# Transboundary Animal Diseases, an Overview of 17 Diseases with Potential for Global Spread and Serious Consequences

**DOI:** 10.3390/ani11072039

**Published:** 2021-07-08

**Authors:** Elizabeth A. Clemmons, Kendra J. Alfson, John W. Dutton

**Affiliations:** 1 Southwest National Primate Research Center, Texas Biomedical Research Institute, 8715 W. Military Drive, San Antonio, TX 78227, USA; jdutton@txbiomed.org; 2 Texas Biomedical Research Institute, 8715 W. Military Drive, San Antonio, TX 78227, USA

**Keywords:** transboundary animal diseases, emerging and re-emerging infections, animal models

## Abstract

**Simple Summary:**

Animals provide food and other critical resources to much of the global population. Transboundary animal diseases are highly contagious or transmissible, epidemic diseases, with the potential to spread rapidly. They have the potential to cause negative socioeconomic and public health consequences. A greater understanding of the factors contributing to disease pathogenesis and spread is needed. Further work is also needed to improve the efficacy and cost of diagnostics and prevention measures for these diseases. This review aims to give a broad overview of 17 transboundary diseases, providing researchers and veterinarians with a current, succinct resource of salient details regarding these significant diseases. For each disease, we provide a synopsis of the disease and its status, species and geographic areas affected, a summary of research models, and when available, information regarding prevention or treatment.

**Abstract:**

Animals provide food and other critical resources to most of the global population. As such, diseases of animals can cause dire consequences, especially disease with high rates of morbidity or mortality. Transboundary animal diseases (TADs) are highly contagious or transmissible, epidemic diseases, with the potential to spread rapidly across the globe and the potential to cause substantial socioeconomic and public health consequences. Transboundary animal diseases can threaten the global food supply, reduce the availability of non-food animal products, or cause the loss of human productivity or life. Further, TADs result in socioeconomic consequences from costs of control or preventative measures, and from trade restrictions. A greater understanding of the transmission, spread, and pathogenesis of these diseases is required. Further work is also needed to improve the efficacy and cost of both diagnostics and vaccines. This review aims to give a broad overview of 17 TADs, providing researchers and veterinarians with a current, succinct resource of salient details regarding these significant diseases. For each disease, we provide a synopsis of the disease and its status, species and geographic areas affected, a summary of in vitro or in vivo research models, and when available, information regarding prevention or treatment.

## 1. Introduction

Animals provide food and other critical resources such as hides and transportation to the majority of the global population. As such, diseases of animals can cause dire consequences, especially disease with high rates of morbidity or worse, mortality. The Food and Agriculture Organization of the United Nations (FAO) and the World Organisation for Animal Health (OIE; formerly the Office International des Epizooties) maintain a list of transboundary animal diseases (TADs). These are highly contagious or transmissible, epidemic diseases, that have the potential to: spread rapidly across the globe, cause substantial socioeconomic losses, and result in negative public health outcomes [1,2].

Transboundary animal diseases are capable of threatening the global food supply through the direct loss of animal protein and products such as milk, or through production deficits from the loss of animal power; reducing the availability of other animal products such as hides or fibers; or diminishing the supply of food or other animal products through loss of human productivity in the case of zoonoses. There are also significant socioeconomic consequences from the cost of control or prevention measures, and from trade restrictions that can result from outbreaks and countries with differing disease status. Thus, there is a high likelihood that these diseases can increase poverty and food insecurity, especially in developing nations that depend heavily on livestock. Unfortunately, TADs are predominantly in low-income areas, thus increasing the significance of the consequences and the difficulty in obtaining funding for control or prevention measures [3,4]. In addition, TADs have the potential for severe public health consequences when humans are also susceptible to the disease; in some cases, these diseases can have high morbidity and mortality in human populations. Finally, the pain and suffering of afflicted animals cannot be discounted.

A greater understanding of transmission, spread, and pathogenesis of these diseases is required to provide better control and mitigate negative outcomes. This will necessitate the development of better characterized in vitro and animal models. Further work is also needed to improve the efficacy and cost of both diagnostics and vaccines. The control and prevention of these diseases rely on rapid diagnostics and/or effective vaccination strategies [5].

**Figure 1:**
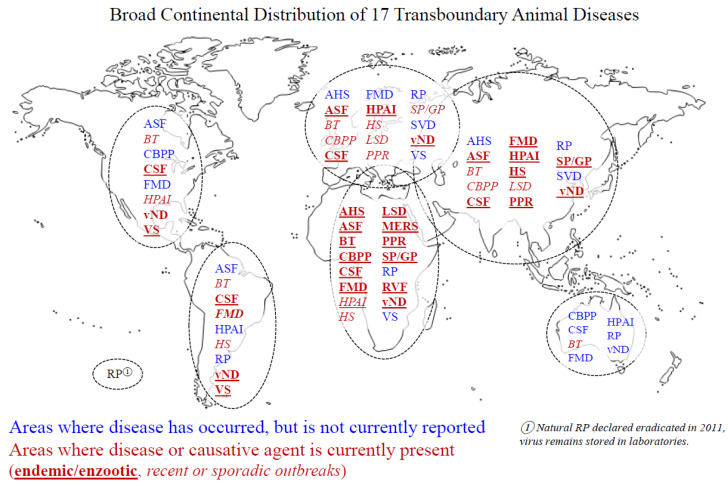
Broad continental distribution of 17 transboundary diseases. This figure displays a general and broad continental distribution of each disease. Due to the potential for these diseases to easily cross borders, the geographic distribution is divided into broad regions rather than being country specific. Abbreviations: African horse sickness (AHS), African swine fever (ASF), avian influenza (HPAI), bluetongue (BT), classical swine fever (CSF), contagious bovine pleuropneumonia (CBPP), foot and mouth disease (FMD), hemorrhagic septicemia (HS), lumpy skin disease (LSD), Middle East respiratory syndrome (MERS), Newcastle disease (VND), peste des petits ruminants (PPR), Rift Valley fever (RVF), rinderpest (RP), sheeppox and goatpox (SP/GP), swine vesicular disease (SVD), vesicular stomatitis (VS).

This review aims to give a broad overview of transboundary diseases, providing researchers and veterinarians with a current, succinct resource of salient details regarding these significant diseases. For each disease, we provide a synopsis of the disease and current status, species and geographic areas affected, a summary of in vitro or in vivo research models, and when available, information regarding prevention or treatment. Table 1 presents a brief overview of each disease, including the causative agent, species generally affected, and common symptoms. Figure 1 displays a general and broad geographic distribution of each disease, including where the disease has historically been found versus where it is currently thought to be present. Due to the potential for these diseases to easily cross borders, the geographic distribution is divided into broad regions rather than being country specific. The following diseases are included, based on the consultation of both FAO and OIE lists: African horse sickness, African swine fever, avian influenza, bluetongue, classical swine fever, contagious bovine pleuropneumonia, foot and mouth disease, hemorrhagic septicemia, lumpy skin disease, Middle East respiratory syndrome, Newcastle disease, peste des petits ruminants, Rift Valley fever, rinderpest, sheeppox/goatpox, swine vesicular disease, and vesicular stomatitis.

## 2. Methods

Literature searches were performed using both PubMed and Google Scholar, with no initial restriction on date range. The following search terms were used, for each disease: Disease, Disease + review, Disease + models. The results were sorted by relevance and the first ten results were selected from each search. Additional results that appeared potentially relevant to the goals of the review were then selected. After the initial search, another search was performed with a date range of 2015 to 2021 to find further, recent results. An additional search was also performed on both PubMed and Google Scholar using the search terms Disease + treatment. Additional references were reviewed as needed, from the reference list of literature found during the initial search. Searches were conducted between December 2020 and May 2021. Articles relevant to the goals of this review, which include providing a synopsis of the disease, sharing information regarding prevention or treatment and summarizing available research models, were selected and cited from the compiled searches for each disease.

## 3. Review

### 3.1. African Horse Sickness

African horse sickness virus (AHSV) causes African horse sickness (AHS) and is a non-enveloped, double stranded RNA arbovirus that belongs to the genus *Orbivirus*, in the family *Reoviridae* [6]. AHSV is divided into nine serotypes (AHSV1-9) [7,8]. The virus is closely related to epizootic hemorrhagic disease virus (EHDV) and bluetongue virus (BTV), the type species of the genus *Orbivirus* [8,9]. The viral genome has 10 segments, numbered 1–10, encoding seven structural proteins (VP1-7), and five non-structural proteins (NS1-3, N3A, and NS4) [10,11,12,13]. These structural proteins are similar to those of bluetongue virus [12], and VP2, encoded by segment 2, is the most important in serotyping and eliciting a neutralizing antibody response [10,12], while VP1, VP4, and VP6 make up the viral transcription complex [10]. Segments encoding the proteins NS1 and NS2 are highly conserved across the nine serotypes. More variable regions between the serotypes encode the proteins NS3, NS3A, NS4, as well as the outer capsid proteins [8,10,11,12]. NS4 has recently been shown to be an important virulence factor, by disrupting JAK-STAT signaling in the innate immune and antiviral response of the host animal [11]. Genome reassortment and recombination is seen in AHSV and plays an important role in the evolution of orbiviruses [12,14].

AHSV can cause a highly lethal disease in horses, and the virus can infect all equine species, with AHSV-9 being less dependent on the zebra reservoir than others [15,16,17]. Other hosts include goats, elephants, camels, dogs, ferrets, and wild carnivores have also shown exposure through antibody responses, but it is unknown what, if any, role they play in the enzootic cycle of the virus [9,15]. The non-equid species surveyed are likely dead-end hosts, or have nonspecific antibody reactions, while dogs may be exposed most often by ingestion of contaminated meat [17,18,19]. Horses appear to be the most susceptible to severe disease, while donkeys and mules tend to have a milder disease but longer viremia [12]. Zebras generally are asymptomatically infected and are presumed to be the reservoir host in parts of Africa [12,17,18,19,20,21]. The disease is non-contagious and is transmitted by at least two species of biting midges, *Culicoides imicola* throughout the range in Africa and *C. bolitinos* also playing a role in South Africa [6,15,16]. These midges can cause spread over local distances of a few kilometers; however, spread over long distances is primarily due to movement of infected mammalian host species, with asymptomatic animals playing a large role in the spread of disease [15,20]. The disease is enzootic in sub-Saharan Africa, and possibly in Yemen and the Arabian Peninsula as well [17]. There have been epizootics of disease in India, Pakistan, Spain, Portugal, and most recently in Thailand [17,22,23,24,25,26,27]. Global warming and the resulting spread of *Culicoides imicola* outside of Africa, has the potential to further spread the enzootic range of this disease [17,27,28,29]. Other *Culicoides* species pose a threat to transmission of the disease in non-enzootic areas [19].

The disease has been known for centuries in Africa [7]. Disease presentation can be peracute, subacute, mixed, or a milder form of the disease known as horse sickness fever [7,16,19,30,31]. The peracute form of the disease is characterized by pulmonary illness, while the subacute form has a cardiac presentation [16]. The disease is largely fatal, with recovery seen primarily in the milder, horse sickness fever, form [7]. The virus infects endothelial cells and monocytes, with viremia that may last as long as 21 days. The clinical form of the disease depends on the infecting strain, with a mixed form of the disease often being seen in horses, while zebras, being the reservoir host, most often present with the milder fever form [15].

There are a variety of methods for the diagnosis of AHS. The gold standard for detecting the specific serotype is by viral neutralization assay [15]. Other ways of detecting the AHSV include molecular methods that include antigen-antibody binding, such as enzyme-linked immunosorbent assay (ELISA), complement fixation, serum neutralization, or polymerase chain reaction (PCR) [12,15,30]. Newer methods, such as real-time PCR (RT-PCR) and gene expression or sequencing to detect specific RNA sequences have been developed to differentiate between the differing serotypes [12,15,31].

The control of the disease is similar to other vector-borne diseases and varies according to location. The OIE can officially recognize countries as being AHS-free upon request after meeting specific criteria, including no cases of infection in the previous two years, no routine vaccination during the past year, and restrictions on imported equids [19,32,33]. Alternatively, a Member Country can apply for recognition as being historically free of the virus [32]. AHS is the only equine disease for which countries can obtain this status [19,33]. In order to prevent spread from areas where the disease is enzootic or epizootic, quarantine of equines moving from these areas should be practiced [6]. Additional measures that can be taken on the host species include vaccination in both enzootic and epizootic areas and stabling overnight in mosquito-proof stalls [6]. Many vaccines are multivalent, as there is limited cross-protection between the nine serotypes and this will elicit a broader immune response [16,34]. The standard method of vaccination is based on live attenuated vaccines (LAV). These vaccines have an inherent risk of reverting to virulence [10,35], they may not prevent infection and African horse sickness fever [36], and they do not allow for differentiating infected from vaccinated animals (DIVA) [10]. This inability to differentiate infected from vaccinated animals results in difficulties in maintaining an AHS-free zone [37]. As a result, there has been recent progress on the development of recombinant and inactivated vaccines, protein, and virus-like particles [10,38,39,40,41,42]. Reverse genetics systems and recombinant techniques are being used to develop new vaccines to target specific antigens of the AHSV, including several viral and nonstructural proteins of the virus, including the capsid proteins VP2 and VP5, as well as NS1, which may elicit an interferon gamma host antiviral response [10,11,12,16,43]. These newer technologies aim to create a DIVA vaccine, which will greatly improve the control and detection of the virus. Finally, exclusion measures in certain AHS-free countries may include culling of positive animals to prevent an epizootic or establishment in the new area [19].

In addition to host-specific mitigation and prevention techniques, strategies to target the vector can also be implemented. These include spaying of insecticides [19], but care must be taken when used near food producing animals [15]. Local strategies, such as the elimination of breeding habitat for the *Culicoides* midges, should also be practiced. This includes the removal of dung and the elimination of mud or pooled water [15].

The socioeconomic impact of AHS in enzootic areas is great. This ranges from low-income communities, where working animals are affected by disease, to racing, sport, and leisure activities where either disease or impediments to movement across regions is affected [33]. The impact on low-income areas is largely due to morbidity and mortality, and affects food security, as well as having ripple effects on poverty alleviation and gender equality. The exact impact is difficult to discern, as diagnosis and reporting is rarely done [6,33]. The greatest potential for financial impact relates to horse racing. The total for this industry amounts to several hundred billion dollars annually [6].

Most vaccine development studies have been done in horses, and most of what is known about the disease is from naturally infected animals. There has been some standardization towards a mouse model of disease. Early work was done in BALB/c mice, by several challenge routes, to compare attenuated vaccine strains of AHSV to wildtype [44]. Recent work has focused on studying AHSV4 in the IFNAR −/− mouse [45,46,47]. These mice lack the type-I interferon receptor, which increases susceptibility to viral infection. Studies have focused on vaccine evaluation and a further characterization of the model. Additional work has been done to characterize a guinea pig model for the evaluation of AHSV vaccines [39,48].

### 3.2. African Swine Fever

African swine fever virus (ASFV) causes African swine fever (ASF) and is a large, enveloped, double-stranded DNA virus and is the only member of the family *Asfarviridae* [49,50,51,52]. The virion consists of a core with a linear genome, internal lipid membrane, an icosahedral capsid, and lipid envelope [49,50,51,53,54,55]. ASFV can be transmitted by direct or indirect contact with infected pigs, as well as by soft ticks of the genus *Ornithodoros*. This includes *Ornithodoros moubata* in Africa, and *O. erraticus* in Europe, which serve to transmit the virus to wild and feral suids, as well as serve as a reservoir of the virus [50,51,56,57]. Due to differences in vector species and natural host reservoirs, the epidemiology of the virus varies on regional scales [52,56]. ASF has been reported in at least 60 countries to date [56,58,59,60,61].

African swine fever was first described in Kenya in 1921 [51,62]. It causes a hemorrhagic fever with mortality rates nearing 100% in domestic pigs and Eurasian wild boar. Mortality can differ in domestic and wild suids according to virus strain [50,53,62,63]. The virus remained limited to Africa prior to the mid-twentieth century, when it spread to Europe, South America, and the Caribbean. A second expansion out of Africa spread to the Republic of Georgia, the Russian Federation, and again into Europe, where it had previously been eradicated, with the exception of Sardinia [51]. It remains enzootic in sub-Saharan Africa, but the recent expansion out of Africa has now spread to Asia, including China in 2018, where half of the world’s pig production occurs [53,64].

ASFV is highly contagious, and transmission can be through direct contact with infected pigs, ingestion of infected meat, exposure to contaminated feces, blood, or urine, or by the tick vector. There are commercially available diagnostic kits based on ELISA or PCR, but use of such tests may be regulated in certain countries [63]. Confirmation can be performed though virus isolation in porcine leukocyte or bone marrow cells, followed by hemadsorption assays [65]. ASFV can be transmitted by fomites and is highly stable in the environment, especially in protein-rich matrices, such as infected wild boar and their carcasses and the meat from infected domestic pigs [64,66,67]. After exposure, the virus infects monocytes and macrophages, and later, endothelial cells. This leads to the classic presentation of death from shock secondary to disseminated vascular coagulation (DIC) [54,68]. Disease presentation can vary from peracute death to persistent infection and depends on virus strain. Clinical signs include pulmonary edema, depression, fever, anorexia, petechiae, cyanosis, thrombocytopenia, lymphopenia, and hemorrhagic lesions [69]. In contrast to domestic and feral pigs, African wild pigs (warthogs and bushpigs) tend to be asymptomatic and likely serve as the reservoir in areas of Africa where the virus is enzootic through a sylvatic cycle with the tick vector [69,70,71]. However, spread of the virus is primarily due to human activities and the movement of infected pigs [64,66].

The ASFV genome encodes between 151 and 167 open reading frames (ORFs), representing more than 160 proteins [54,55,64,72,73]. The virus contains genes for enzymes related to DNA replication and repair, protein modification, and virus–host interactions. ASF viral transcription is independent of host RNA polymerase II, and the virus may replicate in the macrophage cytoplasm [69,72]. The virus is immunomodulatory for both innate and adaptive immune systems through numerous mechanisms [53,69,72,73]. The viral genome encodes proteins that directly inhibit macrophage intracellular signaling as well as intercellular signaling of immune cells. This includes the inhibition of Toll-like receptor 3 signaling (which is the pathway that recognizes infection with dsDNA viruses), and inhibition of the Type I interferon response (also involved in innate immunity against viral infection) [72,73,74,75]. One viral protein, EP402R or CD2v, is structurally similar to host CD2 in that it has two immunoglobulin-like domains, Ref. [53] which inhibits lymphocyte proliferation [72]. The viral protein is responsible for hemadsorption to the infected macrophage [53,72]. Viral protein EP153R resembles NK cell receptors (e.g., CD69), inhibiting up-regulation of MHC Class I expression, Ref. [72] which is the cellular mechanism of presenting foreign antigen to immune cells. ASFV also encodes several genes which inhibit cellular apoptosis through multiple signaling pathways, thus preventing the infected cell from undergoing apoptosis [73].

In spite of the difficulties the virus poses to the development of an effective vaccine, this is currently a very active area of research [64,69,71,76,77,78,79,80]. It is known that pigs surviving acute infection develop long-term resistance to infection by homologous strains of the virus [76,80]. The development of inactivated vaccines produced by numerous methods have all been unsuccessful, and it appears that an efficacious killed vaccine will not be possible [77,79,80]. Attempts at developing a live attenuated vaccine (LAV) have been made through serial passage in bone marrow culture cells, or through using naturally attenuated strains have led to chronic lesions and late disease [79,80]. Serial passage in cell lines, such as Vero cells or COS-7 cells, has led to decreased protection, or inability to replicate in domestic pigs [76]. Attenuated strains may also be developed by the deletion of certain viral genes. A recent vaccine made by deletion of a virulence factor (Pret4Δ9GL virus) was found to be safe in pigs and imparted partial protection from a homologous strain of ASFV as early as 21–28 days [81]. LAVs provide the advantage of being more simple to develop compared to subunit vaccines [78], and they can elicit a host immune response to all antigens present, as opposed to recombinant vaccines with a limited number of antigens [79]. Also, there can be a small window of safety, with virulence at times only depending on the dose of the virus used [80], and attenuated strains will not likely allow for differentiating infected from vaccinated animals [76]. Important factors in developing an acceptable vaccine include, safety, DIVA, regulated and reliable production, and cross-protection, as well as developing formulations that may be used in wildlife, such as bait vaccine formulations [78].

Subunit vaccine technology has been investigated using antigen, DNA, and virus vector based vaccines [71]. One advantage of antigen and DNA-based vaccines is a more favorable safety profile than LAVs [71]. The development of subunit vaccines depend on identifying neutralizing antibodies to viral antigens. Three ASFV proteins appear to be promising in achieving this; p30, p54, and p72, however, attempts at using a recombinant vaccine targeting these antigens provided high titers but was not protective in challenge studies [82]. Other proteins may also provide immunity but attempts to produce a vaccine have resulted in similar results [71,77,79,80]. Vaccines targeting the CD2v protein have been shown to reduce hemadsorption and the virus’ ability to infect monocytes, providing partial protection [79]. Single cycle and replication deficient viruses have promise to provide safe vaccines [77]. Virus vectored vaccines have been based on vaccinia virus Ankara, baculovirus, and alphavirus replicon particles. A combination approach using heterologous prime-boost strategy has also been investigated. By using two vaccine platforms, there is a hope of providing more robust innate and cellular immunity [71].

Due to the lack of an approved vaccine against ASFV, traditional methods of containing and eradicating an outbreak are followed, including movement restrictions and culling [64,81,83]. The early detection and eradication in ASF-free zones is vital. The current outbreak in China is believed to have spread rapidly after pigs from an affected farm were sold to several nearby farms. Given the widespread, decentralized nature of pig farming in China, the long-distance transportation of pigs aided in the dispersion [64,84,85]. Basic biosecurity measures include restrictions on movement and trade of live pigs and raw or treated meat products, as these can remain infective, prohibiting exposure to wild boar, and culling animals in the face of an outbreak. Additional measures include cessation of swill feeding of pigs and wild boar, safe disposal of contaminated products, and restricted zones of at least 3 km and surveillance zones of at least 10 km for pigs and pork products [83,86]. Effective biosecurity is important, as illustrated by outbreaks in Sardinia, Eastern Europe, and the Russian Federation, where backyard farms and small-scale producers that may lack adequate biosecurity measures led to spread of ASFV and long-term outbreaks [67,87,88]. Outbreaks in large commercial farms can have a devastating impact. It was estimated that approximately 800,000 pigs died or were destroyed in Eastern Europe and the Russian Federation between 2014 and 2017, and exports from Poland, Lithuania, Latvia, and Estonia were reduced by US $961 million as a result of outbreaks in 2014 and 2015 [67]. Spain provides a successful model for eradication of the virus. From 1985–1995; Spain instituted a program with several components, including: mobile field teams for control and diagnosis, serological testing of animals, facilities improvements, including barriers and the safe disposal of manure, elimination of all outbreaks and a test and cull strategy to identify carriers, and controlled movement with individual identification of animals. If an outbreak is detected, all pigs within the 3 km protective zone are immediately serologically tested immediately, movement is stopped within the 10 km surveillance zone for 30 days, and animals within this zone are tested no sooner than 30 days after the initial cleaning and disinfection of the affected areas. Once serological data indicate the area is free of ASFV, movement within the zones could recommence, but no movement of live pigs is allowed outside of this zone [86].

Given the complexity and cost of biocontainment studies in large animals and the gaps in understanding the immunology related to ASFV vaccinology, a small animal model could provide benefit in the development of new vaccines and help describe the complex interaction of the virus in host leading to disease. A mouse model showed that a recombinant Newcastle disease virus vaccine expressing the ASFV p72 gene was safe and effective, but previous studies showed a lack of translation when studied in pigs [71]. The development of a small animal model of ASFV that recapitulates the disease and immunology of pigs would likely shorten the time to the development of a safe and effective ASFV vaccine to be used in the control of this important disease.

### 3.3. Avian Influenza

Avian influenza (AI) refers to a group of single-stranded, negative-sense, enveloped RNA viruses of the family *Orthomyxoviridae* in the genus Influenzavirus A [89,90]. Influezna viruses are classified based on two surface glycoproteins, hemagglutanin (HA) and neuraminidase (NA). There are eighteen different hemagglutinin subtypes (H1-18) and eleven different neuraminidase subtypes (N1-11). These viruses can be further classified as low pathogenicity (LPAI) or high pathogenicity (HPAI), by the disease they cause in the domestic chicken (*Gallus gallus domesticus*) [89,91]. HPAI viruses fall into groups with H5 and H7 hemagglutinin subtypes and may result in 100% mortality. However, not all H5 and H7 viruses cause HPAI, Ref. [91] and the molecular difference between LPAI and HPAI may only be one amino acid [89].

All type A influenza viruses were derived from wild birds, mainly waterfowl (Order Anseriformes) and shorebirds (Order Charadriiformes), with the exception of H17N11 and H18N12, which have only been isolated in bats [92,93]. Waterfowl and shorebirds are the accepted reservoir, but typically circulate virus that is LPAI in domestic poultry. These LPAI viruses have been isolated from more than 100 species across more than 25 families [92]. HPAI has evolved from LPAI in domestic poultry, first noted in Italian chickens in 1878, and from that time was known as “fowl plague” [89,91,92]. HPAI had remained a disease of domestic poultry until an H5N1 strain of HPAI was found in domestic geese in China (A/goose/Guangdong/1/1996 lineage H5Nx viruses) and has since caused morbidity and mortality in wild birds as well [92,94]. LPAI viruses have been identified in a wide range of other birds and mammals, including felines, canines, suids, equines, and mustelids [95,96,97]. The local spread and evolution of LPAI viruses can lead to continental-scale distribution [98]. One LPAI currently circulating worldwide is H9N2, which poses a great risk to small scale and family farms, where it may have great socioeconomic effects [94,99,100,101]. While H9N2 viruses were first isolated in Wisconsin, United States, in 1966, the currently circulating virus has developed into many clades that are now enzootic in Asia, the Middle East, and parts of Africa. Since H9 viruses are not reportable, as H5 or H7 are, further spread will be difficult to curtail [94].

Most HPAI outbreaks have been limited in their temporal and geographic impact. Between 1959 and 2019, 15 H5 and 27 H7 (total of 42) conversions to HPAI have occurred worldwide [92]. Of these, all but three were limited in scope. The exceptions include A/goose/Guangdong/1/1996 (H5Nx), Mexican H7N3, and Chinese H7N9 [93,102]. Deaths in wild birds, poultry, and humans have been linked to the Guangdong goose lineage (Gs/Gd). The geographic extent encompasses over 80 countries in Asia, Europe, Africa, and North America [92]. These strains have been detected in migratory birds in China, Mongolia, South Korea, and Japan by 2011. By comparing outbreak records with the satellite tracking of wild birds, and comparisons of whole-genome sequencing of viral samples, it was shown that spread occurred along migratory bird routes [103]. Viruses of this lineage are now enzootic in wild waterfowl and have spilled over into domestic poultry and have evolved into at least 8 different genotypes [104]. At the time of this writing, there are 410 outbreaks of H5Nx HPAI in poultry and 233 in non-poultry, reported to OIE’s early warning system by Member countries. This includes 215 new outbreaks in poultry in Asia, Europe, and Africa, and 79 non-poultry outbreaks in Asia and Europe [105].

The expansion of HPAI beyond a local outbreak depends largely on migratory birds. There are at least nine different types of H5 viruses circulating in wild bird populations that present a risk of developing into HPAI or causing human disease. These are H5N1, H5N2, H5N3, H5N4, H5N5, H5N6, H5N7, H5N8, and H5N9 [104]. This followed the 1996 emergence of the Gs/Gd lineage. Prior to that time, HPAI evolved in domestic poultry and could be eradicated through local means, including the depopulation of affected flocks, preventative culling, vaccination, and controlled marketing [93,106]. In 2009, the H5 gene of the Gs/Gd lineage was found to have integrated into at least six different H5Nx subtypes. These viruses have now been seen to spill over into domestic poultry and “spill back” into wild birds in Asia, the Middle East, Africa, Europe, and North America [106]. Surveillance programs for HPAI have now, more than ever, relied on the detection of potentially HPAI viruses in both domestic and wild bird species.

The surveillance of domestic and wild birds can be done through either live virus isolation or next generation sequencing (NGS) of samples or swabs. The advantage of live virus isolation, which includes inoculation of specific pathogen free (SPF) embryonated chicken eggs, is that it shows that live virus is present in the sample. One disadvantage is that it selects for viruses that grow well in eggs. The advantage of NGS is that it is rapid and sensitive to all virus types, however, it does not provide information on whether the virus is live or replication competent [102]. Sampling of live birds often includes tracheal or choanal swabs along with cloacal swabs. These swabs may then be tested by real-time polymerase chain reaction (RT-PCR), along with NGS, to identify notifiable strains. Isolated virus can be identified through agar gel immunodiffusion (AGID), enzyme-linked immunosorbent assays (ELISA) for antigen, or other immunoassays, or by a molecular test such as RT-PCR [95,107]. HPAI can be confirmed with the intravenous pathogenicity index [108]. An additional method involves serological subtyping of the hemagglutinin and neuraminidase subtypes using antibody inhibition [109].

The regional means of mitigation during an outbreak also includes market closure. This is especially important in China, where poultry farming is done at high densities, with >50% of duck and >80% of goose worldwide production occurs, and billions of birds are sold annually in live bird markets (LBMs), Ref. [110] with the local density of LBMs being one of the greatest predictors of risk [111]. Given this scenario, these locations are important in the surveillance of new outbreaks of disease, which is also true for LBMs across differing economies, such as the marketing of upland birds for private hunting in the US [112].

In addition to surveillance and reporting of outbreaks, the best means of protecting domestic bird farming is through prevention. Transmission occurs through direct or indirect contact with infected birds, though movement, equipment, fomites, and vehicles. Airborne transmission is fairly limited [113]. Biosecurity is the basis of protection at the local level [95,113,114]. Influenza A viruses can be disinfected through the use of bleach, quaternary ammonia, alcohols, aldehydes, acids, and iodine solutions, as well as temperatures greater than 56–60 °C (133–140 °F) [95]. Adequate isolation of the farm along with good biosecurity measures, provides good protection. An additional preventative measure is prophylactic vaccination of the flock. When HPAI was less wide-spread, vaccination was not considered best practice, as eradication by depopulation in these infrequent outbreaks was preferred [113]. Vaccination must be used in conjunction with OIE oversight, including the limited use of vaccines using OIE quality standards in situations where culling is not practical. Vaccination strategies must be used in combination with an exit strategy based on certain criteria to be met [115]. Vaccination is often prohibited in countries where HPAI is not enzootic, and where vaccination strategies have been ineffective or led to antigenic drift in cases of failure [116,117].

Since vaccination using inactivated vaccines does not provide full protection and does not allow for DIVA, non-vaccinated sentinel animals may be used to detect outbreaks. In addition, a vaccine with homologous hemagglutinin to the circulating strain, but differing neuraminidase, allows identification of infection based on serology for NA [118]. Additional methods include serology for anti-NS1 (nonstructural protein-1) or anti-M2e (matrix 2 ectodomain) antibodies. NS1 is only produced in active viral replication and is rarely present in inactivated vaccine. M2e is a viral transmembrane protein that allows for entry into the host cell [113,118,119]. HPAI vaccines which are attenuated Newcastle disease virus (NDV)-vectored for H5 or H7 and are protective against both Newcastle disease and HPAI are being developed. NDV-vectored H5 vaccines are currently approved for use in China and Mexico [120].

There are several animal models of avian influenza A viruses, as they relate to human disease [94,121,122,123,124,125,126,127,128]. However, most animal experimentation on avian influenza, as it relates to birds, is conducted in the host species, with most of the testing being done to evaluate pathogenicity of new strains or vaccine development [129,130]. Other work has shown the pathogenicity of goose-origin HPAI in chickens [131]. Guidance on the performance of studies in avian species, including virus selection and preparation, host selection and monitoring, study design, sampling, and analysis, was recently published [132].

### 3.4. Bluetongue

Bluetongue virus (BTV) causes bluetongue and is a nonenveloped, double stranded RNA virus that belongs to the genus *Orbivirus*, in the family *Reoviridae* [133,134,135]. Transmission is vector borne, via biting midges (*Culicoides*) and the disease is noncontagious. Domestic and wild ruminants are susceptible and, as such, the disease can have a large impact on trade and socioeconomics [133,134,136,137]. The virus has spread over time to be present over a large geographical range, with different serotypes being present in distinct regions [138] with global spread increasing [139]. There are almost 30 distinct serotypes globally (28 officially recognized), with new serotypes identified almost on an annual basis [140,141,142,143]; at least 9 serotypes have spread across Europe in the past few decades [138,144].

Sheep are the primary, significant host, as clinical disease is most frequently seen in sheep [137,145,146]. Cattle are also important hosts, but they usually exhibit asymptomatic infections [146]. However, cattle have been shown to exhibit clinical disease in European BTV-8 epidemics [147]. The role and significance of cattle involvement is complex and changing over time (reviewed in [138]). A wide variety of other wild ruminants are also susceptible, including various species of deer and antelope, and camels [148]. Deer belonging to the *Cervinae* subfamily are less susceptible to disease (red deer may serve as reservoirs), while white tail deer (members of *Capreolinae* subfamily) are more highly susceptible [149,150]. In India, seroprevalence studies have suggested that the following animals are susceptible (listed in order of percent found seropositive, though seroprevalence was found to vary by geographic region): goats (43%), sheep (39%), cattle (38%, though prevalence was 66% when looking specifically at *Bos frontalis,* known as Mithun), buffaloes (34%), and camels (16%), with prevalence varying based on specific region (reviewed in [144]). Furthermore, BTV has been reported in canines [151] and a variety of African carnivores, though the overall significance of this finding requires further investigation [152].

The disease symptoms are broad and depend on many factors, including animal species, virus serotype, and route of infection. Disease symptoms often include lameness, painful hooves, and ulcerations or sores; animals may also develop a swollen tongue, which leads to decreased blood flow and thus a blue coloration of the tongue, giving the disease its name (bluetongue) [145,146]. In sheep, BTV can cause serious disease with overt symptoms such as fever, hypersalivation, swelling, vascular injury and hemorrhage, ulceration, pulmonary edema, muscle necrosis, and possible death. Other animals may present with no symptoms at all [134,145].

Bluetongue disease is a re-emerging disease with major, global economic implications [153]. Economic loss can result from losses in productivity, animal death, cost of control measures, or trade restrictions [154,155]. Surveillance and vaccination programs also increase the financial burden [156]. Many vaccines have been developed over the years (including modified live virus, attenuated BTV, and inactivated vaccines), as vaccination is more feasible than vector control strategies. However, vaccines are not available for all serotypes but new vaccines are being developed (reviewed in [157]).

Animal models are crucial to study the pathogenesis and evaluate vaccination, treatment, and preventative measures. As sheep are the primary host impacted by clinical disease, they serve as a good large animal model and are commonly used to evaluate the immune response and vaccine efficacy (reviewed in [158,159]. The difference in virulence between different strains has also been evaluated in sheep [158]. The characteristics of natural infection in sheep can be recapitulated via intravenous inoculation with infected blood, which causes severe disease that includes symptoms seen naturally [146,160]. In models utilizing subcutaneous inoculation, fever is usually the first clinical sign, followed by viral spread from the lymph nodes, tonsils, and spleen leading to viremia a few days after fever, followed by lesions. In these models, the virus seems to first enter the lymph nodes near where the virus is introduced, and spreads from there to the majority of tissues, via lymphatics or bloodstream. Persistence is not thought to be relevant [146,161,162,163]. A related model has been developed for cattle, using BTV-8. Intravenous or subcutaneous administration of the virus stock results in clinical signs including fever, eye involvement, ulcers, and swelling. Symptoms were more severe and prevalent than traditionally seen with natural infections using other serotypes [164]. The virus is first observed in peripheral blood mononuclear cells (PBMCs) with subsequent spread to the spleen and then most other tissues; spread is temporally similar to what has been observed in experimentally infected sheep. Viral replication also appears to begin in the lymph nodes near the site of infection. Experimentally, adult cattle (not calves) have previously been shown to have a more persistent viremia [165] but this finding has been challenged and is generally no longer accepted [146,162,163].

There are many challenges to large animal models, such as sheep and cattle. Thus, small animal models have also been an important area of research. These models have been especially useful for studying virus replication, characterization, virus evolution, and markers of attenuation [166,167]. As with many viral infections, suckling mice are susceptible to BTV, generally using the common intracranial route [166,167]. Pathogenicity was shown along with infection of various parts of the brain, along with pathology including encephalitis and lesions, similar to what is seen in sheep or cattle fetuses. Very young mice were most susceptible, with animals as young as two weeks being much less susceptible [168]. In addition, interferon α/β receptor knockout mice (IFNAR−/−) can be used to model lethal bluetongue for some serotypes, using intravenous or subcutaneous exposure routes. This model exhibits pathogenesis similar to what is seen in natural hosts and can be used for evaluation of vaccines and the immune response [169,170]. However, murine models that lack an interferon response do not fully recapitulate what occurs in natural hosts, such as cattle [169].

As a large variety of animal species are susceptible, a number of other diverse models also exist. These include: antelope (sub clinical infection, with viremia), deer (severe or fatal disease or subclinical disease, depending on deer species and virus serotype), pronghorn and bighorn (clinical disease), bison (low viremia and few symptoms), and camels (low viremia and few symptoms); reviewed in [158].

Numerous models have been developed over the years, but historical studies have been performed with a variety of different virus stocks, administered via different routes. As with other viruses, intramuscular injection has been commonly used but it is unclear how well this exposure route recapitulates natural infection [171]; symptom development may be artificially impacted by unnatural administration routes [172]. Results can also vary based on serotype, animal species, and differences within species (e.g., age) [158]. These issues can complicate results and emphasizes the need for well characterized virus stocks and well characterized models.

### 3.5. Classical Swine Fever

Classical swine fever virus (CSFV) causes classical swine fever (CSF) and is a small enveloped single stranded positive-sense RNA virus that belongs to the genus *Pestivirus* in the family *Flaviviridae* [83,173,174,175,176]. It is closely related to bovine viral diarrhea virus-1 and -2 (BVDV-1 and -2) of large ruminants and border disease virus (BDV) [175]. The viral genome is a single linear strand with one open reading frame (ORF) that codes for four structural and seven nonstructural viral proteins [83,173]. Virus replication occurs in the cytoplasm, where the viral genome is enclosed in the capsid. The virions acquire a round viral envelope during budding by exocytosis. Naturally occurring strains are non-cytopathic in cell culture [173,175,177]. Important factors in transmission and virulence include attachment to host cells, viral replication, immunomodulatory effects, and inhibition of cell apoptosis [178,179,180,181,182,183,184]. There are three distinct genotypes with three or four sub-genotypes [83,173,175]. These genotypes are serologically related and can be cross-protective [83,175].

The historic origin of CSFV is not entirely clear, but it was first reported in the United States in 1833, where the disease became known as “hog cholera.” When it was first recognized in Europe during the latter 1800′s, it was termed “swine fever,” and later, “European swine fever,” to differentiate it from the unrelated African swine fever (ASF). ASF was first described in Kenya in 1921 but may have been misdiagnosed as classical swine fever prior to that time since the diseases may be similar [51,62,83,176,185]. Today, CSF remains an important disease of pigs worldwide [175,176,186]. There are three recognized presentations of CSF: acute, chronic, and prenatal [83,175]. The virus remains stable, even under transpacific shipping conditions, which poses a risk of transboundary spread from enzootic countries [187]. Diagnosis is made by clinical signs, gross pathology, indirect (serological), and direct (virus isolation, antigen, and nucleic acid) detection of the virus [188,189,190,191,192]. Diagnosis should be made using methods and protocols which are validated according to OIE standards [193], and surveillance is imperative in maintaining a CSF-free zone [188,194]. Inactivation protocols have been described to prevent accidental transmission of the virus by diagnostic samples [195].

Much like BVDV, but unlike ASFV, CSFV can cross the placenta and infect the developing fetus, leading to persistent infection particularly during mid-gestation [83,175,185,196,197]. Experimental data indicate that early postnatal infection with low or moderately virulent strains may lead to persistent infection, immunosuppression, and the inability to detect infection based on serological assays [197]. The disease presentation can be affected by many factors, including the virus strain, route of infection, infective dose, and host immune system [83]. The acute form can vary from fever, lethargy, anorexia, conjunctivitis, enteric lymphadenopathy, respiratory and gastrointestinal disease, and possibly neurologic signs, hemorrhagic fever, and death [83,175,198]. Hemorrhage and thrombocytopenia are seen, including hemorrhagic lymph nodes and kidneys. This may lead to characteristic pale kidneys with multifocal hemorrhage, or “turkey egg” kidneys [199]. Piglets are more profoundly affected, and adult pigs may survive and develop lasting immunity [83,175,198]. The efficiency of the virus to cross the placental barrier is dependent on the virulence of the strain, with medium and highly virulent viruses passing more readily. Piglets become persistently infected, despite immune recognition as indicated by increased CD8+ T-cells and IFN-alpha activation in viremic animals [196]. It is important that persistently infected piglets be recognized to avoid these animals being inadvertently vaccinated, rather than identified and culled from the herd [200]. The chronic form is nonspecific and occurs when animals are not able to mount an effective immune response. It is initially similar to the acute disease but is caused by less virulent strains and progresses to chronic wasting, enteritis, reduced fertility, recurring fever, and invariably fatal while not being hemorrhagic. Animals that are chronically infected will continue to shed the virus until death [83,175,198]. The presentation of the prenatal form is dependent on the gestational age at infection and virulence of the virus strain. The presentation in the sow may be subclinical, but if infection occurs early in gestation, it may result in stillbirth, abortion, mummification, and malformations. During mid-gestation, about 50–70 days, the piglet may be immunotolerant, persistently infected, and survive for several months while shedding large amounts of virus. They then develop the late onset form of CSF, exhibiting poor growth, occasionally showing congenital tremor, and ultimately death [83,175,197,198,201].

CSFV is divided into three genotypes (groups 1–3), each with three to four subgenotypes [173,175,186,202,203,204]. These genotypes are not serologically distinct and provide cross-protection [202]. Traditionally, phylogeny was established based on short fragments of the 5′-nontranslated region (5′NTR) and E2 coding region [173,202]. Recent advancements in sequencing capabilities have led to recommendations of using the entire E2 coding region for more detailed phylogenetic determination [173,202]. There is a geographical pattern of genotype distribution with some overlap, mainly in Asia. Circulating genotypes in the Western Hemisphere are of group 1, group 2 strains predominate in Europe, and group 3 strains are apparently solely in Asia. Group 2 strains are the most prevalent genotypes worldwide and are seen in Europe and Asia [173,205,206].

Immunity most effectively targets the structural proteins E^rns^ and E2, which are involved in virus entry into the host cell [186,205]. There are LAV strains in use worldwide, and these vaccines should be produced in accordance with OIE direction [186,205,207]. Attenuation may be based on mutations in the viral genomic regions encoding the E2 and E^rns^ proteins [208,209,210]. CSFV LAVs are often made by serial passage in either rabbits or cell culture [211]. LAVs include the Chinese C-strain, or Chinese hog cholera lapinized virus (HCLV), the Lapinized Philippines Coronel (LPC) strain, Russian LK-VNIVViM strain, the low-temperature adapted Japanese guinea pig exaltation-negative (GPE-) strain, the French Thiverval strain, and the Mexican PAV strain, among many others [211,212,213]. The C-strain vaccine has been shown to protect against highly virulent CSFV strains within days after vaccination [214]. However, antibodies to natural strains of CSFV in enzootic areas may interfere with this efficacy [215]. In some areas, use of LAVs is cost-prohibitive for local farmers, leading to continuation of outbreaks [216]. Since LAVs elicit a multivalent immune response without the ability to DIVA, there are trade restrictions on animals from areas practicing vaccination with these strains [189,190,192,211,217]. Vaccination with the C-strain has led to selection pressure on the antigenic E2 protein, and possible escape mutants [186,205]. However, this claim is still under investigation [211]. Unintentional use of the LOM (Flc-LOM-BE^rns^) vaccine in a combined CSF/erysipelas LAV in South Korea in 2014 has led to a reemergence of CSF on Jeju Island, which had been a CSF-free zone with a non-vaccination policy for the preceding decade [211,218,219].

Recently, work has focused on developing new DIVA vaccines [212,214,217,220]. One approach is the deletion of the E2 protein in the vaccine strain (C-DIVA strain), which provides a means of differentiation [214]. Another approach is development of recombinant vaccines, which include two licensed products: CP7_E2alf (Suvaxyn^®^CSF Marker, Zoetis, Louvain-la-Neuve, Belgium) in Europe, and Flc-LOM-BE^rns^, in South Korea [212,221]. The deletion of glycosylation sites has shown promise for the development of attenuated vaccine strains [181]. One further approach has been production of a fusion protein, combining the E2 protein to the extracellular segment of the host CD154 molecule, which is licensed as Povac^®^ in Cuba [222,223]. Another fusion protein vaccine combines the E2 protein with the host Fc region of IgG. This vaccine may protect against vertical transmission following a two-dose protocol [223]. Recombinant vaccines have been made, including a pseudorabies vaccine expressing CSFV E2 protein, which has shown protection against both pseudorabies and CSFV challenge [224], and a recombinant Newcastle disease-based vaccine expressing E2 and E^rns^ proteins [225].

As with African swine fever, following strict biosecurity practices is imperative. Control of movement, adequate surveillance, and prophylactic and emergency vaccination are potentially useful in successful eradication and control programs [194,226,227,228,229]. Also, as in African swine fever, additional consideration must be given to the presence of infected wild boar [230,231,232,233,234,235]. Wild boars have played a role in transmission of the disease in both Europe and Asia in recent years. Exposure can be through direct contact, through fomites, or by feeding contaminated food products [233,235]. This exposure can complicate eradication programs and lead to persistence and spread of the disease [231]. Biosecurity measures include fencing of the facility, disinfection of people and equipment, hygiene, baiting wild boar with oral vaccines, and capturing or hunting of the wild boar [231,233,234,235].

Animal experimentation of CSFV is often conducted in pigs [236]. An in vitro model using a 3D collagen matrix was validated to study inactivation of CSFV in natural casings for use in sausage making, which are traded globally and may be a source of transmission. Previously, intestines from experimentally infected pigs had been used to assess inactivation protocols [237]. Cell culture methods have been developed to eliminate the need for using pigs to produce virus for challenge stocks [238]. BALB/c mice have been used to study vaccines against CSFV [239]. However, a well characterized model with established viral concentration, strain, and model species has not been developed. The development of a standard model of CSF could help better define some of the current shortcomings in CSF understanding, including certain aspects of pathogenesis, correlates of protection, and the induction of immunotolerance [177].

### 3.6. Contagious Bovine Pleuropneumonia

Contagious bovine pleuropneumonia (CBPP), also known as “lungsickness,” is caused by *Mycoplasma mycoides* subsp. *mycoides* (Mmm), a self-replicating, pleomorphic bacterium [240,241]. *Mycoplasma mycoides* subsp. *mycoides* is one of 5 pathogenic mycoplasmas, known as the “mycoides cluster” [240]. Previously referred to as the small colony (SC) type of Mmm, this designation was dropped after Mmm large colony (LC) was reclassified as *Mycoplasma mycoides* subsp. *capri* [240]. First described by Gallo in the 16th century, CBPP reached almost worldwide distribution in the 19th century [241,242]. Previously second only to rinderpest as the disease of prime concern in cattle, CBPP remains a significant concern and threat to the livestock industry [243,244]. Following drastic stamping out efforts, CBPP was eradicated in the 20th century from most of the world but continues to plague Sub-Saharan Africa [245,246,247,248,249,250,251,252].

Contagious bovine pleuropneumonia is an acute, subacute or chronic disease that primarily affects cattle (*Bos taurus*, *B. indicus*) and sometimes water buffalo (*Bubalus bubalis*) [241,253]. Hyperacute forms, characterized by sudden death, may be seen at the beginning of outbreaks [240,254]. Serofibrinous pleuropneumonia and severe pleural effusion are seen in the typical acute to subacute form of disease [240,241,253,254]. In chronic cases, subclinical carriers may be seen [240,241,253]. Transmission occurs through the inhalation of aerosolized infected droplets [240]. The role of subclinical carriers in transmission is uncertain [255,256]. *Mycoplasma mycoides* subsp. *mycoides* survives a short time in the environment and is susceptible to most common disinfectants [240]. After an incubation period of generally 3–6 weeks and sometimes up to 6 months, fever, inappetence, depression and labored breathing may be seen followed by coughing, nasal discharge and salivation [240,241,257]. Mortality can reach 75–90% in epidemic outbreak but is usually less than 10% in enzootic regions [240,241]. Recovering animals are weak and emaciated and may develop pulmonary sequestra [241]. Calves may develop carpal and tarsal lesions [240,241]. Differential diagnoses include pasteurellosis (*Pasteurella multocida*), east coast fever (*Theileria parva*), bovine tuberculosis (*Mycobacterium bovis*), *Mycoplasma bovis*, actinobacillosis (*Actinobacillus* spp.), traumatic pericarditis, and hyatid cysts (*Echinococcus granulosus*) [240,241]. Rapid, presumptive diagnosis can be made based on clinical signs and gross lesions seen post mortem [240]. The currently available confirmatory diagnostics include PCR, real-time PCR, complement fixation testing and competitive ELISA [240,243,258]. Immunohistochemistry may be useful in chronic cases [240].

Use of antimicrobials is banned during official eradication programs, but they are commonly used to treat CBPP in Sub-Saharan Africa [240]. There is concern for increasing antimicrobial resistance and potentially increasing the number of animals with pulmonary sequestra, but targeted antimicrobial treatment could play an important role in the control alongside vaccination [240,247]. Lacking a cell wall, Mmm is naturally resistant to beta-lactam antibiotics [240]. Antimicrobial susceptibility testing and therapeutic studies have found efficacy of tetracyclines, macrolides, and fluoroquinolones, and shown resistance to tylosin [240,259]. Administration of the fluoroquinolone Danofloxacin did not result in clinical improvement of diseased animals, but in contact animals showed fewer lesions and less mortality [260]. A case report described clinically effective treatment of acute CBPP in a cow using tetracycline, dexamethasone and vitamin B complex [261].

Vaccination is critical for the control of CBPP in enzootic areas [262]. The currently used vaccines are live attenuated vaccines developed decades ago, that show limited efficacy and occasional severe side effects [263,264]. As additional studies allow for a better understanding of the immune responses, virulence factors and molecular characteristics of Mmm and improved vaccine candidates can be developed [263,265,266,267]. Jores et al. describe research priorities for the development of improved vaccines [243]. In addition to the inadequacies of the current vaccines, vaccination campaigns have been inconsistent [241,268]. Before the causal agent of CBPP was identified by Nocard and Roux in 1898, it had been eradicated from several countries with a variety of strategies including strict control of movement, slaughtering, and vaccination, showing the importance of control efforts beyond vaccination alone [269,270,271]. Additional research is also needed to develop simpler and faster field tests [271]. Particularly while improved vaccines are still under development, a comprehensive control strategy will depend on clear policies, government and public commitment, adequate veterinary services, movement restrictions, robust surveillance, good vaccine manufacturing practices and maintaining high diagnostic laboratory standards [272,273,274,275,276,277,278].

Mathematical models have been developed to evaluate economic impact, transmission dynamics, and the potential impact of various control strategies [256,274,279,280,281,282,283]. In vitro models utilizing bovine lung epithelial cells and a variety of assays have been described [240,284,285]. Bovine respiratory explants from trachea, bronchi and lungs of slaughtered cattle are a promising ex vivo tool for further investigation of CBPP infection [286]. Rodent and rabbit models have been used for some vaccine and virulence studies [285,287]. Mice develop mycoplasmaemia following infection, but they are not a good model of the pathology seen in CBPP [285,288]. Cattle models are costly and can present difficulties reproducing disease, but several challenge techniques have been developed [240,241,288,289,290,291]. Contact infection studies resemble natural infection but require an extra group of diseased animals and result in an unpredictable rate and timing of transmission, making it difficult to compare disease outcomes [290,291]. Endobronchial inoculation of three different strains of Mmm in steers showed two strains (Ondangwa and Shawawa) may be useful for study of subacute and chronic infections, while the third (Gladysdale) more closely mimics the peracute form of disease [289]. Nkando et al. presented nasotracheal inoculation of cattle with the aid of a bronchoscope as an alternative to an endobronchial intubation approach where tube insertion requires sedation of the animal [290]. Repeated aerosol nasal infection of cattle has been reported to closely mirror natural epidemiology in which only a fraction of animals develops acute disease [291]. This approach avoids “overchallenge” that could be seen with direct tracheal or bronchial instillation [291].

### 3.7. Foot and Mouth Disease

Foot and mouth disease virus (FMDV) causes foot and mouth disease (FMD) and is a nonenveloped, single stranded, positive sense, RNA virus that belongs to the genus *Aphthovirus*, in the family *Picornaviridae* [292,293]. FMDV was the first described viral infectious animal disease, based on the findings of Loeffler and Frosch during the late 19th century [292,293]. The virus infects cloven-hooved animals, via a variety of routes, and is highly contagious in susceptible animals. As with other RNA viruses, FMDV has a high mutation rate and exhibits high genetic diversity; there are currently seven recognized FMDV serotypes (i.e., O, A, C, Asia-1, South African Territories (SAT) 1 through 3), each containing distinct genetic lineages [292,294,295].

Prior to the 20th century, FMDV was globally distributed. Extensive eradication efforts over the last century have resulted in a diminished distribution of FMDV and the virus is not currently known to exist naturally in North America, Australia, New Zealand, or the majority of Europe. However, FMDV remains an enzootic problem in South America, the Middle East, and the majority of Africa and Asia. At the end of the 20th century and beginning of the 21st century, regions of Europe and East Asia experienced re-emergences [293,296,297,298]. Extensive epidemiological modeling studies have been performed, but these studies must continue so models can be applied in the event that outbreaks occur in regions previously free of FMDV [299].

While FMDV can infect all cloven-hooved animals, natural infections are most prevalent and significant in domestic livestock such as cattle, pigs, sheep, and goats. Some species of deer (roe and muntjac deer (more severe disease), sika deer (milder disease), and fallow and red deer (subclinical disease) [300,301]) and camelids can also contribute to transmission of the virus and may be significant in instances where they are in close contact with domestic livestock [294,296]. Generally, FMDV infections in wildlife are not significant but African buffaloes (*Syncerus caffer*) appear to be maintenance hosts, which complicates eradication efforts in areas where infected buffaloes are present, as virus elimination and control efforts would likely need to extend beyond domestic livestock [297]. A better understanding of the role of wildlife (for example, African buffalo) is needed to understand the risk of transmission posed by these possible reservoirs [299]. Other animals, and humans, can pose a transmission risk if they become contaminated with the virus (e.g., from aerosolization, fomites, clothing, etc.) and then have contact with livestock. Instances of human infections are rare, often disputed, and difficult to confirm. Thus, direct impact to human health from infection does not appear to be a significant cause for concern (reviewed in [296]).

Transmission is via direct or indirect contact, through several different routes [293,296]. The virus is transmitted most commonly and efficiently via airborne or aerosol spread, especially when animals are in close contact [302]. Spread via aerosol across great distances is possible, though it is rare and dependent on the serotype or isolate involved [303]. Animals can also become infected via breaks in the skin or mucosa. Skin and mucosal infection are less efficient and likely require a higher dose of virus than respiratory infection, though information about infectious dose and route are generally from experimental laboratory studies and natural events are difficult to fully understand [293,296,300,302]. In addition, contact with fomites poses a transmission risk, and a variety of bodily fluids (including semen, urine, and feces) can harbor the virus. Furthermore, milk or other animal products can transmit the virus, which has severe implications for trade; international trade bans can result in economic hardship for countries where the virus is enzootic [302]. Pigs can also become infected from eating food contaminated with the virus, though it is unclear if infection is a direct result of ingestion or from breaks in the mucosa [293,296].

In animals that exhibit clinical illness, fever is generally one of the first symptoms. Followed by vesicle development, on the feet and tongue [292,293,294,296,297]. Vesicles can also appear around the mouth (e.g., snout, muzzle), mammary glands, genital mucosa, or other mucosal or skin sites. Lack of appetite or lameness also occur frequently [292,293,294,296,297]. Viremia is common in animals showing clinical signs. Symptoms can vary based on serotype or strain, and are more severe in pigs and cattle, in comparison to sheep and goats [293,296]. In situations where clinical signs are not as obvious or predominant, diagnosis can be complicated. Furthermore, other viral diseases (such as vesicular stomatitis virus [discussed in Section 3.17] and swine vesicular disease [discussed in Section 3.16] can cause similar vesicles. Thus, laboratory confirmation is often required to differentiate FMD from other possible causes of disease [296].

Mortality from FMD is low [293,296]. Rather, the significant impacts are both direct, from loss of productivity and trade restrictions, and indirect, from control and prevention costs. These losses account for billions of dollars annually. Production losses are most noteworthy in developing areas and cause further issues with poverty and food insecurity. Communities that are especially dependent on livestock are particularly vulnerable [304]. Control programs are also quite costly, but the alternative can be even more detrimental, as evidenced by the re-emergence that occurred after vaccination efforts stopped in Europe [304,305].

For example, an outbreak of FMD occurred in the UK in the early 21st century, resulting in the culling of millions of animals. It is believed that the epidemic originated in sheep that were not showing obvious clinical signs (e.g., small number of lesions, which is common in sheep), thus delaying identification of the problem and allowing it to spread to other animals. FMD afflicted pigs were eventually identified at a slaughterhouse [305]. This epidemic and similar occurrences have further spurred research into effective vaccines against FMD [292].

Currently, there are numerous different vaccines available (reviewed in [295,306]). The first vaccine utilized inactivated virus and was used during the middle of the 20th century, mainly to vaccinate cattle in various parts of northern and western Europe [295,306]. The source of the virus used in the vaccine has changed over time (e.g., animal derived, cell culture derived from different cell types) and the inactivation procedure has been refined. Vaccination efforts using inactivated virus resulted in high success and FMDV eradication in Europe, such that vaccination was stopped in the last 20th century [292,295].

However, if vaccinated animals are only partially protected, they may be able to support viral replication, thus posing a risk of infection to other animals [307]. As such, certain inactivated vaccines also rely on removal of nonstructural proteins to maintain only antigenic portions [306]. Another challenge is related to the genetic diversity of the virus; antigenic variation is one of the main barriers to widespread and efficient control via vaccination. It is generally accepted that infection or vaccination against one serotype does not confer protection to other serotypes, so many vaccines are now targeted at more than one serotype. Unfortunately, even within one serotype, vaccination does not always confer protection against all strains within the serotype [306]. Further work is also needed to determine the best targets for vaccines, along with efficacy testing to determine the feasibility of using vaccine control in enzootic countries [299]. Vaccination efforts can be further complicated by various cultural and socioeconomic factors. Due to extensive costs associated with vaccination and lack of confidence in efficacy, it can be difficult to gain acceptance to control programs in some areas. Understanding the cultural and socioeconomic aspects of FMD control and maximizing local community involvement in control programs is essential [299].

A wide variety of cell culture models exist for studying the basic biology of FMDV as well as more complex topics such as persistence. Primary bovine thyroid (BTY) cell cultures were historically used for the isolation and cultivation of FMDV, but immortalized cells lines are more commonly used due to the challenges of working with primary cell lines [308]. Baby hamster kidney fibroblasts (BHK-21) and pig kidney (IB-RS-2) cells are commonly used immortalized cell lines [308], though genetic variability is, unsurprisingly, common after virus passage in cell culture [309]. More recently, fetal goat tongue cells (ZZ-R 127) and fetal porcine kidney cells (LFBK-αVβ6) have also been evaluated for use in isolating and cultivating FMDV, particularly porcine derived strains [310]. Numerous in vitro models have also been evaluated for studying FMDV persistence, including Madin-Darby bovine kidney (MDBK) [311], primary bovine pharynx tissue (PBPT) derived cells [312], the aforementioned BHK-21 or IBRS-2 cells [313], and more recently, multilayer cells from the bovine dorsal soft palate (DSP) that avoid some of the challenges associated with other primary cells lines [314].

Large animal models present multiple challenges in general, but especially when working with high containment pathogens such as FMDV. Further challenges are presented by incomplete knowledge about large animal host immune systems and a lack of appropriate reagents required for immunological studies. The guinea pig and suckling mouse model have been widely used historically, and the guinea pig model remains an essential small animal model for studying FMD. The guinea pig recapitulates many of the clinical symptoms seen during natural infection, exhibits a measurable antibody response, and mortality rates are low. As such, guinea pigs are used to study basic biology of the virus and pathogenesis, along with production of antiserum, and for countermeasure efficacy studies [315]. Natural hosts such as cattle and pigs are used when feasible and have been useful in informing on many aspects of FMD, including transmissibility, infectious dose, pathogenesis, and immune response [296,315].

### 3.8. Hemorrhagic Septicemia

*Pasteurella multocida* (PM), a gram-negative, non-endospore forming coccobacillus is the causative agent of hemorrhagic septicemia (HS), an acute, fatal and septicemic disease of cattle and buffalo [316,317]. Like other members of the family *Pasteurellaceae*, *Pasteurella* spp. are prevalent in vertebrate animals and frequently found as commensal organisms in the oral, nasopharyngeal and upper respiratory tract [318,319]. Many are opportunistic pathogens [318,319]. *Pasteurella* spp. can be passed from animals to humans through bites or nasal secretion, with PM being the most common zoonosis [316,319]. Bacteremia and life-threatening sequelae may be seen in humans with underlying disease or immunosupression [316,320]. With growing concern regarding emerging or re-emerging infections of zoonotic origin, *Pasteurella* spp. have major implications for both human and animal health [319,321,322,323]. *Pasteurella multocida*, first shown to be the causative agent of fowl cholera by Louis Pasteur in 1881 also contributes to swine atrophic rhinitis [318,324,325]. *Pasteurella multocida* is divided into 5 capsular serogroups A, B, D, E, and F with 16 serotypes based on LPS antigens [325,326]. Isolates of PM that cause HS fall into 2 groups, Asian and North American origin (serogroup B) and African origin (serogroup E) [327].

Although seen most commonly in cattle and buffalo, other species may potentially be affected including deer, swine, elephants, rhinoceros, and antelope [328,329,330,331,332]. Outbreaks may be associated with wet, humid weather [328]. Clinical signs include fever, edematous submandibular and brisket swelling, respiratory distress and profuse mucopurulent or bloody nasal discharge [328]. Acute disease, characterized by sudden death within 24 h of onset may be the first indication of an outbreak [317,328]. Subacute forms of disease are often associated with edema and longer, chronic courses may involve rapid, painful breathing and nasal discharge [317]. Nervous system involvement is rare, but has been reported2 [333]. Carrier states are also possible [317]. In enzootic areas, most deaths are in older calves and young adults [317]. Transmission occurs through inhalation of nasal secretions or exhaled droplets from infected animals [334]. Hemorrhagic septicemia has the potential to cause mass mortality events with up to 100% mortality [317,331,335,336]. In 2015, in Kazakhstan, over 200,000 Saiga antelope, representing over 60% of the global population of a critically endangered species, died from HS over a period of three weeks [331]. Unusual high humidity and temperature in the days preceding the event illustrate the potential contribution of environmental changes to extreme disease events [331,335,336].

The differential diagnoses for sudden death caused by HS include lightning, snakebites, blackleg and anthrax [317]. Post-mortem findings associated with HS include subcutaneous edema, fibrinous pneumonia, pericarditis, sub-serosal hemorrhage throughout the body, and blood-tinged fluid in the abdomen and thorax [317,337]. Laboratory confirmation can be made by the identification of gram-negative, bipolar, pleomorphic bacteria in blood smears [317]. While generally easy to isolate pure culture from fatal cases, it can be difficult to isolate in field screening for carriers [338]. Polymerase chain reaction is a rapid and sensitive tool for species and type identification [338]. Loop-mediated isothermal amplification has been shown to be feasible for rapid DNA and RNA detection [339]. Recombinase polymerase amplification (RPA) with lateral flow dipstick (LFD) has the potential to be an effective and practical onsite diagnostic [340]. Due to the ease of obtaining a definitive diagnosis through isolation and identification and the development of rapid molecular diagnostics, sero-diagnosis is not usually needed [317]. Use of an indirect ELISA with higher specificity and sensitivity than indirect hemagglutination assay has been reported [325].

Antimicrobial has limitations including cost, low efficacy once clinical signs appear, and a possibility of failure due to resistance [317,341,342,343]. Ceftiofur, enrofloxacin, or gentamicin may be effective emergency treatment options until susceptibility is known [342]. In enzootic areas, vaccination is the only practical prevention method [317]. The first prophylactic HS vaccine was killed (0.25% Lysol inactivated-broth) and offered 6 weeks of immunity [344]. Subsequent live attenuated vaccines were developed and novel acellular (subunit, recombinant and DNA) vaccines are under development [343]. There are several commercially available vaccine formulations, but broader protection and longer lasting immunity are needed [317]. The optimization of conditions for in vitro PM growth is important for maximizing vaccine production and quality [345,346]. Availability of multiple PM genome sequences will help better understand PM pathogenesis and host immunity, contributing to the development of new vaccine strategies [321,322,323]. In addition to the need for a highly effective, affordable vaccine, control depends on public awareness, good husbandry practices, legislation to control animal movement and responsible use of chemotherapeutic agents [317].

Mathematical models to evaluate outbreak data and potential intervention strategies have been described [347]. In vitro assays have been performed using macrophages and aortic endothelial cells [348,349,350,351]. Mouse models can play an important role in investigation of pathogenesis and vaccine development [317,351,352,353,354,355,356,357,358,359,360,361,362,363]. Rats with or without immunosuppression have also been used to explore pathogenesis and novel vaccines [349,350,351,364,365,366]. Rabbits are occasionally used for vaccine evaluation and, along with mice, were recently used to evaluate a novel phage lysate marker vaccine along with a DIVA ELISA [358]. Goat challenge models have been described [353,354]. Experimental challenge and vaccine assessment has also been established in dairy cattle and buffalo [367,368,369,370,371].

### 3.9. Lumpy Skin Disease

Lumpy skin disease (LSD) virus is a double standed DNA virus of the genus Capripoxvirus, family *Poxviridae*, which causes acute or subacute disease in cattle (*Bos indicus* and *Bos taurus*) and water buffalo (*Bubalus bubalis*) [372]. Goats and sheep may be experimentally infected [372]. Several wildlife species have been shown to be susceptible or seropositive, but the role of wildlife in LSD epidemiology is not well understood [372]. Diagnosed for the first time in Zambia in 1929, by 1944, LSD had spread to South Africa [373]. Enzootic in most African countries and some countries in the Middle East, LSD has also expanded into eastern Europe, Russia, and China [372,374,375]. Lumpy skin disease threatens international trade and could be used as an economic bioterrorism agent [372]. Incubation in experimentally infected animals is between four and seven days, but may be up to five weeks in naturally infected animals [376]. Clinical signs include lacrimation, nasal discharge, inappetence, enlarged lymph nodes, fever, drop in milk production, lameness, nodular skin lesions, and sometimes death [372,376,377]. Skin lesions are firm, slightly raised, circumscribed nodules usually on the neck, legs, tail, and back [372]. Skin lesions cause permanent damage to the hides [378]. Ulcerative corneal lesions and subcutaneous infections may develop [376]. Common secondary complications include pneumonia, mastitis, and orchitis [372,376]. Morbidity is usually approximately 10% and morbidity between 1–3% in enzootic regions [377]. An outbreak in a large Holstein cattle herd saw 12% mortality in adult animals and clinical signs were much more severe in Holstein cattle than indigenous breeds [379,380]. Stress associated with milk production and higher than normal ambient temperatures may have contributed to disease severity [380].

Lumpy skin disease virus may be viable for long periods in the environment [377]. Transmission is primarily vector borne and is most likely mechanical [372,381]. Most likely vectors include blood sucking arthropods such as stable flies (*Stomoxys calcitrans*), mosquitoes (*Aedes aegypti*), horseflies (*Haematopota* spp.) and hard ticks (*Rhipicephalus* and *Amblyomma* spp.), but further studies are needed to better understand vector transmission of LSDV [381,382,383,384,385,386,387]. Most oubreaks occur in summer when arthropods are most active [372,377]. Direct transmission has been rarely reported [372,388]. Prolonged detection in semen and testes raises a concern for possible spread by recovered or subclinically infected bulls [389,390,391]. Introduction of new animals and communal grazing and watering are associated with higher risk of LSD occurrence [392].

Differential diagnoses include pseudo-LSD, malignant catarrhal fever, bovine papular stomatitis, pseudo-cowpox, vaccinia, cowpox, foot and mouth disease, bovine viral diarrhea, dermatophilosis, insect or tick bites, nesnoitiosis, rinderpest, demodicosis, hypoderma bovis infection, photosensitization, urticaria, cutaneous tuberculosis, and onchocercosis [372,393]. Post mortem findings include nodules throughout the lungs and gastrointestinal tract and lung edema and congestion [372]. Pathognomonic histopathologic findings include eosinophilic intracytoplasmic inclusion bodies in keratinocytes and ballooning degeneration of spinosum cells [372,377]. Vasculitis and necrosis may be seen in deeper tissue layers [377]. Diagnosis is primarily clinical with PCR confirmation. Various PCRs have been developed for different aims such as detecting all capripoxviruses, genus-specific detection, and differentiating virulent and vaccine strains [372,394]. As described in an overview of LSD diagnostic techniques, electron microscopy, virus isolation, virus neutralization and serological techniques have also been utilized [372].

Treatment is symptomatic and targeted at preventing secondary bacterial complications [377]. Vaccination along with movement restrictions is the only effective method to control the disease in enzootic areas [372]. Commercially available vaccines are live attenuated [372]. Both homologous (Neethling LSDV strain) and heterologous (*Sheeppox virus* and *Goatpox virus*) vaccines can be used, but heterologous vaccines may not provide complete immunity [372]. A newly developed inactivated vaccine has demonstrated safety and efficacy in the field [395,396]. Calves from infected or immunized dams should not be vaccinated under 6 months old to prevent maternal antibody interferences [393]. Investigations of reported vaccine breakdowns or failures have identified various causes including vaccination of animals already incubating disease, confusion with pseudo-LSD, and infrequent or improper use of the vaccine [373]. Capripoxvirus distribution seems to be expanding, largely due to the economic effects of the Covid-19 pandemic, sanctions in enzootic regions, increased illegal trade and global climate change [372]. Elimination of the disease is likely to be difficult because of arthropod vectors, but controlling the spread may be achieved by accurate and timely diagnosis in enzootic areas, homologous strain vaccination, vector control, animal movement restriction and testing of bulls used for breeding [372].

Several epidemiologic and mathematical models have been described to help understand outbreaks, transmission risks and design surveillance and control programs [397,398,399,400,401,402,403,404,405,406]. In vitro growth for assessment and comparison of viral strain characteristics may be achieved on lamb kidney/testis cells and goat ovarian cells [407]. There have been several recent descriptions of experimental LSD infection in cattle and use of mice for immunogenicity studies [390,407,408,409,410].

### 3.10. Middle East Respiratory Syndrome

Middle East respiratory syndrome coronavirus (MERS-CoV) is an enveloped, single stranded, positive sense RNA virus that belongs to the genus *Betacoronavirus* (lineage C), in the family *Coronaviridae* [411]. This novel coronavirus was reported in 2012 in Saudi Arabi; the initial patient presented with severe respiratory disease including pneumonia and later developed renal failure [412]. Subsequent research shows the virus to be enzootic in camels in the Arabian Peninsula and East Africa, and it is likely bats served as the original reservoirs [413,414]. The virus has since spread to a number of countries and exhibits high mortality rates [413]. Human cases arise from contact with infected camels [415,416]. Human to human transmission is infrequent, but still a cause for concern leading to a need for quarantine in instances of human infections [414]. Limiting zoonotic spread from camels to humans is a crucial step in control. Some have suggested camel vaccination as the best course of action for control, and suitable vaccines are being developed [417]. Experimentally, other animals such as alpacas, pigs, sheep, goats and horses can become infected, though viral shedding is limited in the majority of laboratory infected animals (reviewed in [414]). Animal models have been generally limited, and mainly focused on the macaque [418,419,420].

Ten years before the identification of MERS, in 2002–2003, severe acute respiratory syndrome coronavirus (SARS-CoV) was responsible for a large outbreak that began in Asia and spread to North America, South America, and Europe. Research into these viruses and their zoonotic potential has been ongoing since their identification. However, beginning in 2019, a novel coronavirus (SARS-CoV-2) sparked a global pandemic [421]. Research into SARS-CoV-2, and the related SARS-CoV and MERS-CoV, has grown exponentially since the start of the pandemic. Thus, further in-depth details are better covered outside the scope of this review. Recent reviews provide more in-depth information and discussion of MERS-CoV in the context of the SARS-CoV-2 pandemic [413,422,423,424].

### 3.11. Newcastle Disease

Newcastle disease virus (NDV) is an enveloped, negative sense, single stranded, non-segmented RNA virus with helical capsid symmetry. There are nine serotypes present in the family *Paramyxoviridae* and the genus *Avulavirus*. The virus is the causative agent of Newcastle disease (ND) [425,426,427,428,429,430]. Nomenclature for isolates follows the convention adopted for Influenza A viruses, in that the isolate is named by serotype, species from which it was isolated, geographical location, reference number or name, and year of isolation [429]. Disease presentation depends on a number of factors, including the strain of virus and the species, age, and immune status of the host [425]. NDVs have been classified into five pathotypes related to the disease presentation that typically occurs in domestic poultry: viscerotropic velogenic, neurotropic velogenic, mesogenic, lentogenic, and asymptomatic enteric [425]. Viscerotropic velogenic viruses cause sudden death with intestinal hemorrhage. Neurotropic velogenic viruses cause high mortality following acute respiratory and neurological disease, but usually lack intestinal pathology. Mesogenic viruses cause respiratory and neurologic disease, but low mortality. Lentogenic viruses cause mild respiratory infections, and have been used as vaccine strains [425]. Asymptomatic enteric viruses cause infections, where virus replication appears to be primarily in the gastrointestinal system. There can be overlap in disease presentation in a single monotypic outbreak [425]. The classification of pathotype is often done by either mean death time (MDT) in 9–10 day old embryonated chicken eggs, or by the intracerebral pathogenicity index (ICPI) in 1 day old chicks [428]. The molecular means of determining pathotype include identifying the amino acid sequence of the fusion (F) protein cleavage site [428,429,431,432]. Viral entry into the host cell occurs through attachment by hemagglutinin-neuraminidase (HN) and virus and cell membrane fusion by the F protein [427,430,433], and both HN and F are viral membrane proteins [430]. A virulence (V) protein has been shown to antagonize type-1 interferon (IFN-1) response and may also play a role in host specificity [431]. Certain comorbidities, such as coinfections or environmental factors, can lead a milder strain to present as a more virulent disease [425]. A disease outbreak is usually accompanied by depression, diarrhea, prostration, head and wattle edema, neurologic symptoms including paralysis and torticollis, and respiratory symptoms [425].

Newcastle disease gets its name from Newcastle-upon-Tyne, England, where in 1926, there was an outbreak that was concurrently described on the island of Java, now in Indonesia [427]. In contrast to highly pathogenic avian influenza (HPAI), ND is enzootic in some areas of the world, where disease can have a major impact on poultry production and small community or family flocks [426]. Assessment of the immune response is often evaluated by the hemagglutination inhibition (HI) test, utilizing a panel of antigens and controls [425,431]. While all viruses causing ND are of one serotype, avian paramyxovirus type 1 (APMV-1), there are 19 genotypes known [428]. Genotypes are based on the F gene sequence [428]. Viruses are divided into class I and class II, with class I viruses representing one genotype with three subtypes [428,432]. Class I viruses are largely isolated from wild birds worldwide [428,432]. Class II viruses are comprised of the other 18 genotypes, I–XVIII [428,432]. Genotypes III, IV, V, and VI are typically considered pathogenic to chickens [428]. There have been four historic panzootics of NDV, and the current panzootic is due to genotype VII viruses [428]. Recent work in Bangledesh has identified the circulating NDV in that country as class II genotype XIII, with a possible new variant evolving away from that genotype [434].

Given the importance of family and community flocks in providing global food security, the elimination of Newcastle disease could greatly increase production efficiency in the areas in which it is enzootic [425,426]. One report from 1992 estimated that 90% of community chickens in Nepal die each year from Newcastle disease [426]. Recently, 45% of 1374 chickens sampled, and 96% of 70 villages, were found to be seropositive for NDV in rural farms across three provinces of Northern Iran [435]. Another study found that 57.1% of sampled flocks in Oman, with a total of 33.8% of all chickens sampled, were seropositive for NDV [436]. Vaccination strategies could help in resource-poor rural communities, but the vaccine should be part of a sponsored program to be worth the risk for farmers that tend to adopt new strategies only when they are low risk and there is an obvious return for investment of time and money [437]. Vaccine programs are often decided at the national level, in accordance with OIE guidance [431]. The success of these programs increases when working with farmers to deliver the vaccine in a preferred form [438]. When epizootics of ND lead to high mortality in free-range flocks, there is currently little incentive to put resources into the care of the chickens. When programs can show reduced mortality and improved food security, they are more likely to be adopted by local communities [438,439]. Contributing factors to the incidence of ND include flock size, isolated and confined housing, multi-aged flock mixture, screening of birds, access to ND vaccination, ND awareness, distance to service providers, and access to training and extension services [440,441]. There are reports of vaccine failure, with vaccinated birds showing disease and mortality after exposure to virulent NDV in natural settings [442,443], but this could not be reproduced experimentally following daily challenge for ten days, with no morbidity or mortality seen at 14 days post-challenge [443]. It may be that current vaccines do not appear to be thermostable in the range of 51° to 61 °C, a consideration when planning vaccine strategies in rural areas [444]. Development of thermostable vaccines is a current area of study [442,445,446]. Inactivated, or killed, vaccines have been developed, and these may be given in ovo. This has been shown to provide protection from morbidity and mortality in challenge studies [447]. One disadvantage of many killed vaccines is the potential for a weak cellular response, thus requiring priming with live or other vaccine types [448]. Certain nonpathogenic lentogenic natural strains have been considered for use as vaccines since there is cross protection against all pathotypes of NDV. These can be delivered by a number of routes, including directly, such as oculonasally or by nebulization [449]. Live attenuated vaccines are traditionally produced by serial passage in specific pathogen free (SPF) embryonated chicken eggs, which requires specialized facilities and is resource intensive [431,450]. An alternative means of production is through cell culture [450]. Recent work has been done to produce newer vaccines based on modern molecular techniques [431,448,451]. These recombinant vaccines often use either nonpathogenic or replication deficient virus as the delivery vehicle for an antigen against the disease of choice [120,452]. An example of the former approach is to use NDV as a vaccine platform for HPAI by insertion of the H5 or H7 gene. These vaccines have the potential to protect against infection from both important viruses of domestic poultry [120]. This approach has also been used to develop a vaccine against low pathogenicity avian influenza by incorporating the H9 antigen [445]. An example of a replication deficient virus being developed as a platform for a NDV vaccine is the use of an adenovirus delivery of NDV F protein (adeno-F) [452].

NDV infects a wide range of animals, from reptiles to mammals [425]. It is also known to be zoonotic, usually causing a self-limiting conjunctivitis with no long-lasting effects [425,429]. As many as 241 species of birds have been shown to be susceptible to infection, but the viruses seen in wild birds are mostly of the asymptomatic lentogenic enteric pathotype when infecting chickens [425]. NDV has been isolated in caged pet and zoo birds, as well as wild birds, including double-crested cormorants (*Phalacrocorax auritus*), all types of domestic poultry, racing and show pigeons, pheasants, ostriches, and captive falcons [425,429,432,453,454,455,456,457]. NDV has also been isolated from domestic mink, where it caused encephalitis, hemorrhagic pneumonia, and death [458]. Risk of introduction in areas free of virulent NDV could be associated with legal or illegal movement of birds and animal products and feed, as well as spillover from wild species and transformation to virulence [457,459,460]. Once in a new area, spread has been linked to movement of live birds and poultry products, people and equipment, contaminated food and water, and contact with other animals [429].

Much of the current literature on NDV focuses on the use of the virus as a vaccine platform or gene delivery system for humans and other animals [461,462,463]. This is due to its broad range of species which the virus can infect. Many RNA viruses have been used in this way, including retroviruses, lentiviruses, alphaviruses, flaviviruses, rhabdoviruses, measles viruses, and picornaviruses [462]. NDV is able to infect a broad range of species, but are very safe due to the limited ability to cause disease in these species, while still eliciting a strong immune response [464]. Through various techniques, including reverse genetics and recombinant nucleic acid techniques, NDV-vectored vaccines have now been made for a variety of human and animal pathogens, including HPAI and LPAI, African swine fever virus, vesicular stomatitis virus, West Nile virus, bovine herpesvirus-1, canine distemper virus, rabies virus, simian immunodeficiency virus (a model of HIV), enterovirus 71, Rift Valley fever virus, Nipah virus, Ebolavirus, severe acute respiratory syndrome coronavirus (SARS-CoV-1), and more recently SARS-CoV-2 [120,445,461,462,463,465,466,467,468,469,470,471].

Gene therapy uses of NDV have been directed towards various types of cancer and hereditary diseases [462]. This field began in the 1990s and had early setbacks, including the death of a young patient being treated for a nonfatal genetic disease and the development of leukemia in severe combined immunodeficiency (SCID) patients [462]. In addition to providing a platform for gene delivery, NDV has the ability to replicate in tumor cells [462] and can replicate up to 10^4^ times faster in human cancer cells than in nonneoplastic human cells [472]. This ability to replicate in tumor cells has been coupled to tumor suppressor and immunomodulatory genes, such as tumor necrosis factor (TNF), tumor necrosis factor-related apoptosis inducing ligand (TRAIL), interferon-alpha (IFN-α), interferon-gamma (IFN-γ), and interleukin-2 and interleukin-15 (IL-2 and IL-15) [462,473]. Many of these recombinant viruses take advantage of NDV’s natural oncolytic activity, which is based on apoptosis, necrosis, or autophagy [472]. A proinflammatory response is triggered by NDV-HN, which drives IFN-1 simulation of TRAIL and activates natural killer (NK) cells, monocytes, macrophages, dendritic cells, and primes antigen-specific T cells and CD8+ T-cell proliferation [472,474,475]. NDV-vectored therapies are in human clinical trials for prostatic carcinoma, hepatocellular carcinoma, gastric carcinoma, colorectal carcinoma, and melanoma [462,475]. Research into other cancer types has been done in cell culture or in mouse models using immortal cell lines [151,476,477].

Most of the research into NDV is done as viral challenge models in chickens or in ovo using SPF embryonated chicken eggs, but the strain of chicken and the precise definition of SPF are not often disclosed [444,478,479,480,481,482,483]. The most commonly reported strain of chicken used is the White Leghorn, along with others such as White Rock and Isa Brown layers [120,447,484,485,486]. It is known that differences in the innate immune response in differing breeds of chicken can have drastic effects on the response to infection [487], making this important in describing the model. Some sources that define SPF describe subjects as being serologically or RT-PCR negative for NDV [478,480]. The virus strain and challenge dose of NDV is usually given and is often the experimental variable, but there is not a standard strain and dose that is used as a model of ND [478,485]. A common vaccine strain used in studies is the LaSota strain [430,450,452,480,483,488,489]. Other host species are also used, including Japanese quail (*Coturnix coturnix japonica*) and pigeons (*Columba livia*) [480,485].

### 3.12. Peste des Petits Ruminants

Peste des petits ruminants (PPR), is a highly contagious, devastating viral disease of domestic and wild ruminants, primarily affecting goats and sheep [490,491]. Peste des petits ruminants virus (PPRV) is a negative sense single stranded-RNA morbillivirus, of the family *Paramyxoviridae*, that is closely related to other members of the genus such as rinderpest, measles, and canine distemper [492]. Although only recognized as a completely distinct disease for approximately 40 years, phylogenetic morbillivirus data suggests PPRV has been in circulation as long as rinderpest virus [493]. In naïve populations of sheep and goats, morbidity and mortality can be greater than 90% [494]. In enzootic areas, morbidity and mortality vary between 10–100% [495]. First identified in Cote D’Ivoire in the 1940s, PPR has since been seen in North and Central Africa, the Middle East, and parts of East Africa, Asia, and Europe [492]. The affected countries are home to 68% of the world’s small ruminant population [490]. An investigation of prevalence of PPR in sheep and goats from 1968 to 2018, showed 40.99% prevalence in Africa and 38.43% prevalence in Asia [496]. Cattle, camelids and a wide range of wild animals and unusual hosts such as pigs, are considered susceptible with varying reports of morbidity and mortality [497]. Experimental infection has shown cattle are susceptible and can display clinical signs [497]. Large ruminants may have a role in transmission or, if dead-end hosts, may be of value for surveillance [497,498,499,500].

Peste des petits ruminants virus is easily transmitted by direct contact with secretions and excretions from infected animals or contact with fomites [492]. The main entry route is respiratory [501]. Peste des petits ruminants virus is a lymphotropic and epitheliotropic virus [502]. Stages of disease include incubation, prodromal, mucosal, diarrheal and recovery if non-fatal [503]. The incubation period is generally 3–9 days [493]. Clinical signs include inappetence, emaciation, depression, fever, diarrhea, nasal and ocular discharge, pneumonia and erosive and necrotic stomatitis [492,504]. Fatal cases usually die between 5–12 days after disease onset [493]. Pregnant animals may abort and transient immunosuppression can make animals susceptible to an activation of latent or new infections [493]. Goats may be more susceptible to severe disease than sheep [492]. Pathognomonic histopathologic findings include multinucleated giant cells and cytoplasmic and/or nuclear eosinophilic inclusion bodies [502]. Differential diagnoses include rinderpest, goatpox, bluetongue, contagious pustular stomatitis, contagious caprine pleuropneumonia, pasteurellosis, FMD, heartwater, coccidiosis, poisoning, and Nairobi sheep disease [501,502]. Co-infections of PPR and goatpox have been reported [505,506]. The integration of tests and improved molecular tools may help with the rapid and accurate identification of enzootic and outbreak PPR [507,508,509]. Pen-side tests and non-invasive sample techniques may improve diagnosis in remote settings and wildlife [492,507,510,511].

There is no specific treatment available, but drugs that control bacterial and parasitic complications may decrease mortality [512]. Experimental work has been performed with antiviral treatment and herbal medicines are widely used in field treatment [513]. Despite the availability of an efficacious and cheap live-attenuated vaccine, the virus has continued to spread [514]. The development of a vaccine allowing DIVA would reduce the time and cost of serological surveillance [515]. Recombinant vaccines may overcome the thermolabile and lack of DIVA limitations of current live attenuated vaccines [516]. Genetically engineered live vector vaccines are also promising candidates that can be developed to be multivalent and activate both cellular and humoral immunity [517].

Peste des petits ruminants is currently targeted for global eradication by 2030 by the PPR Global Eradication Programme [490,518]. Factors that favored the eradication of Rinderpest such as one serotype, availability of a safe vaccine that confers long immunity, simple diagnostics, short infectious period, close contact required for transmission, no known significant wildlife reservoir or carrier state, and short virus survival in the environment, also apply for PPR [490,501]. Constraints to eradication include widespread distribution, high population turnover in small ruminants, low value of individual animals, and clinical disease that varies by species and breed [519]. Understanding farmer’s KAP (knowledge, attitude and practice) towards infectious diseases and consideration of gender issues are important to efforts for limiting impact and spread of disease [520,521]. Research is needed to characterize the effective reproductive number, develop thermostable and DIVA vaccines, refine targets for molecular epidemiology, increase field diagnostics, and determine the role of atypical hosts [514].

Mathematical modelling has been performed to estimate economic impact, identify risks for transmission, and evaluate possible control techniques [522,523,524,525,526]. An in-silico approach to protein analysis may help with development of vaccines and therapeutics [527]. Experimental infections of goats and sheep have been described and are suitable for comparative studies and vaccine evaluation [528,529,530]. Mice, rabbits, and horses have also played a role in diagnosis, treatment, and vaccine development [531,532,533].

### 3.13. Rift Valley Fever

Rift Valley fever virus (RVFV) causes Rift Valley fever (RVF) and is an enveloped, single stranded, negative sense (with ambisense regions), RNA virus that belongs to the *Phlebovirus* genus, in the family *Phenuiviridae* [534] (formerly of the genus *Phlebovirus*, family *Bunyaviridae* [535]). There is significant RVFV diversity with at least 33 different viruses and 15 lineages identified thus far [536]. The disease was first discovered in Kenya and is enzootic in the southern and eastern parts of Africa; it has spread across the African continent (e.g., Egypt and Sudan) and into the Arabian Peninsula (e.g., Saudi Arabia) [535,537]. Outbreaks frequently result in the death of thousands of livestock animals, with a substantial number of human infections and human deaths during some outbreaks [537].

The disease primarily impact ruminants and a main route of transmission is via mosquitoes, mainly from the *Aedes* and *Culex* genera, but the virus has been found in multiple species of mosquito from at least five additional genera, including *Anopheles*, *Coquillettidia*, *Culiseta*, *Eretmapodites, and Mansonia* [538,539,540]. A variety of other arthropod vectors, including ticks and flies, have been implicated in transmission (reviewed in [541]). Due to the role of arthropods in transmission, large outbreaks are frequently associated with periods of heavy rainfall and flooding [3]. Increasing outbreaks, or emergence in new areas, is of increasing concern due to global climate change and the large global distribution of potential vectors; the potential loss of animal or human life, and accompanying production losses, could be quite detrimental if RVF continues uncontrolled [541,542].

Direct contact with fluids or tissues from infected animals, or with contaminated fomites, pose a risk of infection for susceptible animals [541]. Sheep and cattle are the primary livestock species of concern, leading to the majority of virus spread and the majority of clinical disease [541,543,544]. However, goats, camelids, nonhuman primates, cats, dogs, and horses are also susceptible, sometimes without signs of clinical disease. Common clinical signs of RVF include abortions in female adult animals, fever, swollen lymph nodes, and lack of appetite. Weakness, nasal discharge, and bloody diarrhea are also prominent features in infected sheep [541,543,544]. Cattle tend to exhibit hypersalivation, diarrhea, and decreased milk production. Consequently, RVF can result in significant losses to production and food supply, along with high mortality, in infected livestock [3]. Young animals exhibit different disease progression and appear to more susceptible, though more research is needed to better understand the mechanisms involved in the differences between young and older animals [541,544].

Additionally, human infections are a serious concern. Humans can be exposed through contact with mosquitoes, or fetuses aborted from infected animals; consumption of animal products from infected animals or contact with various fluids such as blood, milk, or semen from infected animals; or common herdsmen activities such as slaughtering or skinning animals, or sleeping in close proximity to infected animals [545]. Humans are often asymptomatic and long term or severe sequelae are rare. In milder cases, clinical signs are generally influenza like [541]. However, in large outbreaks thousands of humans can become infected [537,541], leading to a higher number of cases of severe disease with variable clinical presentations. These manifestations can include inflammation of the liver or retina, encephalitis and neurological disease, or hemorrhagic disease, all of which are associated with increased mortality rates; the overall case fatality rate is less than 5% [541].

A variety of animal models for RVF have been developed, including ruminants such as sheep and cattle, laboratory rodents, and non-human primates (reviewed in [543]). Viremia and fever are classic signs during experimental infection. Evidence of liver involvement and liver damage (e.g., increased levels of liver enzymes) and decreased white blood cells are also seen in severe experimental infections, similar to what is observed during natural infections. Also similar to what is seen during natural infections, experimental infections can present as either severe and lethal (accompanied by very high viremia), mild or asymptomatic (accompanied by no viremia or viremia that resolves quickly), or delayed onset with more severe sequelae (accompanied by viral dissemination throughout the body) [541]. Inbred strains of laboratory rats show difference susceptibilities, which may help researchers explore the underlying causes for different susceptibilities during natural infections in target species [541].

Currently there are few fully licensed commercially available vaccines, or effective therapeutics [546,547]. In certain African countries (e.g., South Africa), a vaccine derived from a plaque purified clone of an attenuated mutant is used as a vaccine known as Clone 13; this vaccine is effective and safe but may cause problems when used in pregnant sheep [548,549]. One of the first vaccines generated to control RVF is the live attenuated Smithburn vaccine, produced from repeated passage of virus isolated from a mosquito, in mouse brain; future propagation was done in BHK-21 cells [548,549]. However, the vaccine is not fully attenuated and genetic reassortment remains a concern and thus utility is limited in certain areas. Formalin inactivated vaccines have also been produced but are either not as efficacious or requires boosters to maintain protection [548,549]. Further vaccine strategies have been developed, including vaccines designed to protect against other diseases as well [550], but also include drawbacks, thus necessitating continued work and continued vigilance [541]. Research is ongoing into effective antivirals, especially given the burden of human morbidity and mortality in large outbreaks [547].

### 3.14. Rinderpest

Rinderpest (RP), or “cattle plague,” is caused by a Morbillivirus, of the family *Paramyxoviridae* [551]. It is generally accepted that measles emergence resulted from a spillover of RP from cattle to humans, although the directionality has never been formally established [552]. Rinderpest belongs to a select group of infectious disease that have changed the course of history [553]. It is thought to have originated as far back as the domestication of cattle in Asia 10,000 years ago, probably near the Indus River [554]. Invaders from Asia likely brought Rinderpest Virus (RPV) to Europe with their Asian Grey Steppe oxen which shed virus, but were resistant to RPV effects [551]. After the establishment of RPV in Europe in the 18th century, the panzootic was eventually introduced to Africa in the 19th century [555]. The disease spread through warfare and cattle trade, and centuries of epidemics deprived people of meat, milk and the ability to till land for crops, leading to hunger and starvation [554,555]. Only one outbreak of RPV has been reported in the America’s and Australia, occurring in the 1920′s in Brazil and Australia [554].

Rinderpest virus mainly affects wild and domestic ungulates including cattle (*Bos* spp.), Asian and African buffaloes (*Bubalus bubalis* and *Syncerus caffer*), yaks (*Bos grunniens*), swine (*Sus* spp.), and giraffes (*Giraffa camelopardalis*) [551,553,556]. After a 3–6 day incubation, a prodromal phase characterized by high fever is followed by an erosive mucosal phase with severe mouth lesions and copious nasal and ocular discharges [556,557]. Eventually, a diarrheal phase is seen with severe bloody diarrhea and death from dehydration and weakness [556,557]. Three signs dominate the clinical picture: discharge, diarrhea, death [557]. From start of fever, death occurs within a week [557]. Convalescence in nonfatal cases may take weeks and include abortions, skin lesions and blindness [556]. Mortality could reach 100% in susceptible populations [554]. It was often carried unnoticed by sheep, goats and pigs and pathogenesis in wildlife was highly variable [551,557]. The Great African Pandemic of the 19th century, wiped out untold numbers of wild and domestic animals, leaving only around 5% of the previous cattle and herds of wild ungulates in sub-Saharan Africa and contributing to severe famine [554,558].

Diagnosis relies on serologic methods, but antibodies do not start developing until 2–10 days post infection [559]. Reverse transcription-PCR enables the detection of disease 2–4 days before the appearance of clinical signs [559]. No specific treatment is known [557]. Early slaughter of sick and contaminated animals was the only control option during outbreaks and effective vaccines were eventually developed [557]. There are reports from the 18th century of vaccination against cattle “distemper” by inoculating animals with cloth soaked in discharge from infected animals [560]. Starting in the late 19th century, vaccination was performed with a serum-virus active immunization technique followed by an attenuated goat tissue vaccine and lapinised RP vaccine [555]. These early vaccine strategies could induce disease and a safer attenuated vaccine was needed [555]. The Plowright tissue culture rinderpest vaccine (TCRV), developed in 1960, protected against all clades, gave lifelong immunity and had no adverse reactions, but required strict cold chain handling and could be contaminated due to use of bovine kidney cells [551]. The establishment of independent quality control and invention of a thermostable TCRV formulation contributed to vaccine campaign success [551]. Recombinant vaccines have been under development in the 21st century [555].

The consequences of RP raised attention to the studying of diseases of animals and contributed to founding of the world’s first veterinary college in France in 1761 [554,555]. By 1960, RP had been eradicated from Europe, Russia, China and the Far East, but remained entrenched in India and Africa [553]. Multiple eradication efforts with varying degrees of success were undertaken throughout the 20th century [551]. Challenges included failure to recognize circulation in wildlife and focus on vaccination without a clearly defined objective or exit plan [551]. In 1994, the FAO established the Global Rinderpest Eradication Program (GREP) and one key to success was the establishment of a 2010 deadline [551]. A 300-year global battle against RP led to declaration of worldwide eradication in 2011 [555]. Factors that helped make RPV a candidate for eradication include uncomplicated biology, a single serotype, strong immunity in recovered animals, no carrier state and no vertical or arthropod vector transmission [561]. Keys to successful eradication included a thermostable vaccine and application of participatory epidemiological techniques [562]. The collaborative effort that led to RPV eradication may be the first example of a successful “one health” approach [551].

According to a 2011 survey, 55 labs in 35 countries still held some kind of rinderpest virus [563]. The FAO and OIE aimed to reduce the number of sites holding live virus and vaccine stocks to a handful of officially designated labs [563]. Vaccination was prohibited as part of the eradication plan, meaning current cattle populations are fully susceptible and re-introduced infection would spread rapidly potentially causing “billion-dollar-scale disruption” [564,565]. The highest risk for re-introduction is probably the accidental use of laboratory virus stocks [566]. Other potential pathways include deliberate use in laboratories, use of vaccines, exposure to environmental sources, and biological warfare [566]. In 2016, the virus remained in 21 countries in 22 separate facilities, of which only five were officially inspected and approved [567]. Some labs have participated in a program called “sequence and destroy”, launched by the FAO and OIE in 2015 [564]. Full genome sequencing allows destruction of virus stocks while maintaining the ability to recover virus if needed [568]. Ironically, the advances in synthetic biology that made destruction more palatable may have raised the risk of reintroduction [569]. As of August 2019, samples were still known to be stored in China, Ethiopia, France, Japan and the United States [569].

Initial vaccine development relied on passage in goats and rabbits but was eventually transitioned to bovine kidney cells [551,555]. Rabbits and mice were used to explore various pathogenesis and immunologic topics [570,571,572,573,574]. A variety of mathematical spatial, epidemiological and risk mapping models have been developed [561,575,576,577,578,579,580]. Cattle challenge models have also been described [581]. The first approved in vivo study after the moratorium on RP research, in place from 2011–2013, investigated whether PPRV vaccines could protect against RPV outbreaks [563,581]. Rinderpest eradication itself has been used as a case study and inspiration to model and explore possible eradication of other diseases such as measles and other livestock diseases [562,582,583,584].

### 3.15. Sheeppox and Goatpox

*Sheeppox virus* (SPPV) and *Goatpox virus* (GTPV) are enveloped double-stranded DNA viruses of the genus *Capripoxvirus*, family *Poxviridae* [585]. Sheeppox (SP) and goatpox (GP) are enzootic in many African and Asian countries [586,587]. Goatpox was first reported in Norway in 1879 [585]. Sheeppox has a documented history almost as long as that of smallpox, being present as early as the second century AD [585]. Sheeppox and goatpox are generally host specific, but this may vary between isolates [585,588]. Morbidity and mortality in a susceptible flock may be 75–100% and 10–58% respectively [585]. After a 1–2 week incubation, clinical disease starts with fever, labored breathing, depression, inappetence and lymphadenopathy [585]. Skin lesions develop after 1–2 days and progress through five stages, macular, popular, vesicular, pustular and scabbing [585,589]. Animals may recover in 3–4 weeks with permanent depressed scars [585]. The generalized or malignant form of disease, characterized by prostration, high fever, depression and discharges, is most often seen in lambs and kids aged 4–5 months [585,590]. Skin, digestive, respiratory, and urogenital mucosal lesions may be seen [590]. Older animals are more likely to have mild or benign forms of disease [590]. Abortion and secondary pneumonia may also occur [585,590]. There is no effective treatment aside from providing supportive care and controlling secondary bacterial infection [585,589].

*Sheeppox virus* and *Goatpox virus* commonly enter the respiratory tract via aerosol transmission [585]. Transmission may also occur by contact with skin lesions or mechanically by insect vectors [585]. The virus is generally resistant to drying, survives freezing and thawing and remains viable for months [585]. Seasonality and environmental conditions affect spread with higher occurrence in warm and cold moist months [591,592]. The spread of disease into new areas is often associated with illegal animal movement and inadequate veterinary services [593]. Diagnosis is generally based on clinical signs and gross pathology followed by lab confirmation [585]. Histologic skin lesions are characterized by dermal edema and cellularity with variable numbers of “sheeppox cells,” histiocytic like cells with large vacuolated nuclei and poorly-defined eosinophilic cytoplasmic inclusions [594]. A variety of serologic and molecular diagnostics are available, with some molecular approaches capable of rapid detection and differentiation between GP and SP [588,595,596,597,598,599,600,601,602].

Despite decades of vaccination efforts in some areas, SP/GP still persist [603]. Previous killed vaccines lacked the extracellular virion form, resulting in poor and at best, only temporary protection, but new killed vaccines are under development [604,605]. Immunity against poxviruses is both cell mediated and humoral [606]. A variety of commercially available and locally developed live attenuated capripoxvirus vaccines have been used to provide protection against SP/GP [604,606]. Strains of capripoxviruses share a major neutralizing site giving animals recovered from infection with one strain resistance to infection with other strains [604]. It is possible to use a single strain to protect goats and sheep from all field strains, but some strains are host-specific and can only be used as a species-specific vaccination [604]. Strain variations may be associated with adaptation in the presence or either sheep or goats alone in isolated areas [560]. Confirming the identity of vaccine seed virus and clearly indicating its origin is an important part of vaccine quality control [606,607]. There is interest in creating multivalent capripoxvirus-vectored vaccines that would protect against SP/GP along with multiple other viral diseases [608,609].

With several countries already heavily reliant on small ruminants and others aiming to increase small ruminant populations in the face of the current african swine fever outbreak, the control of small ruminant diseases including SP/GP is economically crucial [603,610,611,612,613]. Early detection and notification, prompt movement restriction, and culling affected herds based on clinical signs are effective control measures [587]. Sentinel animals can be used prior to re-stocking herds [587]. Characteristics that favor the potentially successful control of SP/GP include a single serotype, no persistent infection, limited host range and available effective vaccines [593]. As with LSD, arthropod vector transmission can cause significant challenges in the control of SP/GP [372,585].

Mathematical and spatial models have been developed to better understand outbreaks, transmission and potential impact of control methods [591,611,614,615,616]. Immunogenicity and vaccination studies have been performed using both in vitro cell culture and in vivo models [585,617,618,619,620,621]. An intradermal inoculation of sheep was performed to help determine the most suitable time for collection of diagnostic specimens and provide a description of lesions [622]. Wolff et al. evaluated three different routes of SPPV infection (intravenous, intranasal and contact with infected animals) using both an Indian and Egyptian SPPV strain [623]. Intranasal inoculation of the Indian strain turned out to be the more natural and efficient challenge model for use in future vaccine studies [623]. With experimental infection showing underlying pathogenesis similar to other poxviruses, SP/GP models may be convenient for evaluation of vaccines and therapeutics, but also for the study of host-pox virus interactions [624].

### 3.16. Swine Vesicular Disease

Swine vesicular disease virus (SVDV) causes swine vesicular disease (SVD) and is a non-enveloped, single stranded, positive sense RNA virus that belongs to the genus *Enterovirus*, in the family *Picornaviridae* [625,626]. While there is genetic diversity within the genus and at least four antigenic variants, one serotype encompasses all isolates [625,627]. The virus is antigenically similar to human coxsackievirus B [626]. The disease was first reported in Italy and primarily remains a problem in Italy, but has been reported in other European and Asian countries such as Germany, Portugal, Taiwan, and China [626,628]. Pigs are the natural host of SVDV and the virus is spread through contact with other infected pigs or their bodily fluids, or with contaminated fomites [626,627]. Outside the natural host, one day old laboratory mice have been shown to be susceptible to SVDV and there is a reported case of an infected laboratory worker, who developed mild influenza like symptoms [626].

Clinical symptoms in pigs are infrequent and mild and are nearly indistinguishable from those observed in FMD infected pigs [628,629]. After infection, usually through damaged skin or mucous membranes (though ingestion is possible as well), the virus replicates to high levels before clinical signs may appear [626]. If symptoms develop, they generally consist of vesicle formation on the feet, or in/around the mouth [626]. Vesicle formation on the limbs can result in lameness, which resolves quickly as the vesicles disappear. Mortality is negligible and outbreaks with severe clinical signs are rare [626].

A primary complication in controlling SVD is the extreme stability of SVDV [626,630]. Infected pigs develop high viral titers and begin to excrete virus one to two days after infection, with shedding usually continuing for approximately one week, with shed virus then remaining present in the surrounding environment, and viral shedding may occur for months. This results in the disease being very costly to control and difficult to eradicate [626]. The virus is also more resistant to traditional or common methods of disinfection, such as detergents and organic solvents, though sodium hydroxide and formaldehyde have demonstrated efficacy [629]. Another challenge is related to mild or asymptomatic presentation. Since disease is generally mild, food products from infected animals are more likely to enter the food chain [626,630]. Infected animals are difficult to recognize, and the disease often spreads to numerous animals, or nearby farms, before SVDV is recognized. As outbreaks of SVD are controlled by slaughter and livestock trade restrictions, SVD can cause high economic loss despite the low morbidity and mortality [626]. The similarity to FMD is largely responsible for why the control of SVD is so important, due to the implications for trade [631]. Areas currently free of SVDV are at risk for importing the virus in livestock or food products, further leading to a need for increased control and surveillance [631].

Control efforts are further hampered by challenges in diagnostics. Due to the similarity to other vesicular diseases, laboratory-based diagnostics are crucial for control and identification. However, commonly used serological tests can produce false negatives and researchers are working toward developing more reliable tests [632,633]. The majority of laboratory research is aimed at diagnostics [632] and understanding environmental persistence [630]. In vitro cell based assays, aimed at understanding basic virus biology such as cell entry and spread [634,635,636], are more common than animal modeling. As pigs are the only natural host, experimental modeling studies focus on using pigs and are generally targeted at understanding persistence or shedding, and vaccine testing. While modeling studies have been underway for decades, there is little consistency in the elements of the model (e.g., route of exposure, dose, strain, or experimental design [637,638,639,640,641]).

### 3.17. Vesicular Stomatitis

Vesicular stomatitis viruses (VSV) cause vesicular stomatitis (VS) and are enveloped, single stranded, negative sense RNA viruses belonging to the genus *Vesiculovirus*, in the family *Rhabdoviridae* [628,642,643]. The viruses are transmitted to a wide variety of hosts via arthropods, such as flies, mosquitoes, and midges [628,642,643]. Infection with VSV was likely described in the USA in the 1800s, based on symptoms in sick horses, cattle, and pigs [642], but is generally accepted as being first identified in 1916 in the USA during an epidemic and cattle and horses [628,642,643]. The virus is enzootic in Mexico, Central America, and Northern South America—with high rates of seropositivity in humans in countries such as Panama—and widely distributed across the Americas [628,642,643,644]. There are dozens of recognized species within the genus, but the more well characterized and more significant New World species include VSV-New Jersey (VSV-NJ), VSV-Indiana (VSV-IN), Alagoas, Chalchaqui, Cocal, and Piry [642].

The virus can infect livestock through aerosol exposure, via arthropod, or from contact with infected animals or contaminated fomites. Infections can be asymptomatic, especially in younger animals [645], but disease can also be acute. Acute disease symptoms present in domesticated animals such as cattle, horses, and pigs. Symptoms are similar to FMDV, including fever, swelling of the mouth or nose, lameness, depression, hypersalivation, vesicular lesions in or around the mouth, on the feet, or the teats [628,642,643], though lesions may be less prevalent in horses. Sheep and goats can be infected experimentally but natural infection is rare [628,642,643] and there is some evidence of infection in a wider range of animals such as wild ruminants and carnivorous mammals, other hooved wildlife, or rodents [628,642,643]. Human infections are usually mild or symptomatic [628,642,643]. However, symptoms beyond influenza like symptoms have been reported, including conjunctivitis, nausea and vomiting, lymphadenitis, muscle pain, lesions in the mouth or skin, or rarely encephalitis (reviewed in [642]).

VSV is a prototypical virus for molecular biology and virology studies, especially in the field of viral diversity and evolution [628,642,643]. Numerous in vitro techniques exist to study the virus; VSV replicates in a large number of immortalized cell lines, such as BHK-21 cells, as well as immortalized or primary cells derived from insects, birds, mammals, reptiles, and fish [628,642,643]. Experimental infections have been performed in a large, diverse variety of animals over the past century (reviewed in [646]). In larger animal models, direct injection of the virus is frequently used and results in similar symptoms to natural infections. Under laboratory conditions, cattle often develop lesions near the site of virus injection, though the lesions last only a few days; direct inoculation of the tongue has been shown to cause fever, hypersalivation, lack of appetite, and the expected vesicular lesions [642]. Small animal models, primarily mice, are commonly used to study pathogenesis and immune responses [647,648,649]. Other small animal models, such as guinea pigs, hamsters, ferrets, and chickens, have also been developed [642]. Unsurprisingly, the outcome of experimental infection is largely depending on the dose administered, the route of inoculation, and the species or strain of virus used [647].

Outbreaks in livestock can result in substantial economic losses due to loss in animal productivity, considerable weight loss in infected animals, or lack of milk production (in dairy cattle) [642,643,650]. There is also a significant economic impact from diagnosis and control efforts. As the disease presents similarly to FMD, the identification of symptomatic animals requires immediate diagnostic testing—often through RT-PCR or ELISA—and positive test results usually trigger quarantine. Insect control measures can be challenging to implement, but are another important control measure, along with the disinfection of contaminated surfaces [628,642,643]. Vaccination is another control measure to be considered in enzootic countries. Research in mice has demonstrated neutralizing antibodies as a result of infection [649], and cattle have been shown to develop antibodies. However, the significance and implications for protection against natural infection is unclear. Thus, research into vaccines is crucial and ongoing but much remains unknown about their efficacy and duration of protection. Vaccines using wild type virus have been used but should only be considered in emergent situations as the consequences can be undesirable. As such, vaccines using inactivated virus have been implemented but may be less efficacious [642]. For other viral diseases, vector based vaccines that rely on a VSV backbone are being increasingly developed and evaluated (reviewed in [651]), though more information regarding the safety and implications of such vaccines is required [642,651]. Further work is also needed to develop better vaccination strategies, and to better understand the natural life cycle of the virus [642].

## 4. Discussion

Transboundary animal diseases are highly contagious or transmissible, epidemic diseases, with the potential to spread rapidly across the globe, cause substantial socioeconomic losses, and result in negative public health outcomes [1,2]. These diseases can threaten the global food supply, by reducing the availability of animal products, causing significant socioeconomic consequences, having severe public health consequences, and causing pain or suffering in afflicted animals.

The potential economic impacts from TADs are quite severe. Defining these costs is crucial to securing government and public commitment to disease control programs [512]. During the Great African Pandemic of the 1890s, Africa lost over 2.5 million cattle to RP [556]. In addition to economic impacts, the potential for human suffering and loss of life due to TADs is staggering. The loss of most of Ethiopia’s cattle to RP in the late 19th century contributed directly to famine that claimed the lives of up to one-third of the country’s human population [556]. After the eradication of RP was declared in 2011, analysis of costs and benefits were performed to demonstrate the positive impact of eradication efforts. For example, the benefit of the Pan-African Rinderpest Campaign, which had both regional and international support, was estimated to be up to 35,433,000 European currency units (ECU or XEU, later replaced by the Euro; approximately $43,000,000) [554]. In contrast to a disease with such obviously devastating impacts, it can be challenging to demonstrate the overall importance of eradicating diseases with low mortality or that primarily affect animals of lower individual value or that are relied on mainly by individuals in poverty-stricken regions [519,612]. Approaches that include participatory epidemiology and studies focusing on the impact on small stakeholders could help address these issues [3,271,652,653].

Further research is clearly needed for better diagnostics, especially in instances where diseases resemble one another (e.g., FMD, VSV, SVD). In addition to a requirement for very effective diagnostics (e.g., low instance of false negative or false positives), diagnostics must be widely available and economically obtainable to be impactful. As with diagnostics, more work is desperately needed for vaccines to prevent TADs. While some of these diseases have effective vaccines, most do not or the vaccines available are not as effective as they could be. Control efforts are expensive, on top of the economic impact caused by the diseases themselves, and communities are understandably reluctant to invest time or money into vaccines that do not stop spread or that cause side effects. Mistrust from previous vaccines that have showed low efficacy or side effects can also cause communities to hesitate before implementing new vaccines. Proper prevention or containment of some TADs may lessen the severity of others as well. For example, some diseases such as SVV or VSV have lower incidence and result in less severe disease, but their resemblance to FMD require their rapid identification and control. If FMD control measures succeed, SVV and VSV control may be easier as a result. In contrast, successful efforts to control one disease could inadvertently contribute to the emergence of another. For example, eradication of RP may have facilitated spread of PPR because goats and sheep exposed to RPV were immune to PPRV [493]. Lessons learned from eradicated diseases like RP and smallpox can be applied to successful control of related disease in animals and humans [511,654].

In order to develop appropriate diagnostics and vaccines, and better understand the pathogenesis of TADs, there is a need for more and better characterized animal models. Well characterized animal models must demonstrate that disease in the model is well understood and be used to adequately show efficacy and efficacy endpoints that are related to outcomes in the natural host (e.g., increased survival). Developing such models relies on characterizing a specific agent (e.g., species or strain), a relevant route and target dose, and appropriate disease outcomes that recapitulate natural disease [291,655,656]. In many cases for TADs, research design varies between facilities and standard models have not been developed.

Many of the causative agents described herein, especially the RNA viruses, have high mutation rates which result in heterogenous populations (quasispecies) (reviewed in [657]). This high level of diversity and ability to mutate quickly has many implications for TADs. For example, these viruses are more likely to be able to escape immune pressures, including vaccine induced immune responses, and therapeutics. High genetic diversity also makes developing an effective vaccine against all existing variants more difficult. These topics are especially important for TADs, as vaccine control is crucial in many cases. As the viruses mutate, they may also exhibit modified virulence, disease symptoms, tropism, or host range. As a result, emergence or re-emergence of these diseases can be impacted by their propensity for genetic diversity as mutants may spread to new geographic regions, new hosts, or via new modes of transmission. Mutation rate and quasispecies should be considered an important research area for TADs.

## 5. Conclusions

Transboundary animal diseases remain a serious threat to the global food supply and have significant socioeconomic consequences, along with a potential for severe public health consequences. This will continue until we have a better understanding of the diseases and their causative agents, better control measures available, and better strategies for implementing control measures that rely on community buy in at the local level.

## Figures and Tables

**Table 1:** Transboundary animal diseases overview.

Disease	Causative Agent	Species Affected	Symptoms	Other
African horse sickness	African horse sickness virus (*Orbivirus*)	Equids; primarily horses	Horses—acute (pulmonary) and chronic (cardiac) with high morbidity and mortality, mules and donkeys—mild disease, zebras—usually asymptomatic	
African swine fever	African swine fever virus (*Asfivirus*)	Domestic and wild suids	Sudden death, shock, hemorrhagic fever, pulmonary edema, depression, anorexia, thrombocytopenia, lymphopenia	
Avian influenza	Avian influenza virus (*Influenza A*)	Domestic poultry; birds and mammals	Highly pathogenic avian influenza (H5 and H7) causes high rates of mortality, respiratory symptoms, sinus or head swelling, depression, anorexia, cyanosis, incoordination, neurologic symptoms, diarrhea	Zoonotic
Bluetongue	Bluetongue virus (*Orbivirus*)	Domestic and wild ruminants; primarily sheep	Fever, swelling, vascular injury and hemorrhage, ulceration, pulmonary edema, muscle necrosis; or asymptomatic	
Classical swine fever	Classical swine fever virus (*Pestivirus*)	Domestic and wild suids	Acute, chronic, and prenatal presentations; sudden death, hemorrhagic fever, stillbirth, abortion, mummification, malformations, persistent infection, congenital tremor	
Contagious bovine pleuropneumonia	*Mycoplasma mycoides* subsp. *Mycoides*	Domestic and wild large ruminants; primarily cattle	Fever, inappetence, depression, labored breathing, coughing, nasal discharge, salivation (may vary from sudden death to chronic subclinical carrier)	
Foot and mouth disease	Foot and mouth disease virus (*Aphthovirus*)	Cloven-hooved animals	Fever, vesicles on the feet, tongue, snout, muzzle, mammary glands, genital mucosa, or other mucosal sites, inappetence, lameness	
Hemorrhagic septicemia	*Pasteurella multocida*	Cattle and buffalo	Fever, submandibular and brisket edema, respiratory distress and mucopurulent or bloody nasal discharge (may vary from sudden death to chronic subclinical carrier)	
Lumpy skin disease	Lumpy skin disease virus (*Capripoxvirus*)	Cattle and buffalo	Lacrimation, nasal discharge, inappetence, lymphadenopathy, fever, drop in milk production, lameness, nodular skin lesions, sometimes death	
Middle East respiratory syndrome	Middle East respiratory syndrome coronavirus (*Betacoronavirus*)	Camels	Mild upper respiratory disease	Zoonotic
Newcastle disease	Newcastle disease virus (*Avulavirus*)	Primarily domestic chickens; reptiles, birds, and mammals possible	Five pathotypes (viscerotropic velogenic, neurotropic velogenic, mesogenic, lentogenic, and asymptomatic enteric); neurologic and respiratory disease; asymptomatic	Zoonotic
Peste des petits ruminants	Peste des petits ruminants virus (*Morbillivirus*)	Domestic and wild ruminants; primarily goats and sheep	Inappetence, emaciation, depression, fever, diarrhea, nasal and ocular discharge, pneumonia and erosive and necrotic stomatitis, death	Targeted for eradication by 2030
Rift Valley fever	Rift Valley fever virus (*Phlebovirus*)	Ruminants	Abortion, fever, lymphadenopathy and inappetence; weakness, nasal discharge, and bloody diarrhea (sheep); hypersalivation, diarrhea, and decreased milk production (cattle)	Zoonotic
Rinderpest	Rinderpest virus (*Morbillivirus*)	Domestic and wild ungulates	Fever, erosive mouth lesions, nasal and ocular discharge, bloody diarrhea, dehydration, weakness, death	Eradicated, 2011
Sheeppox and goatpox	*Sheeppox virus* and *Goatpox virus* (*Capripoxvirus*)	Sheep and goats	Fever, labored breathing, depression, inappetence and lymphadenopathy, progressive skin lesions: macular, popular, vesicular, pustular and scabbing; sometimes death	
Swine vesicular disease	Swine vesicular disease virus (*Enterovirus*)	Suids	Infrequent and mild; influenza-like	
Vesicular stomatitis	Vesicular stomatitis virus (*Vesiculovirus*)	Horses, cattle and suids; rarely sheep and goats	Vesicular lesions on feet, snout, lips, and tongue	

## Data Availability

Not applicable.

## References

[1] World Organization for Animal Health (2021). Animal Diseases.

[2] Food and Agriculture Organization of the United Nations (2021). Transboundary Animal Diseases. www.fao.org/emergencies/emergency-types/transboundary-animal-diseases/en/.

[3] Calkins C.M., Scasta J.D. (2020). Transboundary Animal Diseases (Tads) Affecting Domestic and Wild African Ungulates: African Swine Fever, Foot and Mouth Disease, Rift Valley Fever (1996–2018). Res. Vet. Sci..

[4] Rossiter P.B., al Hammadi N. (2008). Living with Transboundary Animal Diseases (TADs). Trop. Anim. Health Prod..

[5] Torres-Velez F., Havas K.A., Spiegel K., Brown C. (2019). Transboundary Animal Diseases as Re-Emerging Threats—Impact on One Health. Semin. Diagn. Pathol..

[6] Carpenter S., Mellor P.S., Fall A.G., Garros C., Venter G.J. (2017). African Horse Sickness Virus: History, Transmission, and Current Status. Annu. Rev. Entomol..

[7] Mirchamsy H., Hazrati A. (1973). A Review on Aetiology and Pathogeny of African Horsesickness. Arch. Razi. Inst..

[8] Roy P., Mertens P.P., Casal I. (1994). African Horse Sickness Virus Structure. Comp. Immunol. Microbiol. Infect. Dis..

[9] Wood H.A. (1973). Viruses with Double-Stranded RNA Genomes. J. Gen. Virol..

[10] Dennis S.J., Meyers A.E., Hitzeroth I., Rybicki E.P. (2019). African Horse Sickness: A Review of Current Understanding and Vaccine Development. Viruses.

[11] Wall G.V., Wright I.M., Barnardo C., Erasmus B.J., van Staden V., Potgieter A.C. (2021). African Horse Sickness Virus NS4 Protein Is an Important Virulence Factor and Interferes with JAK-STAT Signaling during Viral Infection. Virus Res..

[12] Zientara S., Weyer C.T., Lecollinet S. (2015). African Horse Sickness. Rev. Sci. Tech..

[13] Zwart L., Potgieter C.A., Clift S.J., van Staden V. (2015). Characterising Non-Structural Protein NS4 of African Horse Sickness Virus. PLoS ONE.

[14] Ngoveni H.G., van Schalkwyk A., Koekemoer J.J.O. (2019). Evidence of Intragenic Recombination in African Horse Sickness Virus. Viruses.

[15] Boinas F., Calistrib P., Domingo M., Avilés M.M., López B.M., Sánchez B.R., Sánchez-Vizcaíno J.M. (2009). Scientific Report Submitted to EFSA: Scientific Review on African Horse Sickness. EFSA Support. Publ..

[16] Gilkerson J.R., Bailey K.E., Diaz-Méndez A., Hartley C.A. (2015). Update on Viral Diseases of the Equine Respiratory Tract. Vet. Clin. N. Am. Equine Pract..

[17] Mellor P.S., Hamblin C. (2004). African Horse Sickness. Vet. Res..

[18] Van Vuuren M., Penzhorn B.L. (2015). Geographic Range of Vector-Borne Infections and Their Vectors: The Role of African Wildlife. Rev. Sci. Tech..

[19] Robin M., Page P., Archer D., Baylis M. (2016). African Horse Sickness: The Potential for an Outbreak in Disease-Free Regions and Current Disease Control and Elimination Techniques. Equine Vet. J..

[20] Becker E., Venter G.J., Greyling T., Molini U., van Hamburg H. (2018). Evidence of African Horse Sickness Virus Infection of Equus Zebra Hartmannae in the South-Western Khomas Region, Namibia. Transbound. Emerg. Dis..

[21] Porphyre T., Grewar J.D. (2019). Assessing the Potential of Plains Zebra to Maintain African Horse Sickness in the Western Cape Province, South Africa. PLoS ONE.

[22] Castillo-Olivares J. (2021). African Horse Sickness in Thailand: Challenges of Controlling an Outbreak by Vaccination. Equine. Vet. J..

[23] King S., Rajko-Nenow P., Ashby M., Frost L., Carpenter S., Batten C. (2020). Outbreak of African Horse Sickness in Thailand, 2020. Transbound. Emerg. Dis..

[24] Lu G., Pan J., Ou J., Shao R., Hu X., Wang C., Li S. (2020). African Horse Sickness: Its Emergence in Thailand and Potential Threat to Other Asian Countries. Transbound. Emerg. Dis..

[25] Mellor P.S., Boned J., Hamblin C., Graham S. (1990). Isolations of African Horse Sickness Virus from Vector Insects Made during the 1988 Epizootic in Spain. Epidemiol. Infect..

[26] Rodriguez M., Hooghuis H., Castaño M. (1992). African Horse Sickness in Spain. Vet. Microbiol..

[27] Thompson G.M., Jess S., Murchie A.K. (2012). A Review of African Horse Sickness and Its Implications for Ireland. Ir. Vet. J..

[28] Guichard S., Guis H., Tran A., Garros C., Balenghien T., Kriticos D.J. (2014). Worldwide niche and future potential distribution of Culicoides imicola, a major vector of bluetongue and African horse sickness viruses. PLoS ONE.

[29] Martinez-de la Puente J., Navarro J., Ferraguti M., Soriguer R., Figuerola J. (2017). First molecular identification of the vertebrate hosts of Culicoides imicola in Europe and a review of its blood-feeding patterns worldwide: Implications for the transmission of bluetongue disease and African horse sickness. Med. Vet. Entomol..

[30] Nielsen S.S., Alvarez J., Bicout D.J., Calistri P., Depner K., Drewe J.A., Garin-Bastuji B., Rojas J.L.G., Schmidt C.G., Herskin M. (2021). Scientific Opinion on the Assessment of the Control Measures of the Category A Diseases of Animal Health Law: African Horse Sickness. EFSA J..

[31] Toh X., Wang Y., Rajapakse M.P., Lee B., Songkasupa T., Suwankitwat N., Kamlangdee A., Fernandez C.J., Huangfu T. (2021). Use of Nanopore Sequencing to Characterize African Horse Sickness Virus (AHSV) from the African Horse Sickness Outbreak in Thailand in 2020. Transbound. Emerg. Dis..

[32] Faber E., Tshilwane S.I., Kleef M.V., Pretorius A. (2021). Virulent African Horse Sickness Virus Serotype 4 Interferes with the Innate Immune Response in Horse Peripheral Blood Mononuclear Cells In Vitro. Infect. Genet. Evol..

[33] OIE (2019). Application for the official recognition by the OIE of free status for African horse sickness. Terrestrial Animal Health Code.

[34] Redmond E.F., Jones D., Rushton J.J.E.V.J. (2021). Economic Assessment of African Horse Sickness Vaccine Impact. Equine Vet. J..

[35] Weyer C.T., Grewar J.D., Burger P., Rossouw E., Lourens C., Joone C., le Grange M., Coetzee P., Venter E., Martin D.P. (2016). African Horse Sickness Caused by Genome Reassortment and Reversion to Virulence of Live, Attenuated Vaccine Viruses, South. Africa, 2004–2014. Emerg. Infect. Dis..

[36] Wernery U., Joseph S., Raghavan R., Dyer B., Spendrup S. (2020). African Horse Sickness Fever in Vaccinated Horses: Short Communication. J. Equine Vet. Sci..

[37] Grewar J.D., Sergeant E.S., Weyer C.T., van Helden L.S., Parker B.J., Anthony T., Thompson P.N. (2019). Establishing Post-Outbreak Freedom from African Horse Sickness Virus in South Africa’s Surveillance Zone. Transbound. Emerg. Dis..

[38] Wernery U., Rodriguez M., Raghavan R., Syriac G., Miriam Thomas S., Elizabeth S.K., Federico Ronchi G., Muhammed R., Patteril N.A., Joseph S. (2021). Humoral Antibody Response of 10 Horses after Vaccination against African Horse Sickness with an Inactivated Vaccine Containing All 9 Serotypes in One Injection. Equine. Vet. J..

[39] Lelli R., Molini U., Ronchi G.F., Rossi E., Franchi P., Ulisse S., Armillotta G., Capista S., Khaiseb S., di Ventura M. (2013). Inactivated and Adjuvanted Vaccine for the Control of the African Horse Sickness Virus Serotype 9 Infection: Evaluation of Efficacy in Horses and Guinea-Pig Model. Vet. Ital..

[40] Dennis S.J., O’Kennedy M.M., Rutkowska D., Tsekoa T., Lourens C.W., Hitzeroth I., Meyers A.E., Rybicki E.P. (2018). Safety and Immunogenicity of Plant-Produced African Horse Sickness Virus-Like Particles in Horses. Vet. Res..

[41] Rodríguez M., Joseph S., Pfeffer M., Raghavan R., Wernery U. (2020). Immune Response of Horses to Inactivated African Horse Sickness Vaccines. BMC Vet. Res..

[42] Calvo-Pinilla E., Gubbins S., Mertens P., Ortego J., Castillo-Olivares J. (2018). The Immunogenicity of Recombinant Vaccines Based on Modified Vaccinia Ankara (MVA) Viruses Expressing African Horse Sickness Virus VP2 Antigens Depends on the Levels of Expressed VP2 Protein Delivered to the Host. Antivir. Res..

[43] Calvo-Pinilla E., Marín-López A., Utrilla-Trigo S., Jiménez-Cabello L., Ortego J. (2020). Reverse Genetics Approaches: A Novel Strategy for African Horse Sickness Virus Vaccine Design. Curr. Opin. Virol..

[44] O’Hara R., Meyer A., Burroughs J., Pullen L., Martin L.-A., Mertens P. (1998). Development of a Mouse Model System, Coding Assignments and Identification of the Genome Segments Controlling Virulence of African Horse Sickness Virus Serotypes 3 and 8. African Horse Sickness.

[45] Aksular M., Calvo-Pinilla E., Marín-López A., Ortego J., Chambers A.C., King L.A., Castillo-Olivares J. (2018). A Single Dose of African Horse Sickness Virus (AHSV) VP2 Based Vaccines Provides Complete Clinical Protection in a Mouse Model. Vaccine.

[46] Castillo-Olivares J., Calvo-Pinilla E., Casanova I., Bachanek-Bankowska K., Chiam R., Maan S., Nieto J.M., Ortego J., Mertens P.P. (2011). A Modified Vaccinia Ankara Virus (MVA) Vaccine Expressing African Horse Sickness Virus (AHSV) VP2 Protects against AHSV Challenge in an IFNAR −/− Mouse Model. PLoS ONE.

[47] Jones L., Hawes P., Salguero J., Castillo-Olivares J.J.A.M. (2019). African Horse Sickness Virus: Pathogenicity in an IFNAR (−/−) Mouse Model of Infection. Access Microbiol..

[48] Ronchi G.F., Ulisse S., Rossi E., Franchi P., Armillotta G., Capista S., Peccio A., di Ventura M., Pini A. (2012). Immunogenicity of Two Adjuvant Formulations of an Inactivated African Horse Sickness Vaccine in Guinea-Pigs and Target Animals. Vet. Ital..

[49] Salas M.L., Andrés G.J.V.R. (2013). African Swine Fever Virus Morphogenesis. Virus Res..

[50] Liu S., Luo Y., Wang Y., Li S., Zhao Z., Bi Y., Sun J., Peng R., Song H., Zhu D. (2019). Cryo-EM Structure of the African Swine Fever Virus. Cell Host Microbe.

[51] Galindo I., Alonso C.J.V. (2017). African Swine Fever Virus: A Review. Viruses.

[52] Dixon L.K., Stahl K., Jori F., Vial L., Pfeiffer D.U. (2020). African Swine Fever Epidemiology and Control. Annu. Rev. Anim. Biosci..

[53] Karger A., Pérez-Núñez D., Urquiza J., Hinojar P., Alonso C., Freitas F.B., Revilla Y., le Potier M.-F., Montoya M.J.V. (2019). An Update on African Swine Fever Virology. Viruses.

[54] Alejo A., Matamoros T., Guerra M., Andrés G.J.J.O.V. (2018). A Proteomic Atlas of the African Swine Fever Virus Particle. J. Virol..

[55] Sánchez-Vizcaíno J.M., Martínez-López B., Martínez-Avilés M., Martins C., Boinas F., Vialc L., Michaud V., Jori F., Etter E., Albina E. (2009). Scientific Review on African Swine Fever. EFSA Support. Publ..

[56] Arias M., Jurado C., Gallardo C., Fernández-Pinero J., Sánchez J.J.T., Vizcaíno M.S. (2018). Gaps in African Swine Fever: Analysis and Priorities. Transbound. Emerg. Dis..

[57] Costard S., Wieland B., de Glanville W., Jori F., Rowlands R., Vosloo W., Roger F., Pfeiffer D.U., Dixon L.K. (2009). African Swine Fever: How Can Global Spread Be Prevented?. Philos. Trans. R. Soc. B.

[58] Patil S.S., Suresh K.P., Vashist V., Prajapati A., Pattnaik B., Roy P.J.V.W. (2020). African Swine Fever: A Permanent Threat to Indian Pigs. Vet. World.

[59] Gao X., Liu T., Liu Y., Xiao J., Wang H. (2021). Transmission of African Swine Fever in China through Legal Trade of Live Pigs. Transbound. Emerg. Dis..

[60] Ma J., Chen H., Gao X., Xiao J., Wang H. (2020). African Swine Fever Emerging in China: Distribution Characteristics and High-Risk Areas. Prev. Vet. Med..

[61] Zhou X., Li N., Luo Y., Liu Y., Miao F., Chen T., Zhang S., Cao P., Li X., Tian K. (2018). Emergence of African Swine Fever in China, 2018. Transbound. Emerg. Dis..

[62] Wang N., Zhao D., Wang J., Zhang Y., Wang M., Gao Y., Li F., Wang J., Bu Z., Rao Z. (2019). Architecture of African Swine Fever Virus and Implications for Viral Assembly. Science.

[63] Blome S., Franzke K., Beer M. (2020). African Swine Fever—A Review of Current Knowledge. Virus Res..

[64] Wu K., Liu J., Wang L., Fan S., Li Z., Li Y., Yi L., Ding H., Zhao M., Chen J. (2020). Current State of Global African Swine Fever Vaccine Development under the Prevalence and Transmission of ASF in China. Vaccines..

[65] OIE (2019). African swine fever (infection with african swince fever). Terrestrial Animal Health Manual.

[66] Penrith M.-L., Vosloo W. (2009). Review of African Swine Fever: Transmission, Spread and Control. J. S. Afr. Vet. Assoc..

[67] Sánchez-Cordón P., Montoya M., Reis A., Dixon L. (2018). African Swine Fever: A Re-Emerging Viral Disease Threatening the Global Pig Industry. Vet. J..

[68] Hervás J., Gómez-Villamandos J., Méndez A., Carrasco L., Sierra M. (1996). The Lesional Changes amd Pathogenesis in the Kidney in African Swine Fever. Vet. Res. Commun..

[69] Revilla Y., Perez-Nunez D., Richt J.A. (2018). African Swine Fever Virus Biology and Vaccine Approaches. Adv. Virus Res..

[70] Gaudreault N.N., Madden D.W., Wilson W.C., Trujillo J.D., Richt J.A. (2020). African Swine Fever Virus: An. Emerging DNA Arbovirus. Front. Vet. Sci..

[71] Gaudreault N.N., Richt J.A. (2019). Subunit Vaccine Approaches for African Swine Fever Virus. Vaccines.

[72] Dixon L.K., DChapman A., Netherton C.L., Upton C. (2013). African Swine Fever Virus Replication and Genomics. Virus Res..

[73] Dixon L., Islam M., Nash R., Reis A. (2019). African Swine Fever Virus Evasion of Host Defences. Virus Res..

[74] Razzuoli E., Franzoni G., Carta T., Zinellu S., Amadori M., Modesto P., Oggiano A. (2020). Modulation of Type I Interferon System by African Swine Fever Virus. Pathogens.

[75] Zhuo Y., Guo Z., Ba T., Zhang C., He L., Zeng C., Dai H. (2021). African Swine Fever Virus MGF360–12L Inhibits Type I Interferon Production by Blocking the Interaction of Importin A and NF-Κb Signaling Pathway. Virol. Sin..

[76] Sánchez E.G., Pérez-Núñez D., Revilla Y. (2019). Development of Vaccines against African Swine Fever Virus. Virus Res..

[77] Teklue T., Sun Y., Abid M., Luo Y., Qiu H.J. (2020). Current Status and Evolving Approaches to African Swine Fever Vaccine Development. Transbound. Emerg. Dis..

[78] Bosch-Camós L., López E., Rodriguez F. (2020). African Swine Fever Vaccines: A Promising Work Still in Progress. Porc. Health Manag..

[79] Sang H., Miller G., Lokhandwala S., Sangewar N., Waghela S.D., Bishop R.P., Mwangi W. (2020). Progress toward Development of Effective and Safe African Swine Fever Virus Vaccines. Front. Vet. Sci..

[80] Rock D. (2017). Challenges for African Swine Fever Vaccine Development—“…Perhaps the End of the Beginning”. Vet. Microbiol..

[81] Carlson J., O’Donnell V., Alfano M., Salinas L.V., Holinka L.G., Krug P.W., Gladue D.P., Higgs S., Borca M.V. (2016). Association of the Host Immune Response with Protection Using a Live Attenuated African Swine Fever Virus Model. Viruses.

[82] Neilan J.G., Zsak L., Lu Z., Burrage T.G., Kutish G.F., Rock D.L. (2004). Neutralizing Antibodies to African Swine Fever Virus Proteins P30, P54, and P72 Are Not Sufficient for Antibody-Mediated Protection. Virology.

[83] Schulz K., Staubach C., Blome S. (2017). African and Classical Swine Fever: Similarities, Differences and Epidemiological Consequences. Vet. Res..

[84] Yoo D., Kim H., Lee J.Y., Yoo H.S. (2020). African Swine Fever: Etiology, Epidemiological Status in Korea, and Perspective on Control. J. Vet. Sci..

[85] Dixon L., Sun H., Roberts H. (2019). African Swine Fever. Antivir. Res..

[86] Arias M., Sánchez-Vizcaíno J., Morilla A., Yoon K., Zimmerman J. (2002). Trends in Emerging Viral Infections of Swine.

[87] Mur L., Atzeni M., Martínez-López B., Feliziani F., Rolesu S., Sanchez-Vizcaino J. (2016). Thirty-Five-Year Presence of African Swine Fever in Sardinia: History, Evolution and Risk Factors for Disease Maintenance. Transbound Emerg. Dis..

[88] Mur L., Sánchez-Vizcaíno J., Fernández-Carrión E., Jurado C., Rolesu S., Feliziani F., Laddomada A., Martínez-López B. (2018). Understanding African Swine Fever Infection Dynamics in Sardinia Using a Spatially Explicit Transmission Model in Domestic Pig Farms. Transbound Emerg. Dis..

[89] Swayne D.E. (2009). Avian Influenza.

[90] Swayne D.E. (2007). Understanding the Complex Pathobiology of High Pathogenicity Avian Influenza Viruses in Birds. Avian. Dis..

[91] Alexander D.J. (2000). A Review of Avian Influenza in Different Bird Species. Vet. Microbiol..

[92] Lee D.H., Bertran K., Kwon J.H., Swayne D.E. (2017). Evolution, Global Spread, and Pathogenicity of Highly Pathogenic Avian Influenza H5Nx Clade 2.3.4.4. J. Vet. Sci..

[93] Lee D.H., Criado M.F., Swayne D.E. (2021). Pathobiological Origins and Evolutionary History of Highly Pathogenic Avian Influenza Viruses. Cold Spring Harb. Perspect. Med..

[94] Peacock T.H.P., James J., Sealy J.E., Iqbal M. (2019). A Global Perspective on H9N2 Avian Influenza Virus. Viruses.

[95] Spickler A.R. (2016). Highly Pathogenic Avian Influenza.

[96] Shriner S.A., Root J.J. (2020). A Review of Avian Influenza A Virus Associations in Synanthropic Birds. Viruses.

[97] Root J., Shriner S. (2020). Avian Influenza A Virus Associations in Wild, Terrestrial Mammals: A Review of Potential Synanthropic Vectors to Poultry Facilities. Viruses.

[98] Gorsich E.E., Webb C.T., Merton A.A., Hoeting J.A., Miller R.S., Farnsworth M.L., Swafford S.R., DeLiberto T.J., Pedersen K., Franklin A.B. (2021). Continental-Scale Dynamics of Avian Influenza in U.S. Waterfowl Are Driven by Demography, Migration, and Temperature. Ecol. Appl..

[99] Khan M., Chaudhry M., Fatima Z., Khan R.U., Ahmad B., Ullah R., Khan A. (2021). Effect of Avian Influenza H9N2 Subtype Virus Infection on Backyard Poultry Production. Sci. Lett..

[100] Gu M., Xu L., Wang X., Liu X. (2017). Current Situation of H9N2 Subtype Avian Influenza in China. Vet. Res..

[101] Kim Y., Biswas P.K., Giasuddin M., Hasan M., Mahmud R., Chang Y.M., Essen S., Samad M.A., Lewis N.S., Brown I.H. (2018). Prevalence of Avian Influenza A(H5) and A(H9) Viruses in Live Bird Markets, Bangladesh. Emerg. Infect. Dis..

[102] Bi Y., Chen Q., Wang Q., Chen J., Jin T., Wong G., Quan C., Liu J., Wu J., Yin R. (2016). Genesis, Evolution and Prevalence of H5N6 Avian Influenza Viruses in China. Cell Host Microb..

[103] Tian H., Zhou S., Dong L., van Boeckel T.P., Cui Y., Newman S.H., Takekawa J.Y., Prosser D.J., Xiao X., Wu Y. (2015). Avian Influenza H5N1 Viral and Bird Migration Networks in Asia. Proc. Natl. Acad. Sci. USA.

[104] Nuñez I.A., Ross T.M. (2019). A Review of H5Nx Avian Influenza Viruses. Adv. Vaccines Immunother..

[105] OIE (2021). Highly Pathogenic Avian Influenza (HPAI) Report N° 21: January 15 to February 04, 2021.

[106] Verhagen J.H., Fouchier R.A.M., Lewis N. (2021). Highly Pathogenic Avian Influenza Viruses at the Wild-Domestic Bird Interface in Europe: Future Directions for Research and Surveillance. Viruses.

[107] Suarez D.L., Das A., Ellis E. (2007). Review of Rapid Molecular Diagnostic Tools for Avian Influenza Virus. Avian Dis..

[108] Beerens N., Koch G., Heutink R., Harders F., Vries D.E., Ho C., Bossers A., Elbers A. (2018). Novel Highly Pathogenic Avian Influenza A (H5N6) Virus in the Netherlands, December 2017. Emerg. Infect. Dis..

[109] OIE (2018). Terrestrial Animal Health Manual.

[110] Liu S., Zhuang Q., Wang S., Jiang W., Jin J., Peng C., Hou G., Li J., Yu J., Yu X. (2020). Control. of Avian Influenza in China: Strategies and Lessons. Transbound. Emerg. Dis..

[111] Gilbert M., Golding N., Zhou H., Wint G.W., Robinson T.P., Tatem A.J., Lai S., Zhou S., Jiang H., Guo D. (2014). Predicting the Risk of Avian Influenza a H7N9 Infection in Live-Poultry Markets across Asia. Nat. Commun..

[112] St Charles K.M., Ssematimba A., Malladi S., Bonney P.J., Linskens E., Culhane M., Goldsmith T.J., Halvorson D.A., Cardona C.J. (2018). Avian Influenza in the U.S. Commercial Upland Game Bird Industry: An Analysis of Selected Practices as Potential Exposure Pathways and Surveillance System Data Reporting. Avian Dis..

[113] Capua I., Marangon S. (2006). Control. of Avian Influenza in Poultry. Emerg. Infect. Dis..

[114] Parvin R., Nooruzzaman M., Kabiraj C.K., Begum J.A., Chowdhury E.H., Islam M.R., Harder T. (2020). Controlling Avian Influenza Virus in Bangladesh: Challenges and Recommendations. Viruses.

[115] OIE (2021). Avian Influenza.

[116] Peyre M., Fusheng G., Desvaux S., Roger F. (2009). Avian Influenza Vaccines: A Practical Review in Relation to Their Application in the Field with a Focus on the Asian Experience. Epidemiol. Infect..

[117] Capua I., Marangon S. (2000). The Avian Influenza Epidemic in Italy, 1999–2000: A review. Avian Pathol..

[118] Suarez D.L. (2005). Overview of Avian Influenza DIVA Test Strategies. Biologicals.

[119] Hasan N.H., Ignjatovic J., Peaston A., Hemmatzadeh F. (2016). Avian Influenza Virus and DIVA Strategies. Viral. Immunol..

[120] Kim S.H., Samal S.K. (2019). Innovation in Newcastle Disease Virus Vectored Avian Influenza Vaccines. Viruses.

[121] Cardona C.J., Halvorson D.A., Hall J., Pantin-Jackwood M.J., Brown J.D. (2020). Conducting Influenza Virus Pathogenesis Studies in Avian Species. Methods Mol. Biol..

[122] Driskell E.A., Jones C.A., Stallknecht D.E., Howerth E.W., Tompkins S.M. (2010). Avian Influenza Virus Isolates from Wild Birds Replicate and Cause Disease in a Mouse Model of Infection. Virology.

[123] Kim S.M., Kim Y.I., Pascua P.N., Choi Y.K. (2016). Avian Influenza A Viruses: Evolution and Zoonotic Infection. Semin. Respir. Crit. Care Med..

[124] Koo B.S., Kim H.K., Song D., Na W., Song M.S., Kwon J.J., Wong S.S., Noh J.Y., Ahn M.J., Kim D.J. (2018). Virological and Pathological Characterization of an Avian H1N1 Influenza A Virus. Arch. Virol..

[125] Li R., Yuan B., Xia X., Zhang S., Du Q., Yang C., Li N., Zhao J., Zhang Y., Zhang R. (2018). Tree Shrew as a New Animal Model to Study the Pathogenesis of Avian Influenza (H9N2) Virus Infection. Emerg. Microbes Infect..

[126] Pulit-Penaloza J.A., Brock N., Pappas C., Sun X., Belser J.A., Zeng H., Tumpey T.M., Maines T.R. (2020). Characterization of Highly Pathogenic Avian Influenza H5Nx Viruses in the Ferret Model. Sci. Rep..

[127] Veldhuis Kroeze E., Bauer L., Caliendo V., van Riel D. (2021). In Vivo Models to Study the Pathogenesis of Extra-Respiratory Complications of Influenza A Virus Infection. Viruses.

[128] Zanin M., Koçer Z.A., Poulson R.L., Gabbard J.D., Howerth E.W., Jones C.A., Friedman K., Seiler J., Danner A., Kercher L. (2017). Potential for Low-Pathogenic Avian H7 Influenza A Viruses to Replicate and Cause Disease in a Mammalian Model. J. Virol..

[129] Lei H., Lu X., Li S., Ren Y. (2021). High Immune Efficacy against Different Avian Influenza H5N1 Viruses Due to Oral Administration of a Saccharomyces Cerevisiae-Based Vaccine in Chickens. Sci. Rep..

[130] Germeraad E.A., Sanders P., Hagenaars T.J., Jong M.C.M., Beerens N., Gonzales J.L. (2019). Virus Shedding of Avian Influenza in Poultry: A Systematic Review and Meta-Analysis. Viruses.

[131] Mei K., Guo Y., Zhu X., Qu N., Huang J., Chen Z., Zhang Y., Zhao B., He Z., Liao M. (2019). Different Pathogenicity and Transmissibility of Goose-Origin H5N6 Avian Influenza Viruses in Chickens. Viruses.

[132] Cardona C.J., Halvorson D.A., Hall J., Pantin-Jackwood M.J., Brown J.D. (2020). Conducting Influenza Virus Pathogenesis Studies in Avian Species, in Animal Influenza Virus.

[133] Roy P., Noad R., Roy P. (2006). Bluetongue Virus Assembly and Morphogenesis. Reoviruses: Entry, Assembly and Morphogenesis.

[134] Schwartz-Cornil I., Mertens P., Contreras V., Hemati B., Pascale F., Bréard E., Mellor P., MacLachlan N., Zientara S. (2008). Bluetongue Virus: Virology, Pathogenesis and Immunity. Vet. Res..

[135] Prasad B.V., Yamaguchi S., Roy P. (1992). Three-Dimensional Structure of Single-Shelled Bluetongue Virus. J. Virol..

[136] Jones A.E., Turner J., Caminade C., Heath A.E., Wardeh M., Kluiters G., Diggle P.J., Morse A.P., Baylis M. (2019). Bluetongue Risk under Future Climates. Nat. Clim. Chang..

[137] Rao P.P., Hegde N.R., Singh K.P., Putty K., Hemadri D., Maan N.S., Reddy Y.N., Maan S., Mertens P.P.C., Bayry J. (2007). Bluetongue: Aetiology, Epidemiology, Pathogenesis, Diagnosis and Control., in Emerging and Re-Emerging Infectious Diseases of Livestock.

[138] Walton T.E. (2004). The History of Bluetongue and a Current Global Overview. Vet. Ital..

[139] Maclachlan N.J., Guthrie A.J. (2010). Re-Emergence of Bluetongue, African Horse Sickness, and Other Orbivirus Diseases. Vet. Res..

[140] Ries C., Sharav T., Tseren-Ochir E.-O., Beer M., Hoffmann B. (2021). Putative Novel Serotypes ‘33’ and ‘35’ in Clinically Healthy Small Ruminants in Mongolia Expand the Group of Atypical BTV. Viruses.

[141] Bumbarov V., Golender N., Jenckel M., Wernike K., Beer M., Khinich E., Zalesky O., Erster O. (2020). Characterization of Bluetongue Virus Serotype 28. Transbound. Emerg. Dis..

[142] Marcacci M., Sant S., Mangone I., Goria M., Dondo A., Zoppi S., van Gennip R.G.P., Radaelli M.C., Cammà C., van Rijn P.A. (2018). One after the Other: A Novel Bluetongue Virus Strain Related to Toggenburg Virus Detected in the Piedmont Region (North-Western Italy), Extends the Panel of Novel Atypical BTV Strains. Transbound. Emerg. Dis..

[143] Ries C., Domes U., Janowetz B., Böttcher J., Burkhardt K., Miller T., Beer M., Hoffmann B. (2020). Isolation and Cultivation of a New Isolate of BTV-25 and Presumptive Evidence for a Potential Persistent Infection in Healthy Goats. Viruses.

[144] Rupner R.N., Vinodh Kumar R.O., Karthikeyan R., Sinha D.K., Singh K.P., Dubal Z.B., Tamta S., Gupta V.K., Singh B.R., Malik Y.S. (2020). Bluetongue in India: A Systematic Review and Meta-Analysis with Emphasis on Diagnosis and Seroprevalence. Vet. Q..

[145] Parsonson I.M. (1990). Pathology and Pathogenesis of Bluetongue Infections.

[146] Maclachlan N.J., Drew C.P., Darpel K.E., Worwa G. (2009). The Pathology and Pathogenesis of Bluetongue. J. Comp. Pathol..

[147] Darpel K.E., Batten C.A., Veronesi E., Shaw A.E., Anthony S., Bachanek-Bankowska K., Kgosana L., Bin-Tarif A., Carpenter S., Müller-Doblies U.U. (2007). Clinical Signs and Pathology Shown by British Sheep and Cattle Infected with Bluetongue Virus Serotype 8 Derived from the 2006 Outbreak in Northern Europen. Vet. Rec..

[148] Sperlova A., Zendulkova D. (2011). Bluetongue: A Review. Vet. Med..

[149] Linden A., Gregoire F., Nahayo A., Hanrez D., Mousset B., Massart A.L., de Leeuw I., Vandemeulebroucke E., Vandenbussche F., de Clercq K. (2010). Bluetongue Virus in Wild Deer, Belgium, 2005–2008. Emerg. Infect. Dis..

[150] Ruiz-Fons F., Sánchez-Matamoros A., Gortázar C., Sánchez-Vizcaíno J.M. (2014). The Role of Wildlife in Bluetongue Virus Maintenance in Europe: Lessons Learned after the Natural Infection in Spain. Virus Res..

[151] Wilbur L.A., Evermann J.F., Levings R.L., Stoll I.R., Starling D.E., Spillers C.A., Gustafson G.A., McKeirnan A.J. (1994). Abortion and Death in Pregnant Bitches Associated with a Canine Vaccine Contaminated with Bluetongue Virus. J. Am. Vet. Med. Assoc..

[152] Alexander K.A., MacLachlan N.J., Kat P.W., House C., O’Brien S.J., Lerche N.W., Sawyer M., Frank L.G., Holekamp K., Smale L. (1994). Evidence of Natural Bluetongue Virus Infection among African Carnivores. Am. J. Trop. Med. Hyg..

[153] Maclachlan N.J. (2010). Global Implications of the Recent Emergence of Bluetongue Virus in Europe. Vet. Clin. N. Am. Food Anim. Pract..

[154] MacLachlan N.J., Osburn B.I. (2006). Impact of Bluetongue Virus Infection on the International Movement and Trade of Ruminants. J. Am. Vet. Med Assoc..

[155] Velthuis A.G.J., Mourits M.C.M., Saatkamp H.W., de Koeijer A.A., Elbers A.R.W. (2011). Financial Evaluation of Different Vaccination Strategies for Controlling the Bluetongue Virus Serotype 8 Epidemic in the Netherlands in 2008. PLoS ONE.

[156] Pinior B., Lebl K., Firth C., Rubel F., Fuchs R., Stockreiter S., Loitsch A., Köfer J. (2015). Cost Analysis of Bluetongue Virus Serotype 8 Surveillance and Vaccination Programmes in Austria from 2005 to 2013. Vet. J..

[157] Mayo C., Lee J., Kopanke J., MacLachlan N.J. (2017). A Review of Potential Bluetongue Virus Vaccine Strategies. Vet. Microbiol..

[158] Coetzee P., van Vuuren M., Venter E.H., Stokstad M. (2014). A Review of Experimental Infections with Bluetongue Virus in the Mammalian Host. Virus Res..

[159] Martinelle L., Pozzo F.D., Thiry E., de Clercq K., Saegerman C. (2019). Reliable and Standardized Animal Models to Study the Pathogenesis of Bluetongue and Schmallenberg Viruses in Ruminant Natural Host Species with Special Emphasis on Placental Crossing. Viruses.

[160] Maclachlan N.J., Crafford J.E., Vernau W., Gardner I.A., Goddard A., Guthrie A.J., Venter E.H. (2008). Experimental Reproduction of Severe Bluetongue in Sheep. Vet. Pathol..

[161] Pini A. (1976). Study on the Pathogenesis of Bluetongue: Replication of the Virus in the Organs of Infected Sheep. Onderstepoort J. Vet. Res..

[162] Gibbs E.P.J., Greiner E.C. (1994). The Epidemiology of Bluetongue. Comp. Immunol. Microbiol. Infect. Dis..

[163] Melville L.F., Hunt N.T., Davis S.S., Weir R.P. (2004). Bluetongue Virus Does Not Persist in Naturally Infected Cattle. Vet. Ital..

[164] Dal Pozzo F., Saegerman C., Thiry E. (2009). Bovine Infection with Bluetongue Virus with Special Emphasis on European Serotype 8. Vet. J..

[165] Barratt-Boyes S.M., MacLachlan N.J. (1994). Dynamics of Viral Spread in Bluetongue Virus Infected Calves. Vet. Microbiol..

[166] Caporale M., Wash R., Pini A., Savini G., Franchi P., Golder M., Patterson-Kane J., Mertens P., di Gialleonardo L., Armillotta G. (2011). Determinants of Bluetongue Virus Virulence in Murine Models of Disease. J. Virol..

[167] Stokstad M., Coetzee P., Myrmel M., Mutowembwa P., Venter E.H., Larsen S. (2021). Refined Experimental Design May Increase the Value of Murine Models for Estimation of Bluetongue Virus Virulence. Lab. Anim..

[168] Narayan O., Johnson R.T. (1972). Effects of Viral Infection on Nervous System Development. I. Pathogenesis of Bluetongue Virus Infection in Mice. Am. J. Pathol..

[169] Calvo-Pinilla E., Rodríguez-Calvo T., Anguita J., Sevilla N., Ortego J. (2009). Establishment of a Bluetongue Virus Infection Model in Mice that Are Deficient in the Alpha/Beta Interferon Receptor. PLoS ONE.

[170] Ortego J., de la Poza F., Marín-López A. (2014). Interferon α/β Receptor Knockout Mice as a Model to Study Bluetongue Virus Infection. Virus Res..

[171] Darpel K.E., Monaghan P., Simpson J., Anthony S.J., Veronesi E., Brooks H.W., Elliott H., Brownlie J., Takamatsu H.-H., Mellor P.S. (2012). Involvement of the Skin During Bluetongue Virus Infection and Replication in the Ruminant Host. Vet. Res..

[172] Umeshappa C.S., Singh K.P., Channappanavar R., Sharma K., Nanjundappa R.H., Saxena M., Singh R., Sharma A.K. (2011). A Comparison of Intradermal and Intravenous Inoculation of Bluetongue Virus Serotype 23 in Sheep for Clinico-Pathology, and Viral and Immune Responses. Vet. Immunol. Immunopathol..

[173] Blome S., Staubach C., Henke J., Carlson J., Beer M. (2017). Classical Swine Fever-An Updated Review. Viruses.

[174] Moennig V., Becher P., Beer M. (2013). Classical Swine Fever. Dev. Biol..

[175] Moennig V., Floegel-Niesmann G., Greiser-Wilke I. (2003). Clinical Signs and Epidemiology of Classical Swine Fever: A Review of New Knowledge. Vet. J..

[176] Penrith M.L., Vosloo W., Mather C. (2011). Classical Swine Fever (Hog Cholera): Review of Aspects Relevant to Control. Transbound. Emerg. Dis..

[177] Summerfield A., Ruggli N. (2015). Immune Responses Against Classical Swine Fever Virus: Between Ignorance and Lunacy. Front. Vet. Sci..

[178] Li S., Wang J., Yang Q., Anwar M.N., Yu S., Qiu H.J. (2017). Complex Virus-Host Interactions Involved in the Regulation of Classical Swine Fever Virus Replication: A Minireview. Viruses.

[179] Zhang L., Qin Y., Chen M. (2018). Viral Strategies for Triggering and Manipulating Mitophagy. Autophagy.

[180] Zhu E., Chen W., Qin Y., Ma S., Fan S., Wu K., Li W., Fan J., Yi L., Ding H. (2019). Classical Swine Fever Virus Infection Induces Endoplasmic Reticulum Stress-Mediated Autophagy to Sustain Viral Replication In Vivo and In Vitro. Front. Microbiol..

[181] Leifer I., Ruggli N., Blome S. (2013). Approaches to Define the Viral Genetic Basis of Classical Swine Fever Virus Virulence. Virology.

[182] Ma S.M., Mao Q., Yi L., Zhao M.Q., Chen J.D. (2019). Apoptosis, Autophagy, and Pyroptosis: Immune Escape Strategies for Persistent Infection and Pathogenesis of Classical Swine Fever Virus. Pathogens.

[183] Vuono E.A., Ramirez-Medina E., Azzinaro P., Berggren K.A., Rai A., Pruitt S., Silva E., Velazquez-Salinas L., Borca M.V., Gladue D.P. (2020). SERTA Domain Containing Protein 1 (SERTAD1) Interacts with Classical Swine Fever Virus Structural Glycoprotein E2, Which Is Involved in Virus Virulence in Swine. Viruses.

[184] Liu C.C., Liu Y.Y., Cheng Y., Zhang Y.N., Zhang J., Liang X.D., Gao Y., Chen H., Baloch A.S., Yang Q. (2021). The ESCRT-I Subunit Tsg101 Plays Novel Dual Roles in Entry and Replication of Classical Swine Fever Virus. J. Virol..

[185] Dahle J., Liess B. (1992). A Review on Classical Swine Fever Infections in Pigs: Epizootiology, Clinical Disease and Pathology. Comp. Immunol. Microbiol. Infect. Dis..

[186] Fatima M., Luo Y., Zhang L., Wang P.-Y., Song H., Fu Y., Li Y., Sun Y., Li S., Bao Y.-J. (2021). Genotyping and Molecular Characterization of Classical Swine Fever Virus Isolated in China during 2016–2018. Viruses.

[187] Stoian A.M.M., Petrovan V., Constance L.A., Olcha M., Dee S., Diel D.G., Sheahan M.A., Rowland R.R.R., Patterson G., Niederwerder M.C. (2020). Stability of Classical Swine Fever Virus and Pseudorabies Virus in Animal Feed Ingredients Exposed to Transpacific Shipping Conditions. Transbound. Emerg. Dis..

[188] Greiser-Wilke I., Blome S., Moennig V. (2007). Diagnostic Methods for Detection of Classical Swine Fever Virus--Status Quo and New Developments. Vaccine.

[189] De Smit A.J. (2000). Laboratory Diagnosis, Epizootiology, and Efficacy of Marker Vaccines in Classical Swine Fever: A Review. Vet. Q..

[190] Wang L., Madera R., Li Y., McVey D.S., Drolet B.S., Shi J. (2020). Recent Advances in the Diagnosis of Classical Swine Fever and Future Perspectives. Pathogens.

[191] Beemer O., Remmenga M., Gustafson L., Johnson K., Hsi D., Antognoli M.C. (2019). Assessing the Value of PCR Assays in Oral Fluid Samples for Detecting African Swine Fever, Classical Swine Fever, and Foot-and-Mouth Disease in U.S. Swine. PLoS ONE.

[192] Chander V., Nandi S., Ravishankar C., Upmanyu V., Verma R. (2014). Classical Swine Fever in Pigs: Recent Developments and Future Perspectives. Anim. Health Res. Rev..

[193] OIE (2019). Classical swine fever (infection with classical swine fever). Terrestrial Animal Health Manual.

[194] Pineda P., Santa C., Deluque A., Peña M., Casal J. (2021). Evaluation of the Sensitivity of the Classical Swine Fever Surveillance System in Two Free Zones in Colombia. Transbound. Emerg. Dis..

[195] Meyer D., Petrov A., Becher P. (2019). Inactivation of Classical Swine Fever Virus in Porcine Serum Samples Intended for Antibody Detection. Pathogens.

[196] Bohórquez J.A., Muñoz-González S., Pérez-Simó M., Muñoz I., Rosell R., Coronado L., Domingo M., Ganges L. (2020). Foetal Immune Response Activation and High. Replication Rate during Generation of Classical Swine Fever Congenital Infection. Pathogens.

[197] Muñoz-González S., Ruggli N., Rosell R., Pérez L.J., Frías-Leuporeau M.T., Fraile L., Montoya M., Cordoba L., Domingo M., Ehrensperger F. (2015). Postnatal Persistent Infection with Classical Swine Fever Virus and Its Immunological Implications. PLoS ONE.

[198] Kleiboeker S.B. (2002). Swine Fever: Classical Swine Fever and African Swine Fever. Vet. Clin. N. Am. Food Anim. Pract..

[199] Gómez-Villamandos J.C., Carrasco L., Bautista M.J., Sierra M.A., Quezada M., Hervas J., Mde L.C., Ruiz-Villamor E., Salguero F.J., Sónchez-Cordón P.J. (2003). African Swine Fever and Classical Swine Fever: A Review of the Pathogenesis. Dtsch. Tierarztl. Wochenschr..

[200] Coronado L., Bohórquez J.A., Muñoz-González S., Perez L.J., Rosell R., Fonseca O., Delgado L., Perera C.L., Frías M.T., Ganges L. (2019). Investigation of Chronic and Persistent Classical Swine Fever Infections under Field Conditions and Their Impact on Vaccine Efficacy. BMC Vet. Res..

[201] Barman N.N., Khatoon E., Bora M., Deori L., Gogoi S.M., Kalita D. (2021). Investigation of Congenital Tremor Associated with Classical Swine Fever Virus Genotype 2.2 in an Organized Pig Farm in North-Eastern India. Virus Dis..

[202] Beer M., Goller K.V., Staubach C., Blome S. (2015). Genetic Variability and Distribution of Classical Swine Fever Virus. Anim. Health Res. Rev..

[203] Paton D.J., McGoldrick A., Greiser-Wilke I., Parchariyanon S., Song J.Y., Liou P.P., Stadejek T., Lowings J.P., Björklund H., Belák S. (2000). Genetic Typing of Classical Swine Fever Virus. Vet. Microbiol..

[204] Hao G., Zhang H., Chen H., Qian P., Li X. (2020). Comparison of the Pathogenicity of Classical Swine Fever Virus Subgenotype 2.1c and 2.1d Strains from China. Pathogens.

[205] Postel A., Austermann-Busch S., Petrov A., Moennig V., Becher P. (2018). Epidemiology, Diagnosis and Control of Classical Swine Fever: Recent Developments and Future Challenges. Transbound. Emerg. Dis..

[206] Malik Y.S., Bhat S., Kumar O.R.V., Yadav A.K., Sircar S., Ansari M.I., Sarma D.K., Rajkhowa T.K., Ghosh S., Dhama K. (2020). Classical Swine Fever Virus Biology, Clinicopathology, Diagnosis, Vaccines and a Meta-Analysis of Prevalence: A Review from the Indian Perspective. Pathogens.

[207] Li Y.C., Chiou M.T., Lin C.N. (2020). Serodynamic Analysis of the Piglets Born from Sows Vaccinated with Modified Live Vaccine or E2 Subunit Vaccine for Classical Swine Fever. Pathogens.

[208] Cao T., Wang Z., Li X., Zhang S., Paudyal N., Zhang X., Li X., Fang W. (2019). E2 and E(Rns) of Classical Swine Fever Virus C-Strain Play Central Roles in Its Adaptation to Rabbits. Virus Genes.

[209] Cao T., Zhang S., Li X., Xu Y., Wang Z., Chen C., Paudyal N., Li X., Sun J., Fang W. (2019). Classical Swine Fever Virus C-Strain with Eight Mutation Sites Shows Enhanced Cell Adaptation and Protects Pigs from Lethal Challenge. Arch. Virol..

[210] Xie L., Han Y., Ma Y., Yuan M., Li W., Li L.F., Li M., Sun Y., Luo Y., Li S. (2020). P108 and T109 on E2 Glycoprotein Domain I Are Critical for the Adaptation of Classical Swine Fever Virus to Rabbits but Not for Virulence in Pigs. J. Virol..

[211] Coronado L., Perera C.L., Rios L., Frías M.T., Pérez L.J. (2021). A Critical Review about Different Vaccines against Classical Swine Fever Virus and Their Repercussions in Endemic Regions. Vaccines.

[212] Wei Q., Liu Y., Zhang G. (2021). Research Progress and Challenges in Vaccine Development against Classical Swine Fever Virus. Viruses.

[213] Lamothe-Reyes Y., Bohórquez J.A., Wang M., Alberch M., Pérez-Simó M., Rosell R., Ganges L. (2021). Early and Solid Protection Afforded by the Thiverval Vaccine Provides Novel Vaccination Alternatives Against Classical Swine Fever Virus. Vaccines.

[214] Jelsma T., Post J., Born E.v.d., Segers R., Kortekaas J.J.V. (2021). Assessing the Protective Dose of a Candidate DIVA Vaccine against Classical Swine Fever. Vaccines.

[215] Villanueva-Cabezas J.P., Wangchuk J. (2020). Classical Swine Fever: Challenges for the Emerging Swine Sector in Bhutan. Trop. Anim. Health Prod..

[216] Singh V.K., Rajak K.K., Kumar A., Yadav S.K. (2018). Classical Swine Fever in India: Current Status and Future Perspective. Trop. Anim. Health Prod..

[217] Huang Y.L., Deng M.C., Wang F.I., Huang C.C., Chang C.Y. (2014). The Challenges of Classical Swine Fever Control: Modified Live and E2 Subunit Vaccines. Virus Res..

[218] Je S.H., Kwon T., Yoo S.J., Lee D.U., Lee S., Richt J.A., Lyoo Y.S. (2018). Classical Swine Fever Outbreak after Modified Live LOM Strain Vaccination in Naive Pigs, South Korea. Emerg. Infect. Dis..

[219] Jang G., Kim J.A., Kang W.M., Yang H.S., Park C., Jeong K., Moon S.U., Park C.K., Lyoo Y.S., Lee C. (2019). Endemic Outbreaks Due to the Re-Emergence of Classical Swine Fever after Accidental Introduction of Modified Live LOM Vaccine on Jeju Island, South Korea. Transbound. Emerg. Dis..

[220] Blome S., Moß C., Reimann I., König P., Beer M. (2017). Classical Swine Fever Vaccines-State-of the-Art. Vet. Microbiol..

[221] Blome S., Wernike K., Reimann I., König P., Moß C., Beer M. (2017). A Decade of Research into Classical Swine Fever Marker Vaccine CP7_E2alf (Suvaxyn^®^ CSF Marker): A Review of Vaccine Properties. Vet. Res..

[222] Sordo-Puga Y., Suárez-Pedroso M., Naranjo-Valdéz P., Pérez-Pérez D., Santana-Rodríguez E., Sardinas-Gonzalez T., Mendez-Orta M.K., Duarte-Cano C.A., Estrada-Garcia M.P., Rodríguez-Moltó M.P. (2021). Porvac^®^ Subunit Vaccine E2-CD154 Induces Remarkable Rapid Protection against Classical Swine Fever Virus. Vaccines.

[223] Park Y., Oh Y., Wang M., Ganges L., Bohórquez J.A., Park S., Gu S., Park J., Lee S., Kim J. (2021). A Novel E2 Glycoprotein Subunit Marker Vaccine Produced in Plant Is Able to Prevent Classical Swine Fever Virus Vertical Transmission after Double Vaccination. Vaccines.

[224] Tong W., Zheng H., Li G.X., Gao F., Shan T.L., Zhou Y.J., Yu H., Jiang Y.F., Yu L.X., Li L.W. (2020). Recombinant Pseudorabies Virus Expressing E2 of Classical Swine Fever Virus (CSFV) Protects against Both Virulent Pseudorabies Virus and CSFV. Antivir. Res..

[225] Kumar R., Kumar V., Kekungu P., Barman N.N., Kumar S. (2019). Evaluation of Surface Glycoproteins of Classical Swine Fever Virus as Immunogens and Reagents for Serological Diagnosis of Infections in Pigs: A Recombinant Newcastle Disease Virus Approach. Arch. Virol..

[226] Ganges L., Crooke H.R., Bohórquez J.A., Postel A., Sakoda Y., Becher P., Ruggli N. (2020). Classical Swine Fever Virus: The Past, Present and Future. Virus Res..

[227] Backer J., Brouwer H., van Schaik G., Van Roermund H. (2011). Using Mortality Data for Early Detection of Classical Swine Fever in The Netherlands. Prev. Vet. Med..

[228] Backer J.A., Hagenaars T.J., van Roermund H.J., de Jong M.C. (2009). Modelling the Effectiveness and Risks of Vaccination Strategies to Control Classical Swine Fever Epidemics. J. R. Soc. Interface.

[229] Fan J., Liao Y., Zhang M., Liu C., Li Z., Li Y., Li X., Wu K., Yi L., Ding H. (2021). Anti-Classical Swine Fever Virus Strategies. Microorganisms.

[230] Artois M., Depner K., Guberti V., Hars J., Rossi S., Rutili D. (2002). Classical Swine Fever (Hog Cholera) in Wild Boar in Europe. Rev. Sci. Tech. l’OIE.

[231] Isoda N., Baba K., Ito S., Ito M., Sakoda Y., Makita K. (2020). Dynamics of Classical Swine Fever Spread in Wild Boar in 2018–2019, Japan. Pathogens.

[232] Ito S., Jurado C., Bosch J., Sánchez-Vizcaíno J.M., Isoda N., Sakoda Y. (2019). Role of Wild Boar in the Spread of Classical Swine Fever in Japan. Pathogens.

[233] Moennig V. (2015). The Control of Classical Swine Fever in Wild Boar. Front. Microbiol..

[234] Shimizu Y., Hayama Y., Murato Y., Sawai K., Yamaguchi E., Yamamoto T. (2020). Epidemiology of Classical Swine Fever in Japan—A Descriptive Analysis of the Outbreaks in 2018–2019. Front. Vet. Sci..

[235] Shimizu Y., Hayama Y., Murato Y., Sawai K., Yamaguchi E., Yamamoto T. (2021). Epidemiological Analysis of Classical Swine Fever in Wild Boars in Japan. BMC Vet. Res..

[236] Kameyama K.I., Nishi T., Yamada M., Masujin K., Morioka K., Kokuho T., Fukai K. (2019). Experimental Infection of Pigs with a Classical Swine Fever Virus Isolated in Japan for the First Time in 26 Years. J. Vet. Med. Sci..

[237] Jelsma T., Wijnker J.J., Smid B., Verheij E., van der Poel W.H.M., Wisselink H.J. (2019). Salt Inactivation of Classical Swine Fever Virus and African Swine Fever Virus in Porcine Intestines Confirms the Existing In Vitro Casings Model. Vet. Microbiol..

[238] Badasara S.K., Mohan M., Sah V., Kumari P., Upmanyu V., Dhar P., Tiwari A.K., Chander V., Gupta V.K. (2017). Replacement of Animal Model for Propagation of Classical Swine Fever Challenge Virus by Adaption in the PK-15 Cell Line. J. Anim. Res..

[239] Sordo Y., Suárez M., Caraballo R., Sardina T., Brown E., Duarte C., Lugo J., Gil L., Perez D., Oliva A. (2018). Humoral and Cellular Immune Response in Mice Induced by the Classical Swine Fever Virus E2 Protein Fused to the Porcine CD154 Antigen. Biologicals.

[240] Di Teodoro G., Marruchella G., Di Provvido A., D’Angelo A.R., Orsini G., Di Giuseppe P., Sacchini F., Scacchia M. (2020). Contagious Bovine Pleuropneumonia: A Comprehensive Overview. Vet. Pathol..

[241] Amanfu W., Kardjadj M. (2019). Contagious Bovine Pleuropneumonia, in Transboundary Animal Diseases in Sahelian Africa and Connected Regions.

[242] Dupuy V., Manso-Silván L., Barbe V., Thebault P., Dordet-Frisoni E., Citti C., Poumarat F., Blanchard A., Breton M., Sirand-Pugnet P. (2012). Evolutionary History of Contagious Bovine Pleuropneumonia Using Next Generation Sequencing of *Mycoplasma Mycoides* Subsp. Mycoides “Small Colony”. PLoS ONE.

[243] Jores J., Baldwin C., Blanchard A., Browning G.F., Colston A., Gerdts V., Goovaerts D., Heller M., Juleff N., Labroussaa F. (2020). Contagious Bovine and Caprine Pleuropneumonia: A Research Community’s Recommendations for the Development of Better Vaccines. NPJ Vaccines.

[244] Amanfu W. (2009). Contagious Bovine Pleuropneumonia (Lung Sickness) in Africa. Onderstepoort J. Vet. Res..

[245] Dedieu-Engelmann L. (2008). Contagious Bovine Pleuropneumonia: A Rationale for the Development of a Mucosal Sub-Unit Vaccine. Comp. Immunol. Microbiol. Infect. Dis..

[246] Thiaucourt F., Yaya A., Wesonga H., Huebschle O.J., Tulasne J.J., Provost A. (2006). Contagious Bovine Pleuropneumonia: A Reassessment of the Efficacy of Vaccines Used in Africa. Ann. N. Y. Acad. Sci..

[247] Olorunshola I.D., Peters A.R., Scacchia M., Nicholas R.A.J. (2017). Contagious Bovine Pleuropneumonia—Never Out of Africa?. CAB Rev..

[248] Mamo Y., Bitew M., Teklemariam T., Soma M., Gebre D., Abera T., Benti T., Deneke Y. (2018). Contagious Bovine Pleuropneumonia: Seroprevalence and Risk Factors in Gimbo District, Southwest Ethiopia. Vet. Med. Int..

[249] Zerbo L.H., Dahourou L.D., Sidi M., Ouoba L.B., Ouandaogo S.H., Bazimo G., Sie B.N., Traore K.Z.A., Tapsoba M., Ouedraogo A. (2021). Seroprevalence and Determinants of Contagious Bovine Pleuropneumonia in Cattle in Burkina Faso. Trop. Anim. Health Prod..

[250] Nicholas R., Bashiruddin J., Ayling R., Miles R. (2000). Contagious Bovine Pleuropneumonia: A Review of Recent Developments. Vet. Bull..

[251] Abdela N., Yune N. (2017). Seroprevalence and Distribution of Contagious Bovine Pleuropneumonia in Ethiopia: Update and Critical Analysis of 20 Years (1996–2016) Reports. Front. Vet. Sci..

[252] Yansambou M.S., Diallo A.A., Idi M., Gagara H., Haido A.M., Alambedji R.B. (2018). Serological Prevalence of Contagious Bovine Pleuropneumonia in Niger in 2017. Front. Vet. Sci..

[253] Thiaucourt F., van der Lugt J.J., Provost A., Coetzer J.A., Tustin R.C. (2004). Contagious Bovine Pleuropneumonia. Infectious Diseases of Livestock.

[254] Masiga W., Domenech J., Windsor R. (1996). Manifestation and Epidemiology of Contagious Bovine Pleuropneumonia in Africa. Rev. Sci. Tech..

[255] Windsor R., Masiga W. (1977). Investigations into the Role of Carrier Animals in the Spread of Contagious Bovine Pleuropneumonia. Res. Vet. Sci..

[256] Lesnoff M., Bonnet P., Workalemahu A. (2004). A Mathematical Model of Contagious Bovine Pleuropneumonia (CBPP) within-Herd Outbreaks for Economic Evaluation of Local Control Strategies: An Illustration from a Mixed Crop-Livestock System in Ethiopian Highlands. Anim. Res..

[257] Windsor R.S. (2006). The Eradication of Contagious Bovine Pleuropneumonia from South Western Africa: A Plan for Action. Ann. New York Acad. Sci..

[258] Le Goff C., Thiaucourt F. (1998). A Competitive ELISA for the Specific Diagnosis of Contagious Bovine Pleuropneumonia (CBPP). Vet. Microbiol..

[259] Ayling R.D., Bisgaard-Frantzen S., March J.B., Godinho K., Nicholas R.A.J. (2005). Assessing the In Vitro Effectiveness of Antimicrobials against *Mycoplasma Mycoides* Subsp. Mycoides Small-Colony Type to Reduce Contagious Bovine Pleuropneumonia Infection. Antimicrob. Agents Chemother..

[260] Huebschle O.J., Ayling R.D., Godinho K., Lukhele O., Tjipura-Zaire G., Rowan T.G., Nicholas R.A. (2006). Danofloxacin (Advocin™) Reduces the Spread of Contagious Bovine Pleuropneumonia to Healthy In-Contact Cattle. Res. Vet. Sci..

[261] Danbirni S., Kaltungo B.Y., Dandu B.B., Mohammed F.U., Abubakar U.B., Ibrahim S., Usman A., Sackey A.K.B. (2020). Clinical Management Protocol of an Acute Contagious Bovine Pleuropneumonia in a 6-Year-Old Bunaji Cow in Sakaru Village of Soba Local Gov-Ernment Area, Kaduna State, Nigeria. Niger. J. Anim. Prod..

[262] Tulasne J.J., Litamoi J.K., Morein B., Dedieu L., Palya V.J., Yami M., Abusugra I., Sylla D., Bensaid A. (1996). Contagious Bovine Pleuropneumonia Vaccines: The Current Situation and the Need for Improvement. Rev. Sci. Tech..

[263] Sacchini F., Luciani M., Salini R., Scacchia M., Pini A., Lelli R., Naessens J., Poole J., Jores J. (2012). Plasma lLevels of TNF-alpha, IFN-gamma, IL-4 and IL-10 during a Course of Experimental Contagious Bovine Pleuropneumonia. BMC Vet. Res..

[264] Nicholas R.A.J., Ayling R.D., Tjipura-Zaire G., Rowan T. (2012). Treatment of Contagious Bovine Pleuropneumonia. Vet. Rec..

[265] Hamsten C., Tjipura-Zaire G., McAuliffe L., Huebschle O.J.B., Scacchia M., Ayling R.D., Persson A. (2010). Protein-Specific Analysis of Humoral Immune Responses in a Clinical Trial for Vaccines against Contagious Bovine Pleuropneumonia. Clin. Vaccine Immunol..

[266] Jores J., Mariner J.C., Naessens J. (2013). Development of an Improved Vaccine for Contagious Bovine Pleuropneumonia: An African Perspective on Challenges and Proposed Actions. Vet. Res..

[267] Westberg J., Persson A., Holmberg A., Goesmann A., Lundeberg J., Johansson K.-E., Pettersson B., Uhlén M. (2004). The Genome Sequence of *Mycoplasma Mycoides* Subsp. Mycoides SC Type Strain PG1T, the Causative Agent of Contagious Bovine Pleuropneumonia (CBPP). Genome Res..

[268] Windsor R.S., Wood A. (1998). Contagious Bovine Pleuropneumonia: The Costs of Control in Central/Southern Africa. Ann. New York Acad. Sci..

[269] Ter Laak E. (1992). Contagious Bovine Pleuropneumonia A Review. Vet. Q..

[270] Nicholas R., Bashiruddin J. (1995). Mycoplasma Mycoides Subspecies Mycoides (Small Colony Variant): The Agent of Contagious Bovine Pleuropneumonia and Member of the “Mycoplasma Mycoides Cluster”. J. Comp. Pathol..

[271] Alhaji N.B., Ankeli P.I., Ikpa L.T., Babalobi O.O. (2020). Contagious Bovine Pleuropneumonia: Challenges and Prospects Regarding Diagnosis and Control Strategies in Africa. Vet. Med. Res. Rep..

[272] Marobela-Raborokgwe C., Modise B., Kgotlele T., Masoba K., Keokilwe L., Dipuo K. (2018). The Experience of Contagious Bovine Pleuropneumonia Ring Trials in Botswana. Rev. Sci. Tech..

[273] Rweyemamu M., Litamoi J., Palya V., Sylla D. (1995). Contagious Bovine Pleuropneumonia Vaccines: The Need for Improvements. Rev. Sci. Tech..

[274] Ssematimba A., Jores J., Mariner J.C. (2015). Mathematical Modelling of the Transmission Dynamics of Contagious Bovine Pleuropneu-Monia Reveals Minimal Target Profiles for Improved Vaccines and Diagnostic Assays. PLoS ONE.

[275] Kusiluka L., Sudi F. (2003). Review of Successes and Failures of Contagious Bovine Pleuropneumonia Control Strategies in Tanzania. Prev. Vet. Med..

[276] Marobela-Raborokgwe C. (2011). Contagious Bovine Pleuropneumonia in Botswana: Experience with Control, Eradication, Prevention and Surveillance. Vet. Ital..

[277] Muuka G., Songolo N., Kabilika S., Hang’ombe B.M., Nalubamba K.S., Muma J.B. (2013). Challenges of Controlling Contagious Bovine Pleuropneumonia in Sub-Saharan Africa: A Zambian Perspective. Trop. Anim. Health Prod..

[278] Onono J., Wieland B., Suleiman A., Rushton J. (2017). Policy Analysis for Delivery of Contagious Bovine Pleuropneumonia Control Strategies in Sub-Saharan Africa. Rev. Sci. Tech..

[279] Tambi N.E., Maina W.O., Ndi C. (2006). An Estimation of the Economic Impact of Contagious Bovine Pleuropneumonia in Africa. Rev. Sci. Tech..

[280] Aligaz A.A., Munganga J.M.W. (2021). Modelling the Transmission Dynamics of Contagious Bovine Pleuropneumonia in the Presence of Antibiotic Treatment with Limited Medical Supply. Math. Model. Anal..

[281] Mariner J., McDermott J., Heesterbeek J., Thomson G., Martin S. (2006). A Model of Contagious Bovine Pleuropneumonia Transmission Dynamics in East Africa. Prev. Vet. Med..

[282] Mariner J., McDermott J., Heesterbeek J., Thomson G., Roeder P., Martin S. (2006). A Heterogeneous Population Model for Contagious Bovine Pleuropneumonia Transmission and Control in Pastoral Communities of East Africa. Prev. Vet. Med..

[283] Aligaz A.A., Munganga J.M.W. (2019). Mathematical Modelling of the Transmission Dynamics of Contagious Bovine Pleuropneumonia with Vaccination and Antibiotic Treatment. J. Appl. Math..

[284] Aye R., Weldearegay Y.B., Lutta H.O., Chuma F., Pich A., Jores J., Meens J., Naessens J. (2018). Identification of Targets of Monoclonal Antibodies that Inhibit Adhesion and Growth in Mycoplasma Mycoides Subspecies Mycoides. Vet. Immunol. Immunopathol..

[285] Gaurivaud P., Lakhdar L., le Grand D., Poumarat F., Tardy F. (2014). Comparison of In Vivo and In Vitro Properties of Capsulated and Noncapsulated Variants of Mycoplasma Mycoides Subsp. Mycoides Strain Afade: A Potential New Insight into the Biology of Contagious Bovine Pleuropneumonia. FEMS Microbiol. Lett..

[286] Di Teodoro G., Marruchella G., Di Provvido A., Orsini G., Ronchi G.F., D’Angelo A.R., D’Alterio N., Sacchini F., Scacchia M. (2018). Respiratory Explants as a Model to Investigate Early Events of Contagious Bovine Pleuropneumonia Infection. Vet. Res..

[287] Waite E., March J. (2002). Capsular Polysaccharide Conjugate Vaccines against Contagious Bovine Pleuropneumonia: Immune Responses and Protection in Mice. J. Comp. Pathol..

[288] Mwirigi M., Nkando I., Olum M., Attah-Poku S., Ochanda H., Berberov E., Potter A., Gerdts V., Perez-Casal J., Wesonga H. (2016). Capsular Polysaccharide from *Mycoplasma Mycoides* Subsp. Mycoides Shows Potential for Protection against Contagious Bovine Pleuropneumonia. Vet. Immunol. Immunopathol..

[289] Gull T., French R.A., Gorton T.S., Burrage T.G., Prozesky L., Geary S.J., Adams L.G. (2013). Models of Contagious Bovine Pleuro-pneumonia: Evaluation of Two Novel Strains. Open Vet. Sci. J..

[290] Nkando I.G., Wesonga H.O., Kuria J.K., McKeever D. (2010). Assessing the Effectiveness of Intubation as a Challenge Model in Conta-Gious Bovine Pleuropneumonia Vaccine Experiments. Trop. Anim. Health Prod..

[291] Sacchini F., Liljander A.M., Heller M., Poole E.J., Posthaus H., Schieck E., Jores J. (2020). Reproduction of Contagious Bovine Pleu-Ropneumonia via Aerosol-Based Challenge with *Mycoplasma Mycoides* Subsp. Mycoides. Acta Vet. Scand..

[292] Mahy B.W.J. (2005). Introduction and History of Foot-and-Mouth Disease Virus.

[293] Grubman M.J., Baxt B. (2004). Foot-and-Mouth Disease. Clin. Microbiol. Rev..

[294] Jamal S.M., Belsham G.J. (2013). Foot-and-Mouth Disease: Past, Present and Future. Vet. Res..

[295] Segundo F.D.-S., Medina G.N., Stenfeldt C., Arzt J., Santos T.D.L. (2017). Foot-and-Mouth Disease Vaccines. Vet. Microbiol..

[296] Alexandersen S., Mowat N. (2005). Foot-and-Mouth Disease: Host Range and Pathogenesis. Curr. Top. Microbiol..

[297] Thomson G., Vosloo W., Bastos A. (2003). Foot and Mouth Disease in Wildlife. Virus Res..

[298] Brito B.P., Rodriguez L.L., Hammond J.M., Pinto J., Perez A.M. (2017). Review of the Global Distribution of Foot-and-Mouth Disease Virus from 2007 to 2014. Transbound. Emerg. Dis..

[299] Knight-Jones T.J.D., Robinson L., Charleston B., Rodriguez L.L., Gay C.G., Sumption K.J., Vosloo W. (2016). Global Foot-and-Mouth Disease Research Update and Gap Analysis: 2—Epidemiology, Wildlife and Economics. Transbound. Emerg. Dis..

[300] Gibbs E.P., A Herniman K., Lawman M.J., Sellers R.F. (1975). Foot-and-Mouth Disease in British Deer: Transmission of Virus to Cattle, Sheep and Deer. Vet. Rec..

[301] Kittelberger R., Nfon C., Swekla K., Zhang Z., Hole K., Bittner H., Salo T., Goolia M., Embury-Hyatt C., Bueno R. (2017). Foot-and-Mouth Disease in Red Deer—Experimental Infection and Test. Methods Perform..

[302] Paton D.J., Gubbins S., King D.P. (2018). Understanding the Transmission of Foot-and-Mouth Disease Virus at Different Scales. Curr. Opin. Virol..

[303] Donaldson A.I., Gloster J., Harvey L.D., Deans D.H. (1982). Use of Prediction Models to Forecast and Analyse Airborne Spread during the Foot-and-Mouth Disease Outbreaks in Brittany, Jersey and the Isle of Wight in 1981. Vet. Rec..

[304] Knight-Jones T., Rushton J. (2013). The Economic Impacts of Foot and Mouth Disease—What Are They, how Big Are They and where Do They Occur?. Prev. Vet. Med..

[305] Davies G. (2002). The Foot and Mouth Disease (FMD) Epidemic in the United Kingdom 2001. Comp. Immunol. Microbiol. Infect. Dis..

[306] Doel T.R., Mahy B.W.J. (2005). Natural and Vaccine Induced Immunity to FMD. Foot-and-Mouth Disease Virus.

[307] Sutmoller P., Gaggero A. (1965). Foot-and Mouth Diseases Carriers. Vet. Rec..

[308] Ferris N., King D., Reid S., Hutchings G., Shaw A., Paton D., Goris N., Haas B., Hoffmann B., Brocchi E. (2006). Foot-and-Mouth Disease Virus: A First Inter-Laboratory Comparison Trial to Evaluate Virus Isolation and RT-PCR Detection Methods. Vet. Microbiol..

[309] Sobrino F., Dávila M., Ortín J., Domingo E. (1983). Multiple Genetic Variants Arise in the Course of Replication of Foot-and-Mouth Disease Virus in Cell Culture. Virology.

[310] Gray A.R., Wood B.A., Henry E., Azhar M., King D.P., Mioulet V. (2020). Evaluation of Cell Lines for the Isolation of Foot-and-Mouth Disease Virus and Other Viruses Causing Vesicular Disease. Front. Vet. Sci..

[311] Kopliku L., Relmy A., Romey A., Gorna K., Zientara S., Bakkali-Kassimi L., Blaise-Boisseau S. (2015). Establishment of Persistent Foot-and-Mouth Disease Virus (FMDV) Infection in MDBK Cells. Arch. Virol..

[312] O’Donnell V., Pacheco J., Larocco M., Gladue D., Pauszek S., Smoliga G., Krug P., Baxt B., Borca M., Rodriguez L. (2014). Virus–Host Interactions in Persistently FMDV-Infected Cells Derived from Bovine Pharynx. Virology.

[313] De la Torre J.C., Dávila M., Sobrino F., Ortín J., Domingo E. (1985). Establishment of Cell Lines Persistently Infected with Foot-and-Mouth Disease Virus. Virology.

[314] Hägglund S., Laloy E., Näslund K., Pfaff F., Eschbaumer M., Romey A., Relmy A., Rikberg A., Svensson A., Huet H. (2019). Model of Persistent Foot-and-Mouth Disease Virus Infection in Multilayered Cells Derived from Bovine Dorsal Soft Palate. Transbound. Emerg. Dis..

[315] Habiela M., Seago J., Perez-Martin E., Waters R., Windsor M., Salguero F.J., Wood J., Charleston B., Juleff N. (2014). Laboratory Animal Models to Study Foot-and-Mouth Disease: A Review with Emphasis on Natural and Vaccine-Induced Immunity. J. Gen. Virol..

[316] Weber D.J., Wolfson J.S., Swartz M.N., Hooper D.C. (1984). Pasteurella Multocida Infections. Report Of 34 Cases and Review of the Literature. Medicine.

[317] Shivachandra S.B., Viswas K.N., Kumar A.A. (2011). A Review of Hemorrhagic Septicemia in Cattle and Buffalo. Anim. Health Res. Rev..

[318] Biberstein E.L. (1990). Our Understanding of the Pasteurellaceae. Can. J. Vet. Res..

[319] Wilson B.A., Ho M. (2013). Pasteurella Multocida: From Zoonosis to Cellular Microbiology. Clin. Microbiol. Rev..

[320] Raffi F., Barrier J., Baron D., Drugeon H.B., Nicolas F., Courtieu A.L. (1987). Pasteurella Multocida Bacteremia: Report of Thirteen Cases over Twelve Years and Review of the Literature. Scand. J. Infect. Dis..

[321] Hunt M.L., Boucher D.J., Boyce J.D., Adler B. (2001). In Vivo-Expressed Genes of Pasteurella multocida. Infect. Immun..

[322] Hurtado R., Maturrano L., Azevedo V., Aburjaile F. (2020). Pathogenomics Insights for Understanding Pasteurella Multocida Adaptation. Int. J. Med. Microbiol..

[323] Peng Z., Wang X., Zhou R., Chen H., Wilson B.A., Wu B. (2019). Pasteurella Multocida: Genotypes and Genomics. Microbiol. Mol. Biol. Rev..

[324] Harper M., Boyce J., Adler B. (2006). Pasteurella Multocida Pathogenesis: 125 Years after Pasteur. FEMS Microbiol. Lett..

[325] Tankaew P., Srisawat W., Singhla T., Tragoolpua K., Kataoka Y., Sawada T., Sthitmatee N. (2018). Comparison of Two Indirect ELISA Coating Antigens for the Detection of Dairy Cow Antibodies Against Pasteurella Multocida. J. Microbiol. Methods.

[326] Harper M., Cox A., Adler B., Boyce J. (2011). Pasteurella Multocida Lipopolysaccharide: The Long and the Short of It. Vet. Microbiol..

[327] Johnson R.B., Dawkins H.J., Spencer T.L. (1991). Electrophoretic Profiles of Pasteurella Multocida Isolates from Animals with Hemor-Rhagic Septicemia. Am. J. Vet. Res..

[328] De Alwis M.C. (1992). Haemorrhagic Septicaemia—A General Review. Br. Vet. J..

[329] Eriksen L., Aalbaek B., Leifsson P.S., Basse A., Christiansen T., Eriksen E., Rimler R.B. (1999). Hemorrhagic Septicemia in Fallow Deer (Dama Dama) Caused by Pasteurella Multocida Multocida. J. Zoo Wildl. Med..

[330] Khairani K.O., Nydam D., Felippe M.J., McDonough P., Barry J., Mahmud R., Haryono M., Radcliffe R.W. (2018). Surveillance for Hemorrhagic Septicemia in Buffalo (Bubalus Bubalis) as an Aid to Range Expansion of the Javan Rhinoceros (Rhinoceros Sondaicus) in Ujung Kulon National Park, Indonesia. J. Wildl. Dis..

[331] Kock R.A., Orynbayev M., Robinson S., Zuther S., Singh N.J., Beauvais W., Morgan E.R., Kerimbayev A., Khomenko S., Martineau H.M. (2018). Saigas on the Brink: Multidisci-Plinary Analysis of the Factors Influencing Mass Mortality Events. Sci. Adv..

[332] Tankaew P., Singh-La T., Titaram C., Punyapornwittaya V., Vongchan P., Sawada T., Sthitmatee N. (2017). Evaluation of an In-House Indirect ELISA for Detection of Antibody against Haemorrhagic Septicemia in Asian Elephants. J. Microbiol. Methods.

[333] Marza A.D., Abdullah F.F.J., Ahmed I.M., Chung E.L., Ibrahim H.H., Zamri-Saad M., Omar A.R., Bakar M.Z., Sa-haree A.A., Haron A. (2015). Involvement of Nervous System in Cattle and Buffaloes Due to Pasteurella Multocida B:2 Infection: A Review of Clinicopathological and Pathophysiological Changes. J. Adv. Vet. Anim. Res..

[334] Jilo K., Belachew T., Birhanu W., Habte D., Yadeta W., Giro A. (2020). Pasteurellosis Status in Ethiopia: A Comprehensive Review. J. Trop. Dis. Public Health.

[335] Fereidouni S., Freimanis G.L., Orynbayev M., Ribeca P., Flannery J., King D.P., Zuther S., Beer M., Hoper D., Kydyrmanov A. (2019). Mass Die-Off. of Saiga Antelopes, Kazakhstan, 2015. Emerg. Infect. Dis..

[336] Robinson S., Milner-Gulland E.J., Grachev Y., Salemgareyev A., Orynbayev M., Lushchekina A., Morgan E., Beauvais W., Singh N., Khomenko S. (2019). Opportunistic Bacteria and Mass Mortality in Ungulates: Lessons from an Extreme Event. Ecosphere.

[337] Khan A., Saleemi M.K., Khan M.Z., Gul S.T., Irfan M., Qamar M.S. (2011). Hemorrhagic Septicemia in Buffalo (Bubalus bubalis) Calves under Sub-Tropical Conditions in Pakistan. Pak. J. Zool..

[338] Townsend K.M., Frost A.J., Lee C.W., Papadimitriou J.M., Dawkins H.J. (1998). Development of PCR Assays for Species- and Type-Specific Identification of Pasteurella Multocida Isolates. J. Clin. Microbiol..

[339] Moustafa A.M., Bennett M.D. (2017). Development of Loop-Mediated Isothermal Amplification-Based Diagnostic Assays for Detection of Pasteurella Multocida and Hemorrhagic Septicemia-Associated P Multocida Serotype B:2. Am. J. Vet. Res..

[340] Zhao G., He H., Wang H. (2019). Use of a Recombinase Polymerase Amplification Commercial Kit for Rapid Visual Detection of Pasteurella Multocida. BMC Vet. Res..

[341] Bote Y., Legesse K., Tassew A., Zeit E.D. (2017). Isolation, Identification and Antimicrobial Susceptibility of Pasteurella Multocida from Cattle with Hemorrhagic Septicemia in Assosa and Bambasi Districts, Benishangul Gumuz Regional State, Ethiopia. Int. J. Anim. Res..

[342] Cuevas I., Carbonero A., Cano-Terriza D., García-Bocanegra I., Amaro M.Á., Borge C. (2020). Antimicrobial Resistance of Pasteurella Multocida Type B Isolates Associated with Acute Septicemia in Pigs and Cattle in Spain. BMC Vet. Res..

[343] Mostaan S., Ghasemzadeh A., Sardari S., Shokrgozar M.A., Brujeni G.N., Abolhassani M., Ehsani P., Karam M.R.A. (2020). Pasteurella Multocida Vaccine Candidates: A Systematic Review. Avicenna J. Med. Biotechnol..

[344] Verma R., Jaiswal T. (1998). Haemorrhagic Septicaemia Vaccines. Vaccine.

[345] Hussain Shah A., Kamboh A.A., Rajput N., Korejo N.A. (2008). Optimization of Physico-chemical Conditions for the Growth of Pasteurella multocida under In Vitro. J. Agric. Soc. Sci..

[346] Varshney R., Varshney R., Chaturvedi V.K., Rawat M., Saminathan M., Singh V., Singh R., Sahoo M., Gupta P.K. (2020). De-Velopment of Novel Iron-Regulated Pasteurella Multocida B: 2 Bacterin and Refinement of Vaccine Quality in Terms of Minimum Variation in Particle Size and Distribution Vis-a-Vis Critical Level of Iron in Media. Microb. Pathog..

[347] Gulia D., Aly S.S. (2020). Modeling Vaccination Programs in Outbreaks of Hemorrhagic Septicemia in India. J. Anim. Res..

[348] Yap S.K., Zakaria Z., Othman S.S., Omar A.R. (2018). In Vitro Treatment of Lipopolysaccharide Increases Invasion of Pasteurella Mul-Tocida Serotype B:2 into Bovine Aortic Endothelial Cells. J. Vet. Sci..

[349] Puspitasari Y., Annas S., Adza-Rina M.N., Zamri-Saad M. (2019). In-Vitro Phagocytosis and Intracellular Killing of Pasteurella Mul-Tocida B:2 by Phagocytic Cells of Buffaloes. Microb. Pathog..

[350] Puspitasari Y., Salleh A., Zamri-Saad M. (2020). Ultrastructural Changes in Endothelial Cells of Buffaloes Following In-Vitro Exposure to Pasteurella Multocida B:2. BMC Vet. Res..

[351] Shah N., Biewenga J., De Graaf F.K. (1996). Vacuolating Cytotoxic Activity of Pasteurella Multocida Causing Haemorrhagic Septicaemia in Buffalo and Cattle. FEMS Microbiol. Lett..

[352] Sawada T., Rimler R.B., Rhoades K.R. (1985). Hemorrhagic Septicemia: Naturally Acquired Antibodies against Pasteurella Multocida Types B and E in Calves in the United States. Am. J. Vet. Res..

[353] Yasin I.-S.M., Yusoff S.M., Mohd Z.-S., Mohd E.A.W. (2010). Efficacy of an Inactivated Recombinant Vaccine Encoding a Fimbrial Protein of Pasteurella Multocida B:2 against Hemorrhagic Septicemia in Goats. Trop. Anim. Health Prod..

[354] Zamri-Saad M., Ernie Z.A., Sabri M.Y. (2006). Protective Effect Following Intranasal Exposure of Goats to Live Pasteurella Multocida B:2. Trop. Anim. Health Prod..

[355] Kharb S., Charan S. (2012). Mouse Model of Haemorrhagic Septicaemia: Dissemination and Multiplication of Pasteurella Multocida B:2 in Vital Organs after Intranasal and Subcutaneous Challenge in Mice. Vet. Res. Commun..

[356] Kumar B., Chaturvedi V.K., Somrajan S.R., Kumar P., Sreedevi R., Kumar S., Kaushik P. (2011). Comparative Immune Response of Purified Native Omph Protein Derived from Pasteurella Multocida P52 and Oil Adjuvant Vaccine against Hemorrhagic Septicemia in Mice. Indian J. Anim. Sci..

[357] Qu Y., Qiu Z., Cao C., Lu Y., Sun M., Liang C., Zeng Z. (2015). Pharmacokinetics/Pharmacodynamics of Marbofloxacin in a Pasteurella Multocida Serious Murine Lung Infection Model. BMC Vet. Res..

[358] Qureshi S., Saxena H.M. (2019). Efficacy of bacteriophage Lysed Pasteurella Marker Vaccine in Laboratory Animal Models with a Novel DIVA for Haemorrhagic Septicaemia. Saudi J. Biol. Sci..

[359] Nimtrakul P., Atthi R., Limpeanchob N., Tiyaboonchai W. (2013). Development of Pasteurella Multocida-Loaded Microparticles for Hemorrhagic Septicemia Vaccine. Drug Dev. Ind. Pharm..

[360] Ramdani H.J.S.D., Johnson R.B., Spencer T.L., Adler B., Dawkins H. (1990). Pasteurella Multocida Infections in Mice with Reference to Haemorrhagic Septicaemia in Cattle snd Buffalo. Immunol. Cell Biol..

[361] Shivachandra S.B., Yogisharadhya R., Kumar A., Mohanty N.N., Nagaleekar V.K. (2015). Recombinant Transferrin Binding Protein A (Rtbpa) Fragments of Pasteurella Multocida Serogroup B:2 Provide Variable Protection Following Homologous Challenge in Mouse Model. Res. Vet. Sci..

[362] Singh M., Verma R., Pandit R.S., Tarfain N.U. (2017). Host Responses and Bacterial Colonization Following Inoculation of Pasteurella Multocida P52 Strain into Unvaccinated and Aluminum Hydroxide Gel Hemorrhagic Septicemia Vaccinated Mice. Microb. Pathog..

[363] Tabatabaei M., Liu Z., Finucane A., Parton R., Coote J. (2002). Protective Immunity Conferred by Attenuated aroA Derivatives of Pasteurella multocida B:2 Strains in a Mouse Model of Hemorrhagic Septicemia. Infect. Immun..

[364] Chelliah S., Velappan R.D., Lim K.T., Swee C.W.K., Rashid N.N., Rothan H.A., Kabir N., Ismail S. (2020). Potential DNA Vaccine for Haemorrhagic Septiceamia Disease. Mol. Biotechnol..

[365] Hussaini J., Nazmul M.H.M., Masyitah N., Abdullah M.A., Ismail S. (2013). Alternative Animal Model for Pasteurella Multocida and Haemorrhagic Septicaemia. Biomed. Res. India.

[366] Kang T.L., Velappan R.D., Kabir N., Mohamad J., Rashid N.N., Ismail S. (2019). The ABA392/pET30a Protein of Pasteurella Mul-Tocida Provoked Mucosal Immunity against HS Disease in a Rat Model. Microb. Pathog..

[367] Annas S., Abubakar M.S., Zamri-Saad M., Jesse F.F.A., Zunita Z. (2015). Pathological Changes in the Respiratory, Gastrointestinal and Urinary Tracts of Buffalo Calves Following Experimental Hemorrhagic Septicemia. Pak. Vet. J..

[368] Hodgson J.C., Finucane A., Dagleish M.P., Ataei S., Parton R., Coote J.G. (2005). Efficacy of Vaccination of Calves against Hemorrhagic Septicemia with a Live aroA Derivative of Pasteurella multocida B:2 by Two Different Routes of Administration. Infect. Immun..

[369] Muangthai K., Tankaew P., Varinrak T., Uthi R., Rojanasthien S., Sawada T., Sthitmatee N. (2018). Intranasal Immunization with a Recombinant Outer Membrane Protein H Based Haemorrhagic Septicemia Vaccine in Dairy Calves. J. Vet. Med Sci..

[370] Muenthaisong A., Nambooppha B., Rittipornlertrak A., Tankaew P., Varinrak T., Muangthai K., Atthikanyaphak K., Sawada T., Sthitmatee N. (2020). An Intranasal Vaccination with a Recombinant Outer Membrane Protein H against Haemorrhagic Sep-ticemia in Swamp Buffaloes. Vet. Med. Int..

[371] Tabatabaei M., Jula G.R.M., Jabbari A.R., Esmailzadeh M. (2007). Vaccine Efficacy in Cattle Against Hemorrhagic Septicemia with Live Attenuated Aroa Mutant of Pasteurella Multocida B:2 Strain. J. Cell Anim. Biol..

[372] Namazi F., Tafti A.K. (2021). Lumpy Skin Disease, an Emerging Transboundary Viral Disease: A Review. Vet. Med. Sci..

[373] Hunter P., Wallace D. (2001). Lumpy Skin Disease in Southern Africa: A Review of the Disease and Aspects of Control. J. S. Afr. Vet. Assoc..

[374] Lu G., Xie J., Luo J., Shao R., Jia K., Li S. (2021). Lumpy Skin Disease Outbreaks in China, since 3 August 2019. Transbound. Emerg. Dis..

[375] Beard P.M. (2016). Lumpy Skin Disease: A Direct Threat to Europe. Vet. Rec..

[376] Tuppurainen E., Alexandrov T., Beltran-Alcrudo D. (2017). Lumpy Skin Disease Field Manual—A Manual for Veterinarians. FAO Animal Production and Health Manual No. 20. Rome.

[377] Mulatu E., Feyisa A. (2018). Review: Lumpy Skin Disease. J. Vet. Sci. Technol..

[378] Davies F.G. (1991). Lumpy Skin Disease, an African Capripox Virus Disease of Cattle. Br. Vet. J..

[379] Kumar S.M. (2011). An Outbreak of Lumpy Skin Disease in a Holstein Dairy Herd in Oman: A Clinical Report. Asian J. Anim. Vet. Adv..

[380] Tageldin M.H., Wallace D.B., Gerdes G.H., Putterill J.F., Greyling R.R., Phosiwa M.N., Al Busaidy R.M., Al Ismaaily S.I. (2014). Lumpy Skin Disease of Cattle: An Emerging Problem in the Sultanate of Oman. Trop. Anim. Health Prod..

[381] Sprygin A., Pestova Y., Wallace D., Tuppurainen E., Kononov A. (2019). Transmission of Lumpy Skin Disease Virus: A Short Review. Virus Res..

[382] Calistri P., De Clercq K., Gubbins S., Klement E., Stegeman A., Abrahantes J.C., Marojevic D., Antoniou S., Broglia A. (2020). Lumpy Skin Disease Epidemiological Report IV: Data Collection and Analysis. EFSA J..

[383] Tuppurainen E., Venter E., Coetzer J., Bell-Sakyi L. (2015). Lumpy Skin Disease: Attempted Propagation in Tick Cell Lines and Presence of Viral DNA in Field Ticks Collected from Naturally-Infected Cattle. Ticks Tick Borne Dis..

[384] Sohier C., Haegeman A., Mostin L., De Leeuw I., Van Campe W., De Vleeschauwer A., Tuppurainen E.S.M., Berg T.V.D., De Regge N., De Clercq K. (2019). Experimental Evidence of Mechanical Lumpy Skin Disease Virus Transmission by Stomoxys Calcitrans Biting Flies and *Haematopota* spp. Horseflies. Sci. Rep..

[385] Lubinga J.C., Tuppurainen E.S.M., Mahlare R., Coetzer J.A.W., Stoltsz W.H., Venter E.H. (2013). Evidence of Transstadial and Mechanical Transmission of Lumpy Skin Disease Virus byAmblyomma hebraeumTicks. Transbound. Emerg. Dis..

[386] Lubinga J.C., Tuppurainen E.S.M., Coetzer J.A.W., Stoltsz W.H., Venter E.H. (2014). Transovarial Passage and Transmission of LSDV by Amblyomma Hebraeum, Rhipicephalus Appendiculatus and Rhipicephalus Decoloratus. Exp. Appl. Acarol..

[387] Lubinga J.C., Tuppurainen E.S.M., Coetzer J.A.W., Stoltsz W.H., Venter E.H. (2013). Evidence of Lumpy Skin Disease Virus Over-Wintering by Transstadial Persistence in Amblyomma Hebraeum and Transovarial Persistence in Rhipicephalus Decoloratus Ticks. Exp. Appl. Acarol..

[388] Aleksandr K., Olga B., David W.B., Pavel P., Yana P., Svetlana K., Alexander N., Vladimir R., Dmitriy L., Alexander S. (2020). Non-Vector-Borne Transmission of Lumpy Skin Disease Virus. Sci. Rep..

[389] Irons P., Tuppurainen E., Venter E. (2005). Excretion of Lumpy Skin Disease Virus in Bull Semen. Theriogenology.

[390] Kononov A., Prutnikov P., Shumilova I., Kononova S., Nesterov A., Byadovskaya O., Pestova Y., Diev V., Sprygin A. (2019). Determination of Lumpy Skin Disease Virus in Bovine Meat and Offal Products Following Experimental Infection. Transbound. Emerg. Dis..

[391] Annandale C., Cornelius H., Holm D.E., Ebersohn K., Venter E.H. (2013). Seminal Transmission of Lumpy Skin Disease Virus in Heifers. Transbound. Emerg. Dis..

[392] Gari G., Waret-Szkuta A., Grosbois V., Jacquiet P., Roger F. (2010). Risk Factors Associated with Observed Clinical Lumpy Skin Disease in Ethiopia. Epidemiol. Infect..

[393] Abdulqa H.Y., Rahman H.S., Dyary H.O., Othman H.H. (2016). Lumpy Skin Disease. Reprod. Immunol. Open Access.

[394] Haegeman A., De Vleeschauwer A., De Leeuw I., Vidanović D., Šekler M., Petrović T., Demarez C., Lefebvre D., De Clercq K. (2020). Overview of Diagnostic Tools for Capripox Virus Infections. Prev. Vet. Med..

[395] Hamdi J., Boumart Z., Daouam S., El Arkam A., Bamouh Z., Jazouli M., Tadlaoui K.O., Fihri O.F., Gavrilov B., El Harrak M. (2020). Development and Evaluation of an Inactivated Lumpy Skin Disease Vaccine for Cattle. Vet. Microbiol..

[396] Haegeman A., De Leeuw I., Mostin L., Campe W., Aerts L., Venter E., Tuppurainen E., Saegerman C., De Clercq K. (2021). Comparative Evaluation of Lumpy Skin Disease Virus-Based Live Attenuated Vaccines. Vaccines.

[397] Alkhamis M.A., VanderWaal K. (2016). Spatial and Temporal Epidemiology of Lumpy Skin Disease in the Middle East, 2012–2015. Front. Vet. Sci..

[398] European Food Safety (2018). Lumpy Skin Disease II. Data Collection and Analysis. EFSA J..

[399] Gubbins S., Stegeman A., Klement E., Pite L., Broglia A., Abrahantes J.C. (2020). Inferences about the Transmission of Lumpy Skin Disease Virus between Herds from Outbreaks in Albania in 2016. Prev. Vet. Med..

[400] Kahana-Sutin E., Klement E., Lensky I., Gottlieb Y. (2016). High Relative Abundance of the Stable Fly Stomoxys Calcitrans Is Associated with Lumpy Skin Disease Outbreaks in Israeli Dairy Farms. Med. Vet. Entomol..

[401] Saegerman C., Bertagnoli S., Meyer G., Ganière J.-P., Caufour P., De Clercq K., Jacquiet P., Fournié G., Hautefeuille C., Etore F. (2018). Risk of Introduction of Lumpy Skin Disease in France by the Import of Vectors in Animal Trucks. PLoS ONE.

[402] Saegerman C., Bertagnoli S., Meyer G., Ganière J.-P., Caufour P., De Clercq K., Jacquiet P., Hautefeuille C., Etore F., Casal J. (2018). Risk of introduction of Lumpy Skin Disease into France through Imports of Cattle. Transbound. Emerg. Dis..

[403] Machado G., Korennoy F., Alvarez J., Picasso-Risso C., Perez A., VanderWaal K. (2019). Mapping Changes in the Spatiotemporal Distribution of Lumpy Skin Disease Virus. Transbound. Emerg. Dis..

[404] Allepuz A., Casal J., Beltrán-Alcrudo D. (2018). Spatial Analysis of Lumpy Skin Disease in Eurasia—Predicting Areas at Risk for Further Spread within the Region. Transbound. Emerg. Dis..

[405] Magori-Cohen R., Louzoun Y., Herziger Y., Oron E., Arazi A., Tuppurainen E., Shpigel N.Y., Klement E. (2012). Mathematical Modelling and Evaluation of the Different Routes of Transmission of Lumpy Skin Disease Virus. Vet. Res..

[406] Ardestani E.G., Mokhtari A. (2020). Modeling the Lumpy Skin Disease Risk Probability in Central Zagros Mountains of Iran. Prev. Vet. Med..

[407] Kononova S., Kononov A., Shumilova I., Byadovskaya O., Nesterov A., Prutnikov P., Babiuk S., Sprygin A. (2021). A Lumpy Skin Disease Virus which Underwent a Recombination Event Demonstrates More Aggressive Growth in Primary Cells and Cattle than the Classical Field Isolate. Transbound. Emerg. Dis..

[408] Wolff J., Krstevski K., Beer M., Hoffmann B. (2020). Minimum Infective Dose of a Lumpy Skin Disease Virus Field Strain from North Macedonia. Viruses.

[409] Sanz-Bernardo B., Haga I.R., Wijesiriwardana N., Hawes P.C., Simpson J., Morrison L.R., MacIntyre N., Brocchi E., Atkinson J., Haegeman A. (2020). Lumpy Skin Disease Is Characterized by Severe Multifocal Dermatitis with Necrotizing Fibrinoid Vasculitis Following Experimental Infection. Vet. Pathol..

[410] Munyanduki H., Douglass N., Offerman K., Carulei O., Williamson A.-L. (2020). Influence of the Lumpy Skin Disease Virus (LSDV) Superoxide Dismutase Homologue on Host Transcriptional Activity, Apoptosis and Histopathology. J. Gen. Virol..

[411] Van Boheemen S., de Graaf M., Lauber C., Bestebroer T.M., Raj V.S., Zaki A.M., Osterhaus A.D.M.E., Haagmans B.L., Gorbalenya A.E., Snijder E.J. (2012). Genomic Characterization of a Newly Discovered Coronavirus Associated with Acute Respiratory Distress Syndrome in Humans. mBio.

[412] Zaki A., Van Boheemen S., Bestebroer T., Osterhaus A., Fouchier R. (2012). Isolation of a Novel Coronavirus from a Man with Pneumonia in Saudi Arabia. N. Engl. J. Med..

[413] Ramadan N., Shaib H. (2019). Middle East. Respiratory Syndrome Coronavirus (MERS-CoV): A Review. Germs.

[414] Widagdo W., Okba N., Raj V.S., Haagmans B.L. (2017). MERS-Coronavirus: From Discovery to Intervention. One Health.

[415] Azhar E.I., El-Kafrawy S.A., Farraj S.A., Hassan A.M., Al-Saeed M.S., Hashem A.M., Madani T.A. (2014). Evidence for Cam-el-to-Human Transmission of MERS Coronavirus. N. Engl. J. Med..

[416] Dighe A., Jombart T., van Kerkhove M.D., Ferguson N. (2019). A Systematic Review of MERS-Cov Seroprevalence and RNA Preva-Lence in Dromedary Camels: Implications for Animal Vaccination. Epidemics.

[417] Haagmans B.L., van den Brand J.M.A., Raj V.S., Volz A., Wohlsein P., Smits S.L., Schipper D., Bestebroer T.M., Okba N., Fux R. (2016). An Orthopoxvirus-Based Vaccine Reduces Virus Excretion After MERS-Cov Infection in Dromedary Camels. Science.

[418] Munster V., De Wit E., Feldmann H. (2013). Pneumonia from Human Coronavirus in a Macaque Model. N. Engl. J. Med..

[419] Yao Y., Bao L., Deng W., Xu L., Li F., Lv Q., Yu P., Chen T., Xu Y., Zhu H. (2014). An Animal Model of MERS Produced by Infection of Rhesus Macaques with MERS Coronavirus. J. Infect. Dis..

[420] De Wit E., Prescott J., Baseler L., Bushmaker T., Thomas T., Lackemeyer M.G., Martellaro C., Milne-Price S., Haddock E., Haagmans B.L. (2013). The Middle East. Respiratory Syndrome Coronavirus (MERS-CoV) Does Not. Replicate in Syrian Hamsters. PLoS ONE.

[421] Jaiswal N.K., Saxena S.K., Saxena S.K. (2020). Classical Coronaviruses. Coronavirus Disease 2019 (COVID-19): Epidemiology, Pathogenesis, Diagnosis, and Therapeutics.

[422] Liya G., Yuguang W., Jian L., Huaiping Y., Xue H., Jianwei H., Jiaju M., Youran L., Chen M., Yiqing J. (2020). Studies on Viral Pneumonia Related to Novel Coronavirus SARS-CoV-2, SARS-CoV, and MERS-CoV: A Literature Review. APMIS.

[423] Xie M., Chen Q. (2020). Insight into 2019 Novel Coronavirus—An Updated Interim Review and Lessons from SARS-CoV and MERS-CoV. Int. J. Infect. Dis..

[424] Yan Y., Chang L., Wang L. (2020). Laboratory Testing of SARS-CoV, MERS-CoV, and SARS-CoV-2 (2019-nCoV): Current Status, Challenges, and Countermeasures. Rev. Med. Virol..

[425] Alexander D.J., Bell J.G., Alders R.G. (2004). A Technology Review: Newcastle Disease—With Special Emphasis on Its Effects on Village Chickens.

[426] Alexander D.J. (2000). Newcastle Disease and Other Avian Paramyxovirus. Rev. Sci. Tech..

[427] Alexander D.J. (2001). Gordon Memorial Lecture. Newcastle Disease. Br. Poult. Sci..

[428] Bello M.B., Yusoff K.M., Ideris A., Hair-Bejo M., Peeters B.P.H., Jibril A.H., Tambuwal F.M., Omar A.R. (2018). Genotype Diversity of Newcastle Disease Virus in Nigeria: Disease Control Challenges and Future Outlook. Adv. Virol..

[429] Alexander D.J. (2009). Ecology and Epidemiology of Newcastle Disease.

[430] Jadhav A., Zhao L., Ledda A., Liu W., Ding C., Nair V., Ferretti L. (2020). Patterns of RNA Editing in Newcastle Disease Virus Infections. Viruses.

[431] Mayers J., Mansfield K.L., Brown I.H. (2017). The Role of Vaccination in Risk Mitigation and Control of Newcastle Disease in Poultry. Vaccine.

[432] Dimitrov K.M., Ramey A., Qiu X., Bahl J., Afonso C.L. (2016). Temporal, Geographic, and Host Distribution of Avian Paramyxovirus 1 (Newcastle Disease Virus). Infect. Genet. Evol..

[433] Cox R.M., Plemper R.K. (2017). Structure and Organization of Paramyxovirus Particles. Curr. Opin. Virol..

[434] Nooruzzaman M., Mumu T.T., Kabiraj C.K., Hasnat A., Rahman M.M., Chowdhury E.H., Dimitrov K.M., Islam M.R. (2021). Genetic and Biological Characterization of Newcastle Disease Viruses Circulating in Bangladesh During 2010–2017: Further Genetic Di-Versification of Class II Genotype XIII in Southcentral Asia. J. Gen. Virol..

[435] Alamian A., A Pourbakhsh S., Shoushtari A., Keivanfar H. (2019). Seroprevalence Investigation of Newcastle Disease in Rural Poultries of the Northern Provinces (Golestan, Gilan, and Mazandaran) of Iran. Arch. Razi. Inst..

[436] Alsahami A., Ideris A., Omar A., Ramanoon S.Z., Sadiq M.B. (2018). Seroprevalence of Newcastle Disease Virus in Backyard Chickens and Herd-Level Risk Factors of Newcastle Disease in Poultry Farms in Oman. Int. J. Vet. Sci. Med..

[437] Nwanta J., Abdu P., Ezema W. (2008). Epidemiology, Challenges and Prospects for Control of Newcastle Disease in Village Poultry in Nigeria. World Poult. Sci. J..

[438] Terfa Z., Garikipati S., Kassie G., Bettridge J., Christley R. (2018). Eliciting Preferences for Attributes of Newcastle Disease Vaccination Programmes for Village Poultry in Ethiopia. Prev. Vet. Med..

[439] De Bruyn J., Thomson P.C., Bagnol B., Maulaga W., Rukambile E., Alders R.G. (2017). The Chicken or the Egg? Exploring Bi-Directional Associations between Newcastle Disease Vaccination and Village Chicken Flock Size in Rural Tanzania. PLoS ONE.

[440] Ipara B.O., Otieno D.J., Nyikal R., Makokha N.S. (2021). The Contribution of Extensive Chicken Production Systems and Practices to Newcastle Disease Outbreaks in Kenya. Trop. Anim. Health Prod..

[441] Hugo A., Makinde O.D., Kumar S., Chibwana F.F. (2016). Optimal Control and Cost Effectiveness Analysis for Newcastle Disease Eco-Epidemiological Model in Tanzania. J. Biol. Dyn..

[442] Habibi H., Firuzi S., Nili H., Asasi K., Mosleh N. (2020). Efficacy of Thermostable Newcastle Disease Virus Strain I-2 in Broiler Chickens Challenged with Highly Virulent Newcastle Virus. Arch. Razi. Inst..

[443] Taylor T.L., Miller P.J., Olivier T.L., Montiel E., Garcia S.C., Dimitrov K.M., Williams-Coplin D., Afonso C.L. (2017). Repeated Challenge with Virulent Newcastle Disease Virus Does Not Decrease the Efficacy of Vaccines. Avian Dis..

[444] Osman N., Goovaerts D., Sultan S., Salt J., Grund C. (2021). Vaccine Quality Is a Key Factor to Determine Thermal Stability of Commercial Newcastle Disease (ND)Vaccines. Vaccines.

[445] Mahmood M.S., Sabir R. (2021). Preparation and Evaluation of Avian Influenza (H9) and Newcastle Disease (Thermostable I-2 Strain) Bivalent Vaccine for Commercial Poultry. Agrobiol. Rec..

[446] Zhao Y., Liu H., Cong F., Wu W., Zhao R., Kong X. (2018). Phosphoprotein Contributes to the Thermostability of Newcastle Disease Virus. BioMed Res. Int..

[447] Peebles E. (2018). In ovo Applications on Poultry: A review. Poult. Sci..

[448] Ike A.C., Ononugbo C.M., Obi O.J., Onu C.J., Olovo C.V., Muo S.O., Chukwu O.S., Reward E.E., Omeke O.P. (2021). Towards Improved Use of Vaccination in the Control of Infectious Bronchitis and Newcastle Disease in Poultry: Understanding the Immunological Mechanisms. Vaccines.

[449] Cvetić Ž., Nedeljković G., Jergović M., Bendelja K., Mazija H., Gottstein Ž. (2021). Immunogenicity of Newcastle Disease Virus Strain ZG1999HDS Applied Oculonasally or by Means of Nebulization to Day-Old Chicks. Poult. Sci..

[450] Shittu I., Zhu Z., Lu Y., Hutcheson J.M., Stice S.L., West F.D., Donadeu M., Dungu B., Fadly A.M., Zavala G. (2016). Development, Characterization and Optimization of a New Suspension Chicken-Induced Pluripotent Cell Line for the Production of Newcastle Disease Vaccine. Biologicals.

[451] Shahar E., Haddas R., Goldenberg D., Lublin A., Bloch I., Hinenzon N.B., Pitcovski J. (2018). Newcastle Disease Virus: Is an Updated Attenuated Vaccine Needed?. Avian Pathol..

[452] Ferreira H., Miller P., Suarez D. (2021). Protection against Different Genotypes of Newcastle Disease Viruses (NDV) Afforded by an Adenovirus-Vectored Fusion Protein and Live NDV Vaccines in Chickens. Vaccines.

[453] Aldous E., Alexander D. (2008). Newcastle Disease in Pheasants (Phasianus Colchicus): A Review. Vet. J..

[454] Alexander D.J., Alexander D.J. (2000). Newcastle Disease in Ostriches (Struthio Camelus)—A Review. Avian Pathol..

[455] Kuiken T., Frandsen D., Clavijo A. (1998). Newcastle Disease in Cormorants. Can. Vet. J..

[456] Samour J. (2014). Newcastle Disease in Captive Falcons in the Middle East: A Review of Clinical and Pathologic Findings. J. Avian Med. Surg..

[457] Brown V.R., Bevins S.N. (2017). A Review of Virulent Newcastle Disease Viruses in the United States and the Role of Wild Birds in Viral Persistence and Spread. Vet. Res..

[458] Zhao P., Sun L., Sun X., Li S., Zhang W., Pulscher L.A., Chai H., Xing M. (2017). Newcastle Disease Virus from Domestic Mink, China, 2014. Vet. Microbiol..

[459] Elmberg J., Berg C., Lerner H., Waldenström J., Hessel R. (2017). Potential Disease Transmission from Wild Geese and Swans to Livestock, Poultry and Humans: A Review of the Scientific Literature from a One Health Perspective. Infect. Ecol. Epidemiol..

[460] Rehan M., Aslam A., Khan M.-U.-R., Abid M., Hussain S., Umber J., Anjum A., Hussain A. (2019). Potential Economic Impact of Newcastle Disease Virus Isolated from Wild Birds on Commercial Poultry Industry of Pakistan: A Review. Hosts Viruses.

[461] Bello M.B., Yusoff K., Ideris A., Hair-Bejo M., Jibril A.H., Peeters B.P.H., Omar A.R. (2020). Exploring the Prospects of Engineered Newcastle Disease Virus in Modern Vaccinology. Viruses.

[462] Lundstrom K. (2019). RNA Viruses as Tools in Gene Therapy and Vaccine Development. Genes.

[463] Vijayakumar G., McCroskery S., Palese P. (2020). Engineering Newcastle Disease Virus as an Oncolytic Vector for Intratumoral De-livery of Immune Checkpoint Inhibitors and Immunocytokines. J. Virol..

[464] Choi K.-S. (2017). Newcastle Disease Virus Vectored Vaccines as Bivalent or Antigen Delivery Vaccines. Clin. Exp. Vaccine Res..

[465] Chen X., Yang J., Ji Y., Okoth E., Liu B., Li X., Yin H., Zhu Q. (2016). Recombinant Newcastle Disease Virus Expressing African Swine Fever Virus Protein 72 Is Safe and Immunogenic in Mice. Virol. Sin..

[466] Ch’Ng W.-C., Stanbridge E.J., Ong K.-C., Wong K.-T., Yusoff K., Shafee N. (2011). Partial Protection against Enterovirus 71 (EV71) Infection in a Mouse Model Immunized with Recombinant Newcastle Disease Virus Capsids Displaying the EV71 VP1 Fragment. J. Med. Virol..

[467] Debnath A., Pathak D.C., D’Silva A.L., Batheja R., Ramamurthy N., Vakharia V.N., Chellappa M.M., Dey S. (2020). Newcastle Disease Virus Vectored Rabies Vaccine Induces Strong Humoral and Cell Mediated Immune Responses in Mice. Vet. Microbiol..

[468] Manoharan V.K., Khattar S.K., LaBranche C.C., Montefiori D.C., Samal S.K. (2018). Modified Newcastle Disease Virus as an Im-Proved Vaccine Vector against Simian Immunodeficiency Virus. Sci. Rep..

[469] Shirvani E., Samal S.K. (2020). Newcastle Disease Virus as a Vaccine Vector for SARS-CoV-2. Pathogens.

[470] Sun W., McCroskery S., Liu W.C., Leist S.R., Liu Y., Albrecht R.A., Slamanig S., Oliva J., Amanat F., Schäfer A. (2020). A Newcastle Disease Virus (NDV) Expressing a Mem-brane-Anchored Spike as a Cost-Effective Inactivated SARS-CoV-2 Vaccine. Vaccines.

[471] Yang Y., Shi W., Abiona O.M., Nazzari A., Olia A.S., Ou L., Phung E., Stephens T., Tsybovsky Y., Verardi R. (2021). Newcastle Disease Virus-Like Particles Displaying Prefusion-Stabilized SARS-CoV-2 Spikes Elicit Potent Neutralizing Responses. Vaccines.

[472] Schirrmacher V. (2017). Immunobiology of Newcastle Disease Virus and Its Use for Prophylactic Vaccination in Poultry and as Adjuvant for Therapeutic Vaccination in Cancer Patients. Int. J. Mol. Sci..

[473] Vijayakumar G., Zamarin D. (2019). Design and Production of Newcastle Disease Virus for Intratumoral Immunomodulation. Methods Mol. Biol..

[474] Burman B., Pesci G., Zamarin D. (2020). Newcastle Disease Virus at the Forefront of Cancer Immunotherapy. Cancers.

[475] Meng Q., He J., Zhong L., Zhao Y. (2021). Advances in the Study of Antitumour Immunotherapy for Newcastle Disease Virus. Int. J. Med. Sci..

[476] Yurchenko K.S., Zhou P., Kovner A.V., Zavjalov E.L., Shestopalova L.V., Shestopalov A.M. (2018). Oncolytic Effect of Wild-Type Newcastle Disease Virus Isolates in Cancer Cell Lines In Vitro and In Vivo on Xenograft Model. PLoS ONE.

[477] Amin Z.M., Ani M.A.C., Tan S.W., Yeap S.K., Alitheen N.B., Najmuddin S.U.F.S., Kalyanasundram J., Chan S.C., Veerakumarasivam A., Chia S.L. (2019). Evaluation of a Recombinant Newcastle Disease Virus Expressing Human IL12 against Human Breast Cancer. Sci. Rep..

[478] Li X., Chai T., Wang Z., Song C., Cao H., Liu J., Zhang X., Wang W., Yao M., Miao Z. (2009). Occurrence and Transmission of Newcastle Disease Virus Aerosol Originating from Infected Chickens under Experimental Conditions. Vet. Microbiol..

[479] Soliman M.A., Nour A.A., Erfan A.M. (2019). Quantitative Evaluation of Viral Interference among Egyptian Isolates of Highly Pathogenic Avian Influenza Viruses (H5N1 And H5N8) with the Lentogenic and Velogenic Newcastle Disease Virus Genotype VII in Specific Patho-Gen-Free Embryonated Chicken Eggs Model. Vet. World.

[480] Carrasco A.D.O.T., Seki M.C., Benevenute J.L., Ikeda P., Pinto A.A. (2016). Experimental Infection with Brazilian Newcastle Disease Virus Strain in Pigeons and Chickens. Braz. J. Microbiol..

[481] Gallili G.E., Ben-Nathan D. (1998). Newcastle Disease Vaccines. Biotechnol. Adv..

[482] Palya V., Tatár-Kis T., Arafa A.S.A., Felföldi B., Mató T., Setta A. (2021). Efficacy of a Turkey Herpesvirus Vectored Newcastle Disease Vaccine against Genotype VII.1.1 Virus: Challenge Route Affects Shedding Pattern. Vaccines.

[483] Rehman Z., Ren S., Butt S., Manzoor Z., Iqbal J., Anwar M., Sun Y., Qiu X., Tan L., Liao Y. (2021). Newcastle Disease Virus Induced Pathologies Severely Affect the Exocrine and Endocrine Functions of the Pancreas in Chickens. Genes.

[484] Lardinois A., Berg T.V.D., Lambrecht B., Steensels M. (2014). A Model for the Transfer of Passive Immunity against Newcastle Disease and Avian Influenza in Specific Pathogen Free Chickens. Avian Pathol..

[485] Susta L., Segovia D., Olivier T.L., Dimitrov K.M., Shittu I., Marcano V., Miller P.J. (2018). Newcastle Disease Virus Infection in Quail. Vet. Pathol..

[486] Dimitrov K.M., Ferreira H.L., Pantin-Jackwood M.J., Taylor T.L., Goraichuk I.V., Crossley B.M., Killian M.L., Bergeson N.H., Torchetti M.K., Afonso C.L. (2019). Pathogenicity and Transmission of Virulent Newcastle Disease Virus from the 2018–2019 California Outbreak and Related Viruses in Young and Adult Chickens. Virology.

[487] Schilling M.A., Katani R., Memari S., Cavanaugh M., Buza J., Radzio-Basu J., Mpenda F.N., Deist M.S., Lamont S.J., Kapur V. (2018). Transcriptional Innate Immune Response of the Developing Chicken Embryo to Newcastle Disease Virus Infection. Front. Genet..

[488] Miller P.J., Kim L.M., Ip H., Afonso C.L. (2009). Evolutionary Dynamics of Newcastle Disease Virus. Virology.

[489] Abd El-Baky N., Amara A.A. (2014). Newcastle Disease Virus (Lasota Strain) as a Model for Virus Ghosts Preparation Using H2O2 Bio-Critical Concentration. Int. Sci. Investig. J..

[490] Njeumi F., Bailey D., Soula J.J., Diop B., Tekola B.G. (2020). Eradicating the Scourge of Peste des Petits Ruminants from the World. Viruses.

[491] Albina E., Kwiatek O., Minet C., Lancelot R., de Almeida R.S., Libeau G. (2013). Peste des Petits Ruminants, the Next Eradicated Animal Disease?. Vet. Microbiol..

[492] Idoga E.S., Armson B., Alafiatayo R., Ogwuche A., Mijten E., Ekiri A.B., Varga G., Cook A.J.C. (2020). A Review of the Current Status of Peste des Petits Ruminants Epidemiology in Small Ruminants in Tanzania. Front. Vet. Sci..

[493] Baron M.D., Parida S., Oura C.A.L. (2011). Peste des Petits Ruminants: A Suitable Candidate for Eradication?. Vet. Rec..

[494] Kabir A., Kalhoro D.H., Abro S.H., Kalhoro M.S., Yousafzai H.A., Shams S., Khan I.U., Lochi G.M., Mazari M.Q., Baloch M.W. (2019). Peste des Petits Ruminants: A Review. Pure Appl. Biol..

[495] Torsson E., Kgotlele T., Berg M., Mtui-Malamsha N., Swai E.S., Wensman J.J., Misinzo G. (2016). History and Current Status of Peste des Petits Ruminants Virus in Tanzania. Infect. Ecol. Epidemiol..

[496] Ahaduzzaman M. (2020). Peste des Petits Ruminants (PPR) in Africa and Asia: A Systematic Review and Meta-Analysis of the Prevalence in Sheep and Goats between 1969 and 2018. Vet. Med. Sci..

[497] Rahman A.U., Dhama K., Ali Q., Hussain I., Oneeb M., Chaudhary U., Wensman J.J., Shabbir M.Z. (2020). Peste des Petits Ru-Minants in Large Ruminants, Camels and Unusual Hosts. Vet. Q..

[498] Agga G.E., Raboisson D., Walch L., Alemayehu F., Semu D.T., Bahiru G., Woube Y.A., Belihu K., Tekola B.G., Bekana M. (2019). Epidemiological Survey of Peste des Petits Ruminants in Ethiopia: Cattle as Potential Sentinel for Sur-veillance. Front. Vet. Sci..

[499] Rahman A.U., Wensman J.J., Abubakar M., Shabbir M.Z., Rossiter P. (2018). Peste des Petits Ruminants in Wild Ungulates. Trop. Anim. Health Prod..

[500] Schulz C., Fast C., Wernery U., Kinne J., Joseph S., Schlottau K., Jenckel M., Höper D., Patteril N.A.G., Syriac G. (2019). Camelids and Cattle Are Dead-End Hosts for Peste-des-Petits-Ruminants Virus. Viruses.

[501] Nour H.S.H. (2020). Challenges and Opportunities for Global Eradication of Paste des Petits Ruminants (PPR). J. Trop. Dis. Public Health.

[502] Zakian A., Nouri M., Faramarzian F., Tehrani-Sharif M., Rezaie A., Mokhber-Dezfouli M.R. (2016). Comprehensive Review on Peste Des. Petits Ruminants [PPR] Disease in Ruminants and Camels: With Emphasis on Clinical Signs and Histopathological Finding. J. Vet. Sci. Med. Diagn..

[503] Balamurugan V., Hemadri D., Gajendragad M.R., Singh R.K., Rahman H. (2013). Diagnosis and Control of Peste des Petits Ruminants: A Comprehensive Review. Virus Dis..

[504] Giato C.G., SKihu M.H., Bebora L.C., Njenga J.M., Wairire G.G., Maingi N., Muse E.A., Karimuribo E.D., Misinzo G., Mellau L.S.B. Comparison of Peste des Petits Ruminants (PPR) Disease between Tanzania and Kenya. Proceedings of the 3rd RUFORUM Biennial Conference.

[505] Akanbi O.B., Franzke K., Adedeji A.J., Ulrich R., Teifke J.P. (2020). Peste des Petits Ruminants Virus and Goatpox Virus Co-Infection in Goats. Vet. Pathol..

[506] Adedeji A.J., Dashe Y., Akanbi O.B., Woma T.Y., Jambol A.R., Adole J.A., Bolajoko M.B., Chima N., Asala O., Tekki I.S. (2019). Co-Infection of Peste des Petits Ruminants and Goatpox in a Mixed Flock of Sheep and Goats in Kanam, North Central Nigeria. Vet. Med. Sci..

[507] Kinimi E., Odongo S., Muyldermans S., Kock R., Misinzo G. (2020). Paradigm Shift in the Diagnosis of Peste des Petits Ruminants: Scoping Review. Acta Vet. Scand..

[508] Manzoor S., Abubakar M., Bin Zahur A., Yunus A.W., Ullah A., Afzal M. (2020). Genetic Characterization of Peste des Petits Ruminants Virus (Pakistani Isolates) and Comparative Appraisal of Diagnostic Assays. Transbound. Emerg. Dis..

[509] Kardjadj M., Luka P.D. (2016). Molecular Epidemiology of Foot and Mouth Disease, Bluetongue and Pest des Petits Ruminants in Algeria: Historical Perspective, Diagnosis and Control. Afr. J. Biotechnol..

[510] Mahapatra M., Howson E., Fowler V., Batten C., Flannery J., Selvaraj M., Parida S. (2019). Rapid Detection of Peste des Petits Ruminants Virus (PPRV) Nucleic Acid Using a Novel Low-Cost Reverse Transcription Loop-Mediated Isothermal Amplification (RT-LAMP) Assay for Future Use in Nascent PPR Eradication Programme. Viruses.

[511] Baron M., Diallo A., Lancelot R., Libeau G. (2016). Peste des Petits Ruminants Virus. Adv. Clin. Chem..

[512] Edo T., Deneke Y., Abdela N. (2017). Peste Des Petits Ruminants and its Economic Importance. Glob. Vet..

[513] Abubakar M., Irfan M. (2014). An Overview of Treatment Options to Combat Peste des Petits Ruminants in Endemic Situations. Res. J. Vet. Pract..

[514] Baron M.D., Diop B., Njeumi F., Willett B.J., Bailey D. (2017). Future research to underpin successful peste des petits ruminants virus (PPRV) eradication. J. Gen. Virol..

[515] Parida S., Muniraju M., Mahapatra M., Muthuchelvan D., Buczkowski H., Banyard A. (2015). Peste des Petits Ruminants. Vet. Microbiol..

[516] Kumar N., Barua S., Riyesh T., Tripathi B.N. (2017). Advances in Peste des Petits Ruminants Vaccines. Vet. Microbiol..

[517] Jia X.-X., Wang H., Liu Y., Meng D.-M., Fan Z.-C., Hui W. (2020). Development of Vaccines for Prevention of Peste-des-Petits-Ruminants Virus Infection. Microb. Pathog..

[518] Dundon W.G., Diallo A., Cattoli G. (2020). Peste des Petits Ruminants in Africa: A Review of Currently Available Molecular Epide-Miological Data, 2020. Arch. Virol..

[519] Cameron A.R. (2019). Strategies for the Global Eradication of Peste des Petits Ruminants: An Argument for the Use of Guerrilla Rather Than Trench Warfare. Front. Vet. Sci..

[520] Jafari-Gh A., RLaven A., Eila N., Yadi J., Hatami Z., Soleimani P., Jafari-Gh S., Lesko M.M., Sinafar M., Heidari E. (2020). Transboundary and Infectious Diseases of Small Ruminants: Knowledge, Attitude, and Practice of Nomadic and Semi-Nomadic Pastor-Alists in Northern Iran. Small Rumin. Res..

[521] Acosta D., Hendrickx S., McKune S. (2019). The Livestock Vaccine Supply Chain: Why It Matters and How it Can Help Eradicate Peste des Petits Ruminants, Based on Findings in Karamoja, Uganda. Vaccine.

[522] Bardhan D., Kumar S., Anandsekaran G., Chaudhury J.K., Meraj M., Singh R.K., Verma M.R., Kumar D., Kumar P.T.N., Lone S.A. (2017). The Economic Impact of Peste des Petits Ruminants in India. Rev. Sci. Tech..

[523] Ma J., Gao X., Liu B., Chen H., Xiao J., Wang H. (2019). Peste des Petits Ruminants in China: Spatial Risk Analysis. Transbound. Emerg. Dis..

[524] Fournié G., Waret-Szkuta A., Camacho A., Yigezu L.M., Pfeiffer D., Roger F. (2018). A Dynamic Model of Transmission and Elimination of Peste des Petits Ruminants in Ethiopia. Proc. Natl. Acad. Sci. USA.

[525] Assefa A., Tibebu A., Bihon A., Yimana M. (2020). Global Ecological Niche Modelling of Current and Future Distribution of Peste des Petits Ruminants Virus (Pprv) with an Ensemble Modelling Algorithm. Transbound. Emerg. Dis..

[526] Ruget A.-S., Tran A., Waret-Szkuta A., Moutroifi Y.O., Charafouddine O., Cardinale E., Cêtre-Sossah C., Chevalier V. (2019). Spatial Multicriteria Evaluation for Mapping the Risk of Occurrence of Peste des Petits Ruminants in Eastern Africa and the Union of the Comoros. Front. Vet. Sci..

[527] Munduganore D.S., YMundaganore D., Ashokan K.V. (2012). Sequence Analysis of Protein in Peste des. Petits Ruminants: An. In Silico Approach. Int. J. Food Agric. Vet. Sci..

[528] Parida S., Selvaraj M., Gubbins S., Pope R., Banyard A., Mahapatra M. (2019). Quantifying Levels of Peste des Petits Ruminants (PPR) Virus in Excretions from Experimentally Infected Goats and Its Importance for Nascent PPR Eradication Programme. Viruses.

[529] Truong T., Boshra H., Embury-Hyatt C., Nfon C., Gerdts V., Tikoo S., Babiuk L.A., Kara P.D., Chetty T., Mather A. (2014). Peste des Petits Ruminants Virus Tissue Tropism and Pathogenesis in Sheep and Goats following Experimental Infection. PLoS ONE.

[530] Bamouh Z., Fakri F., Jazouli M., Safini N., Tadlaoui K.O., Elharrak M. (2019). Peste des Petits Ruminants Pathogenesis on Experimental Infected Goats by the Moroccan 2015 Isolate. BMC Vet. Res..

[531] Chandran N.D.J., Kumanan K., Venkatesan R.A. (1995). Differentiation of Peste des Petits Ruminants and Rinderpest Viruses by Neutralisation Indices Using Hyperimmune Rinderpest Antiserum. Trop. Anim. Health Prod..

[532] Yan F., Banadyga L., Schiffman Z., Huang P., Li E., Gao Y., Feng N., Xia X., Feng N. (2019). Peste des Petits Ruminants Virus-Like Particles Induce a Potent Humoral and Cellular Immune Response in Goats. Viruses.

[533] Youssef M., Ibrahim H. (2016). Preparation of Hyperimmune Serum against Peste des Petits Ruminants to Be Used in Emergency Cases. Suez Canal Vet. Med. J. SCVMJ.

[534] Adams M.J., Lefkowitz E.J., King A.M.Q., Harrach B., Harrison R.L., Knowles N.J., Kropinski A.M., Krupovic M., Kuhn J.H., Mushegian A.R. (2017). Changes to Taxonomy and the International Code of Virus Classification and Nomenclature Ratified by the International Committee on Taxonomy of Viruses (2017). Arch. Virol..

[535] Ikegami T. (2012). Molecular Biology and Genetic Diversity of Rift Valley Fever Virus. Antivir. Res..

[536] Grobbelaar A.A., Weyer J., Leman P.A., Kemp A., Paweska J.T., Swanepoel R. (2011). Molecular Epidemiology of Rift Valley Fever Virus. Emerg. Infect. Dis..

[537] Nanyingi M.O., Munyua P., Kiama S.G., Muchemi G.M., Thumbi S.M., Bitek A.O., Bett B., Muriithi R.M., Njenga M.K. (2015). A Systematic Review of Rift Valley Fever Epidemiology 1931–2014. Infect. Ecol. Epidemiol..

[538] Anyamba A., Linthicum K.J., Small J., Britch S.C., Pak E., de la Rocque S., Formenty P., Hightower A.W., Breiman R.F., Chretien J.-P. (2010). Prediction, Assessment of the Rift Valley Fever Activity in East. and Southern Africa 2006?2008 and Possible Vector Control. Strategies. J. Am. Soc. Trop. Med. Hyg..

[539] Linthicum K.J. (2007). A Rift Valley Fever Risk Surveillance System for Africa Using Remotely Sensed Data: Potential for Use on Other Continents. Vet. Ital..

[540] Tantely L.M., Boyer S., Fontenille D. (2015). A Review of Mosquitoes Associated with Rift Valley Fever Virus in Madagascar. Am. J. Trop. Med. Hyg..

[541] Pepin M., Bouloy M., Bird B.H., Kemp A., Paweska J. (2010). Rift Valley Fever Virus (Bunyaviridae: Phlebovirus): An Update on Pathogenesis, Molecular Epidemiology, Vectors, Diagnostics and Prevention. Vet. Res..

[542] Clark M.H.A., Warimwe G.M., di Nardo A., Lyons N.A., Gubbins S. (2018). Systematic Literature Review of Rift Valley Fever Virus Seroprevalence in Livestock, Wildlife and Humans in Africa from 1968 to 2016. PLoS Negl. Trop. Dis..

[543] Bird B.H., Ksiazek T.G., Nichol S.T., Maclachlan N.J. (2009). Rift Valley Fever Virus. J. Am. Vet. Med. Assoc..

[544] Odendaal L., Davis A.S., Venter E.H. (2021). Insights into the Pathogenesis of Viral Haemorrhagic Fever Based on Virus Tropism and Tissue Lesions of Natural Rift Valley Fever. Viruses.

[545] Anyangu A.S., Gould L.H., Sharif S.K., Nguku P.M., Omolo J.O., Mutonga D., Rao C.Y., Lederman E.R., Schnabel D., Paweska J.T. (2010). Risk Factors for Severe Rift Valley Fever Infection in Kenya, 2007. Am. Soc. Trop. Med. Hyg..

[546] Lang Y., Li Y., Jasperson D., Henningson J., Lee J., Ma J., Li Y., Duff M., Liu H., Bai D. (2019). Identification and Evaluation of Antivirals for Rift Valley Fever Virus. Vet. Microbiol..

[547] Atkins C., Freiberg A.N. (2017). Recent Advances in the Development of Antiviral Therapeutics for Rift Valley Fever Virus Infection. Future Virol..

[548] Faburay B., LaBeaud A.D., McVey D.S., Wilson W.C., Richt J.A. (2017). Current Status of Rift Valley Fever Vaccine Development. Vaccines.

[549] Ikegami T., Makino S. (2009). Rift Valley Fever Vaccines. Vaccine.

[550] Calvo-Pinilla E., Marín-López A., Moreno S., Lorenzo G., Trigo S.U., Jiménez-Cabello L., Benavides J., Nogales A., Blasco R., Brun A. (2020). A protective bivalent vaccine against Rift Valley fever and bluetongue. npj Vaccines.

[551] Roeder P., Mariner J., Kock R. (2013). Rinderpest: The veterinary perspective on eradication. Philos. Trans. R. Soc. B: Biol. Sci..

[552] Düx A., Lequime S., Patrono L.V., Vrancken B., Boral S., Gogarten J.F., Hilbig A., Horst D., Merkel K., Prepoint B. (2020). Measles virus and rinderpest virus divergence dated to the sixth century BCE. Science.

[553] Roeder P.L., Taylor W.P. (2002). Rinderpest. Vet. Clin. N. Am. Food Anim. Pract..

[554] Tounkara K., Nwankpa N. (2017). Rinderpest Experience. Rev. Sci. Tech..

[555] Njeumi F., Taylor W., Diallo A., Miyagishima K., Pastoret P.-P., Vallat B., Traore M. (2012). The Long Journey: A Brief Review of the Eradication of Rinderpest. Rev. Sci. Tech..

[556] Barrett T., Rossiter P. (1999). Rinderpest: The Disease and Its Impact on Humans and Animals. Adv. Appl. Microbiol..

[557] Vallat F. (2012). An Outbreak in France in the XVIIIth Century: Rinderpest. C. R. Biol..

[558] Roeder P.L. (2011). Rinderpest: The End of Cattle Plague. Prev. Vet. Med..

[559] Carrillo C., Prarat M., Vagnozzi A., Calahan J.D., Smoliga G., Nelson W.M., Rodriguez L.L. (2010). Specific Detection of Rinderpest Virus by Real-Time Reverse Transcription-PCR in Preclinical and Clinical Samples from Experimentally Infected Cattle. J. Clin. Microbiol..

[560] (1754). Letter to Mr Urban. The Gentleman’s Magazine.

[561] Rossiter P.B., James A.D. (1989). An epidemiological Model of Rinderpest. II. Simulations of the Behaviour of Rinderpest Virus in Popula-Tions. Trop. Anim. Health Prod..

[562] Mariner J.C., House J.A., Mebus C.A., Sollod A.E., Chibeu D., Jones B.A., Roeder P.L., Admassu B., Klooster G.G.V. (2012). Rinderpest Eradication: Appropriate Technology and Social Innovations. Science.

[563] Butler D. (2013). Rinderpest Research Restarts. Nat. Cell Biol..

[564] Butler D. (2019). Sequence and Destroy: The Quest to Eliminate the Last Stocks of Deadly Rinderpest Virus. Nat. Cell Biol..

[565] Hamilton K., Visser D., Evans B., Vallat B. (2015). Identifying and Reducing Remaining Stocks of Rinderpest Virus. Emerg. Infect. Dis..

[566] Fournié G., Jones B.A., Beauvais W., Lubroth J., Njeumi F., Cameron A., Pfeiffer D.U. (2014). The Risk of Rinderpest Re-Introduction in Post-Eradication Era. Prev. Vet. Med..

[567] Hamilton K., Baron M., Matsuo K., Visser D. (2017). Rinderpest Eradication: Challenges for Remaining Disease Free and Implications for Future Eradication Efforts. Rev. Sci. Tech..

[568] King S., Rajko-Nenow P., Ropiak H.M., Ribeca P., Batten C., Baron M.D. (2020). Full Genome Sequencing of Archived Wild Type and Vaccine Rinderpest Virus Isolates Prior to Their Destruction. Sci. Rep..

[569] The Lancet Infectious Diseases (2019). Rinderpest, Smallpox, and the Imperative of Destruction. Lancet Infect. Dis..

[570] Kurosawa S., Kobune F., Okuyama K., Sugiura A. (1987). Effects of Antipyretics in Rinderpest Virus Infection in Rabbits. J. Infect. Dis..

[571] Yoneda M., Bandyopadhyay S.K., Shiotani M., Fujita K., Nuntaprasert A., Miura R., Baron M.D., Barrett T., Kai C. (2002). Rinderpest Virus H Protein: Role in Determining Host Range in Rabbits. J. Gen. Virol..

[572] Rahman M., Shaila M.S., Gopinathan K.P. (2003). Baculovirus Display of Fusion Protein of Peste des Petits Ruminants Virus and Hemagglutination Protein of Rinderpest Virus and Immunogenicity of the Displayed Proteins in Mouse Model. Virology.

[573] Galbraith S.E., McQuaid S., Hamill L., Püllen L., Barrett T., Cosby S.L. (2002). Rinderpest and Peste des Petits Ruminants Viruses Exhibit Neurovirulence in Mice. J. Neurovirol..

[574] Khandelwal A., Renukaradhya G.J., Rajasekhar M., Sita G.L., Shaila M.S. (2004). Systemic and Oral Immunogenicity of Hemagglu-Tinin Protein of Rinderpest Virus Expressed by Transgenic Peanut Plants in a Mouse Model. Virology.

[575] James A.D., Rossiter P.B. (1989). An Epidemiological Model of Rinderpest. I. Description of the Model. Trop. Anim. Health Prod..

[576] Manore C., McMahon B., Fair J., Hyman J.M., Brown M., LaBute M. (2011). Disease Properties, Geography, and Mitigation Strategies in a Simulation Spread of Rinderpest across the United States. Vet. Res..

[577] Ortiz-Pelaez A., Pfeiffer D.U., Tempia S., Otieno F.T., Aden H.H., Costagli R. (2010). Risk Mapping of Rinderpest Sero-Prevalence in Central and Southern Somalia Based on Spatial and Network Risk Factors. BMC Vet. Res..

[578] Tillé A., Lefèvre C., Pastoret P.-P., Thiry E. (1991). A Mathematical Model of Rinderpest Infection in Cattle Populations. Epidemiol. Infect..

[579] Mariner J., McDermott J., Heesterbeek J., Catley A., Roeder P. (2005). A Model of Lineage-1 and Lineage-2 Rinderpest Virus Transmission in Pastoral Areas of East Africa. Prev. Vet. Med..

[580] Mourant J.R., Fenimore P.W., Manore C.A., McMahon B.H. (2018). Decision Support for Mitigation of Livestock Disease: Rinderpest as a Case Study. Front. Vet. Sci..

[581] Holzer B., Hodgson S., Logan N., Willett B., Baron M.D. (2016). Protection of Cattle against Rinderpest by Vaccination with Wild-Type but Not Attenuated Strains of Peste des Petits Ruminants Virus. J. Virol..

[582] De Swart R.L., Duprex W.P., Osterhaus A. (2012). Rinderpest Eradication: Lessons for Measles Eradication?. Curr. Opin. Virol..

[583] Thomson G.R., Penrith M.L. (2017). Eradication of Transboundary Animal Diseases: Can the Rinderpest Success Story be Repeated?. Transbound. Emerg. Dis..

[584] Mukhopadhyay A.K., Taylor W.P., Roeder P.L. (1999). Rinderpest: A Case Study of Animal Health Emergency Management. Rev. Sci. Tech..

[585] Rao T.V., Bandyopadhyay S.K. (2000). A Comprehensive Review of Goat Pox and Sheep Pox and Their Diagnosis. Anim. Health Res. Rev..

[586] Ben Chehida F., Ayari-Fakhfakh E., Caufour P., Amdouni J., Nasr J., Messaoudi L., Ammar H.H., Sghaier S., Bernard C., Ghram A. (2018). Sheep Pox in Tunisia: Current Status and Perspectives. Transbound. Emerg. Dis..

[587] European Food Safety (2014). A Scientific Opinion on Sheep and Goat Pox. EFSA J..

[588] Bhanuprakash V., Venkatesan G., Balamurugan V., Hosamani M., Yogisharadhya R., Chauhan R.S., Pande A., Mondal B., Singh R.K. (2010). Pox Outbreaks in Sheep and Goats at Makhdoom (Uttar Pradesh), India: Evidence of Sheeppox Virus Infection in Goats. Transbound. Emerg. Dis..

[589] Yune N., Abdela N. (2017). Epidemiology and Economic Importance of Sheep and Goat Pox: A Review on Past and Current Aspects. J. Vet. Sci. Technol..

[590] Mirzaie K., Barani S.M., Bokaie S. (2015). A Review of Sheep Pox and Goat Pox: Perspective of Their Control and Eradication in Iran. J. Adv. Vet. Anim. Res..

[591] Malesios C., Kostoulas P., Dadousis K., Demiris N. (2017). An Early Warning Indicator for Monitoring Infectious Animal Diseases and Its Application in the Case of a Sheep Pox Epidemic. Stoch. Environ. Res. Risk Assess..

[592] Aregahagn S., Tadesse B., Tegegne B., Worku Y., Mohammed S. (2021). Spatiotemporal Distributions of Sheep and Goat Pox Disease Outbreaks in the Period. 2013–2019 in Eastern Amhara Region, Ethiopia. Vet. Med. Int..

[593] Babiuk S., Bowden T.R., Boyle D.B., Wallace D.B., Kitching R.P. (2008). Capripoxviruses: An Emerging Worldwide Threat to Sheep, Goats and Cattle. Transbound. Emerg. Dis..

[594] Gitao C.G., Mbindyo C., Omani R., Chemweno V. (2017). Review of Sheep Pox Disease in Sheep. J. Vet. Med. Res..

[595] Balinsky C.A., Delhon G., Smoliga G., Prarat M., French R.A., Geary S.J., Rock D.L., Rodriguez L.L. (2008). Rapid Preclinical Detection of Sheeppox Virus by a Real-Time PCR Assay. J. Clin. Microbiol..

[596] Bhanuprakash V., Indrani B.K., Hosamani M., Singh R.K. (2006). The Current Status of Sheep Pox Disease. Comp. Immunol. Microbiol. Infect. Dis..

[597] Bowden T.R., Coupar B.E., Babiuk S.L., White J.R., Boyd V., Duch C.J., Shiell B.J., Ueda N., Parkyn G.R., Copps J.S. (2009). Detection of Antibodies Specific for Sheeppox and Goatpox Viruses Using Recombinant Capripoxvirus Antigens in an Indirect Enzyme-Linked Immunosorbent Assay. J. Virol. Methods.

[598] Haegeman A., Zro K., Sammin D., Vandenbussche F., Ennaji M.M., de Clercq K. (2016). Investigation of a Possible Link Between Vaccination and the 2010 Sheep Pox Epizootic in Morocco. Transbound. Emerg. Dis..

[599] Hosamani M., Mondal B., Tembhurne P.A., Bandyopadhyay S.K., Singh R.K., Rasool T.J. (2004). Differentiation of Sheep Pox and Goat Poxviruses by Sequence Analysis and PCR-RFLP of P32 Gene. Virus Genes.

[600] Pestova Y., Byadovskaya O., Kononov A., Sprygin A. (2018). A Real Time High-Resolution Melting PCR Assay for Detection and Differentiation among Sheep Pox Virus, Goat Pox Virus, Field and Vaccine Strains of Lumpy Skin Disease Virus. Mol. Cell. Probes.

[601] Sumana K., Revanaiah Y., Shivachandra S.B., Mothay D., Apsana R., Saminathan M., Basavaraj S., Reddy G.B.M. (2020). Molecular Phylogeny of Capripoxviruses Based on Major Immunodominant Protein (P32) Reveals Circulation of Host Specific Sheeppox and Goatpox Viruses in Small Ruminants of India. Infect. Genet. Evol..

[602] Tian H., Chen Y., Wu J., Shang Y., Liu X. (2010). Serodiagnosis of Sheeppox and Goatpox Using an Indirect ELISA Based on Synthetic Peptide Targeting for the Major Antigen P32. Virol. J..

[603] Kardjadj M. (2017). Prevalence, Distribution, and Risk Factor for Sheep Pox and Goat Pox (SPGP) in Algeria. Trop. Anim. Health Prod..

[604] OIE (2019). Sheep Pox and Goat Pox. Manual of Diagnostic Tests and Vaccines for Terrestrial Animals.

[605] Boumart Z., Daouam S., Belkourati I., Rafi L., Tuppurainen E., Tadlaoui K.O., el Harrak M. (2016). Comparative Innocuity and Efficacy of Live and Inactivated Sheeppox Vaccines. BMC Vet. Res..

[606] Tuppurainen E.S., Lamien C.E., Diallo A., Metwally S., El Idrissi A., Viljoen G. (2021). Capripox (Lumpy Skin Disease, Sheep Pox, and Goat Pox). Veterinary Vaccines.

[607] Kali K., Kardjadj M., Touaghit N., Yahiaoui F., Ben-Mahdi M.H. (2019). Understanding the Epidemiology of Sheep-Pox Outbreaks among Vaccinated Algerian Sheep and Post Vaccination Evaluation of the Antibodies Kinetics of the Commercially Used Vaccine. Comp. Immunol. Microbiol. Infect. Dis..

[608] Boshra H., Truong T., Nfon C., Gerdts V., Tikoo S., Babiuk L.A., Kara P., Mather A., Wallace D., Babiuk S. (2013). Capripoxvirus-Vectored Vaccines against Livestock Diseases in Africa. Antiviral Res..

[609] Fakri F., Ghzal F., Daouam S., Elarkam A., Douieb L., Zouheir Y., Tadlaoui K.O., Fassi-Fihri O. (2015). Development and Field Application of a New Combined Vaccine against Peste des Petits Ruminants and Sheep Pox. Trials Vaccinol..

[610] Didarkhah M., Vatandoost M., Dirandeh E., Davachi N.D. (2019). Characterization and Pattern of Culling in Goats. Arch. Razi. Inst..

[611] Garner M.G., Sawarkar S.D., Brett E.K., Edwards J.R., Kulkarni V.B., Boyle D.B., Singh S.N. (2000). The Extent and Impact of Sheep Pox and Goat Pox in the State of Maharashtra, India. Trop. Anim. Health Prod..

[612] Limon G., Gamawa A.A., Ahmed A.I., Lyons N.A., Beard P.M. (2020). Epidemiological Characteristics and Economic Impact of Lumpy Skin Disease, Sheeppox and Goatpox Among Subsistence Farmers in Northeast Nigeria. Front. Vet. Sci..

[613] Pham T.H., Lila M.A.M., Rahaman N.Y.A., Lai H.L.T., Nguyen L.T., Do K.V., Noordin M.M. (2020). Epidemiology and Clinico-Pathological Characteristics of Current Goat Pox Outbreak in North Vietnam. BMC Vet. Res..

[614] Chamchod F. (2018). Modeling the Spread of Capripoxvirus Among Livestock and Optimal Vaccination Strategies. J. Theor. Biol..

[615] Singh B., Prasad S. (2008). Modelling of Economic Losses due to Some Important Diseases in Goats in India. Agric. Econ. Res. Rev..

[616] Zhuravlyova V.A., Lunitsin A.V., Kneize A.V., Guzalova A.G., Balyshev V.M. (2020). Epizootic Situation and Modeling of Potential Nosoareals of Peste des Petits Ruminants, Sheep and Goat Pox and Rift Valley Fever Up to 2030. Agric. Biol..

[617] Chervyakova O.V., Zaitsev V.L., Iskakov B.K., Tailakova E.T., Strochkov V.M., Sultankulova K.T., Sandybayev N.T., Stanbekova G.E., Beisenov D.K., Abduraimov Y.O. (2016). Recombinant Sheep Pox Virus Proteins Elicit Neutralizing Antibodies. Viruses.

[618] Hamdi J., Bamouh Z., Jazouli M., Boumart Z., Tadlaoui K.O., Fihri O.F., el Harrak M. (2020). Experimental Evaluation of the Cross-Protection between Sheeppox and Bovine Lumpy Skin Vaccines. Sci. Rep..

[619] Kitching R.P. (1986). Passive Protection of Sheep against Capripoxvirus. Res. Vet. Sci..

[620] Kitching R.P., Taylor W.P. (1985). Clinical and Antigenic Relationship between Isolates of Sheep and Goat Pox Viruses. Trop. Anim. Health Prod..

[621] Zhugunissov K., Bulatov Y., Orynbayev M., Kutumbetov L., Abduraimov Y., Shayakhmetov Y., Taranov D., Amanova Z., Mambetaliyev M., Absatova Z. (2020). Goatpox Virus (G20-LKV) Vaccine Strain Elicits a Protective Response in Cattle against Lumpy Skin Disease at Challenge with Lumpy Skin Disease Virulent Field Strain in a Comparative Study. Vet. Microbiol..

[622] Plowright W., MacLeod W.G., Ferris R.D. (2012). The Pathogenesis of Sheep Pox in the Skin of Sheep. J. Comp. Pathol..

[623] Wolff J., el Rahman S.A., King J., El-Beskawy M., Pohlmann A., Beer M., Hoffmann B. (2020). Establishment of a Challenge Model for Sheeppox Virus Infection. Microorganisms.

[624] Bowden T.R., Babiuk S.L., Parkyn G.R., Copps J.S., Boyle D.B. (2008). Capripoxvirus Tissue Tropism and Shedding: A Quantitative Study in Experimentally Infected Sheep and Goats. Virology.

[625] Brocchi E., Zhang G., Knowles N.J., Wilsden G., McCauley J.W., Marquardt O., Ohlinger V.F., de Simone F. (1997). Molecular Epidemiology of Recent Outbreaks of Swine Vesicular Disease: Two Genetically and Antigenically Distinct Variants in Europe, 1987–1994. Epidemiol. Infect..

[626] Lin F., Kitching R.P. (2000). Swine Vesicular Disease: An Overview. Vet. J..

[627] Tamba M., Plasmati F., Brocchi E., Ruocco L. (2020). Eradication of Swine Vesicular Disease in Italy. Viruses.

[628] (2012). EFSA Panel Animal Health and Welfare (AHAW) Scientific Opinion on Swine Vesicular Disease and Vesicular Stomatitis. EFSA J..

[629] Terpstra C. (1992). Vesicular Swine Disease in The Netherlands. Tijdschr Diergeneeskd.

[630] Dekker A., Moonen P., de Boer-Luijtze E.A., Terpstra C. (1995). Pathogenesis of Swine Vesicular Disease after Exposure of Pigs to an Infected Environment. Vet. Microbiol..

[631] Dibaba A.B. (2019). The Risk of Introduction of Swine Vesicular Disease Virus into Kenya via Natural Sausage Casings Imported from Italy. Prev. Vet. Med..

[632] Yang M., Gagliardi K., McIntyre L., Xu W., Goolia M., Ambagala T., Brocchi E., Grazioli S., Hooper–McGrevy K., Nfon C. (2020). Development and Evaluation of Swine Vesicular Disease Isotype-Specific Antibody Elisas Based on Recombinant Virus-Like Particles. Transbound. Emerg. Dis..

[633] Brocchi E., Berlinzani A., Gamba D., de Simone F. (1995). Development of Two Novel Monoclonal Antibody-Based Elisas for the Detection of Antibodies and the Identification of Swine Isotypes against Swine Vesicular Disease Virus. J. Virol. Methods.

[634] Escribano-Romero E., Jimenez-Clavero M.A., Gomes P., García-Ranea J.A., Ley V. (2004). Heparan Sulphate Mediates Swine Vesicular Disease Virus Attachment to the Host Cell. 2004, 85, 653–663. J. Gen. Virol..

[635] Fry E.E., Knowles N.J., Newman J.W.I., Wilsden G., Rao Z., King A.M.Q., Stuart D.I. (2003). Crystal Structure of Swine Vesicular Disease Virus and Implications for Host Adaptation. J. Virol..

[636] Martín-Acebes M.A., González-Magaldi M., Rosas M.F., Borrego B., Brocchi E., Armas-Portela R., Sobrino F. (2008). Subcellular Distribution of Swine Vesicular Disease Virus Proteins and Alterations Induced in Infected Cells: A Comparative Study with Foot-and-Mouth Disease Virus and Vesicular Stomatitis Virus. Virology.

[637] Burrows R., Greig A., Goodridge D. (1973). Swine Vesicular Disease. Res. Vet. Sci..

[638] Chu R.M., Moore D.M., Conroy J.D. (1979). Experimental Swine Vesicular Disease, Pathology and Immunofluorescence Studies. Can. J. Comp. Med..

[639] Lin F., Mackay D.K.J., Knowles N.J. (1998). The Persistence of Swine Vesicular Disease Virus Infection in Pigs. Epidemiol. Infect..

[640] Mann J.A., Hutchings G.H. (1980). Swine Vesicular Disease: Pathways of Infection. J. Hyg..

[641] Mowat G.N., Prince M.J., Spier R.E., Staple R.F. (1974). Preliminary Studies on the Development of a Swine Vesicular Disease Vaccine. Arch. Gesamte Virusforsch.

[642] Letchworth G.J., Rodriguez L.L., del Cbarrera J. (1999). Vesicular Stomatitis. Vet. J..

[643] Whelan S.P.J., Mahy B.W.J., Van Regenmortel M.H.V. (2008). Vesicular Stomatitis Virus. Encyclopedia of Virology.

[644] Vanleeuwen J.A., Rodriguez L.L., Waltner-Toews D. (1995). Cow, Farm, and Ecologic Risk Factors of Clinical Vesicular Stomatitis on Costa Rican Dairy Farms. Am. J. Trop. Med. Hyg..

[645] Webb P.A., Monath T.P., Reif J.S., Smith G.C., Kemp G.E., Lazuick J.S., Walton T.E. (1987). Epizootic Vesicular Stomatitis in Colorado, 1982: Epidemiologic Studies along the Northern Colorado Front Range. Am. J. Trop. Med. Hyg..

[646] Webb P.A., Holbrook F.R., Monath T.P. (1989). Vesicular Stomatitis. The Arboviruses: Epidemiology and Ecology.

[647] Fultz P.N., Holland J.J. (1985). Differing Responses of Hamsters to Infection by Vesicular Stomatitis Virus Indiana and New Jersey Serotypes. Virus Res..

[648] Gresser I., Tovey M.G., Bourali-Maury C. (1975). Efficacy of Exogenous Interferon Treatment Initiated after Onset of Multiplication of Vesicular Stomatitis Virus in the Brains of Mice. J. Gen. Virol..

[649] Huneycutt B.S., Plakhov I.V., Shusterman Z., Bartido S.M., Huang A., Reiss C.S., Aoki C. (1994). Distribution of Vesicular Stomatitis Virus Proteins in the Brains Of BALB/C Mice Following Intranasal Inoculation: An Immunohistochemical Analysis. Brain Res..

[650] Leder R.R., Maas J., Lane V.M., Evermann J.F. (1983). Epidemiologic Investigation of Vesicular Stomatitis in a Dairy and Its Economic Impact. Bov. Pract..

[651] Clarke D.K., Hendry R.M., Singh V., Rose J.K., Seligman S.J., Klug B., Kochhar S., Mac L.M., Carbery B., Chen R.T. (2016). Live Virus Vaccines Based on a Vesicular Stomatitis Virus (VSV) Backbone: Standardized Template with Key Considerations for a Risk/Benefit Assessment. Vaccine.

[652] Campbell Z., Coleman P., Guest A., Kushwaha P., Ramuthivheli T., Osebe T., Perry B., Salt J. (2021). Prioritizing Smallholder Animal Health Needs in East. Africa, West. Africa, and South. Asia Using Three Approaches: Literature Review, Expert Workshops, and Practitioner Surveys. Prev. Vet. Med..

[653] Hopker A., Pandey N., Saikia D., Goswami J., Hopker S., Saikia R., Sargison N. (2019). Spread and Impact of Goat Pox (“Sagolay Bohonta”) in a Village Smallholder Community around Kaziranga National Park, Assam, India. Trop. Anim. Health Prod..

[654] Holzmann H., Hengel H., Tenbusch M., Doerr H.W. (2016). Eradication of Measles: Remaining Challenges. Med. Microbiol. Immunol..

[655] (2002). Food and Drug Administration (FDA) New Drug and Biological Drug Products; Evidence Needed to Demonstrate Effectiveness of New Drugs when Human Efficacy Studies Are Not Ethical or Feasible. Final Rule Fed. Regist..

[656] Gronvall G.K., Trent D., Borio L., Brey R., Nagao L. (2007). The FDA Animal Efficacy Rule and Biodefense. Nat. Biotechnol..

[657] Domingo E., Perales C. (2019). Viral Quasispecies. PLoS Genet..

